# Transcriptional Dysregulation in *NIPBL* and Cohesin Mutant Human Cells

**DOI:** 10.1371/journal.pbio.1000119

**Published:** 2009-05-26

**Authors:** Jinglan Liu, Zhe Zhang, Masashige Bando, Takehiko Itoh, Matthew A. Deardorff, Dinah Clark, Maninder Kaur, Stephany Tandy, Tatsuro Kondoh, Eric Rappaport, Nancy B. Spinner, Hugo Vega, Laird G. Jackson, Katsuhiko Shirahige, Ian D. Krantz

**Affiliations:** 1 Division of Human Genetics, Abramson Research Institute, The Children's Hospital of Philadelphia, Philadelphia, Pennsylvania, United States of America; 2 Center for Biomedical Informatics, The Children's Hospital of Philadelphia, Philadelphia, Pennsylvania, United States of America; 3 Laboratory of Chromosome Structure and Function, Department of Biological Science, Graduate School of Bioscience and Biotechnology, Tokyo Institute of Technology, Yokohama City, Kanagawa, Japan; 4 The University of Pennsylvania School of Medicine, Philadelphia, Pennsylvania, United States of America; 5 Division of Developmental Disability, Misakaenosono Mutsumi Developmental, Medical, and Welfare Center, Isahaya, Japan; 6 NAPCORE, The Children's Hospital of Philadelphia, Philadelphia, Pennsylvania, United States of America; 7 Instituto de Genética, Universidad Nacional de Colombia, Bogotá, Colombia; 8 Department of Obstetrics and Gynecology, Drexel University School of Medicine, Philadelphia, Pennsylvania, United States of America; MRC Human Genetics Unit, United Kingdom

## Abstract

Cohesin regulates sister chromatid cohesion during the mitotic cell cycle with Nipped-B-Like (NIPBL) facilitating its loading and unloading. In addition to this canonical role, cohesin has also been demonstrated to play a critical role in regulation of gene expression in nondividing cells. Heterozygous mutations in the cohesin regulator *NIPBL* or cohesin structural components *SMC1A* and *SMC3* result in the multisystem developmental disorder Cornelia de Lange Syndrome (CdLS). Genome-wide assessment of transcription in 16 mutant cell lines from severely affected CdLS probands has identified a unique profile of dysregulated gene expression that was validated in an additional 101 samples and correlates with phenotypic severity. This profile could serve as a diagnostic and classification tool. Cohesin binding analysis demonstrates a preference for intergenic regions suggesting a *cis*-regulatory function mimicking that of a boundary/insulator interacting protein. However, the binding sites are enriched within the promoter regions of the dysregulated genes and are significantly decreased in CdLS proband, indicating an alternative role of cohesin as a transcription factor.

## Abstract 2

Genome-wide studies using cells from patients with Cornelia de Lange Syndrome reveal a role for cohesin in regulating gene expression in human cells.

## Author Summary

Appropriate segregation of chromosomes to daughter cells depends upon proper cohesion of sister chromatids during mitosis. The multiprotein cohesin complex and its regulators are key factors in this process. Intriguingly, recent work has shown that the cohesin complex also has other cellular roles, including a role in regulating gene expression. Additionally, mutations in cohesin structural and regulatory components have been linked to human multisystem developmental disorders such as Cornelia de Lange Syndrome (CdLS), but the role cohesin is playing in the pathogenesis of this disorder is unknown. To define the role that cohesin plays in regulating gene expression in human cells, we analyzed gene expression and genome-wide cohesin binding patterns in cells from normal subjects and from CdLS probands with mutations in the cohesin regulator *NIPBL* or in the cohesin structural component *SMC1A*. We found a strikingly conserved pattern of gene dysregulation in these different cell lines that correlates with disease severity and a significant correlation between gene dysregulation and cohesin binding around misexpressed genes. The observed pattern of binding and misexpression is consistent with cohesin having a putative role as a boundary/insulator interacting protein or transcription factor, the activity of which is disrupted in CdLS probands.

## Introduction

Cohesin is an evolutionally conserved multisubunit protein complex consisting of an SMC1A and SMC3 heterodimer, and at least two non-SMC proteins SCC1 (also known as RAD21 or MCD1) and SCC3 (also known as SA or STAG). Cohesin controls sister chromatid cohesion during S phase with Nipped-B-Like (NIPBL) facilitating its loading and unloading [1]. ESCO2 possesses acetyltransferase activity and is involved in the establishment of cohesion [2]. Cohesin is loaded onto chromatin during G1/S phase in budding yeast and during telophase of the preceding cell division in vertebrates. Loading of cohesin also happens during G2/M phase when double strand DNA breaks are generated [3]. Removal of cohesin from chromosome arms begins during prophase and completes by separase-mediated dissolving of the remaining cohesin from centromeres during anaphase [3]. Although no consensus DNA sequence for cohesin binding has been demonstrated, cohesin is enriched at heterochromatin [4] and DNA double-strand breaks (DSBs) [5]. A large amount of intact and free cohesin is associated with chromosomes for most of the cell division cycle because of a separase independent mechanism [6]–[8]. A noncanonical role for cohesin as a key regulator of gene expression has been proposed [9]. The *Drosophila* NIPBL homolog, Nipped-B, alleviates the *gypsy* insulator function by assisting in long distance enhancer–promoter interactions to activate *cut* and *Ultrabithorax* expression. Nipped-B and cohesin colocalize and bind preferentially to active transcription units [9]. Recently, CTCF was reported to colocalize with cohesin and required for cohesin binding to chromatin [10],[11]. CTCF is the only protein known to bind to all vertebrate chromatin insulators and was initially identified as a transcription factor that binds to mammalian c-*MYC* promoters [12]. In addition, CTCF is well studied for its role in regulating genomic imprinting, and is proposed to regulate higher order chromatin structures such as intra- and interchromosomal association [13],[14]. CTCF is required for *Tsix* transactivation and involved in maintaining both X inactivation and escape domains, it stabilizes the repetitive sequences in several genetic disorders, and has been suggested to act as a tumor suppressor gene [15]. CTCF binding sites have been mapped in the human genome [16].

Cornelia de Lange syndrome (CdLS, Online Mendelian Inheritance in Man [OMIM] 122470, 300590, and 610759) is a dominant disorder with multisystem abnormalities including characteristic facial features, hirsutism, upper extremity defects, gastroesophageal dysfunction, growth, and neurodevelopmental delays. The incidence is about one in 10,000, with most cases being sporadic. There is equal gender distribution with a high degree of phenotypic variability. About 60% of the probands with CdLS have heterozygous mutations in the *NIPBL* gene, whereas 5% have mutations in the *SMC1A* gene, and one patient was found to have a mutation in the *SMC3* gene [17],[18]. Other multisystem developmental disorders have been found to be caused by mutations in cohesin-related genes, such as Roberts-SC phocomelia (RBS, OMIM 268300) an autosomal recessive disorder caused by either homozygous or compound heterozygous mutations in the *ESCO2* gene [19]. These disorders have collectively been termed “cohesinopathies.” Given the paucity of sister chromatid cohesion defects observed in individuals with CdLS [20], we hypothesize that it is the newly established role of cohesin in gene regulation that results in the multisystem phenotype when disrupted. To study the effects of disruption of cohesin on gene expression in human cells we have utilized lymphoblastoid cell lines (LCLs) from individuals with CdLS that harbor known heterozygous mutations in the cohesin regulator NIPBL and cohesin structural component SMC1A and applied a genome-wide approach to analyze gene transcription and cohesin binding.

## Results

### Specific Genes Are Differentially Expressed in CdLS Probands with *NIPBL* Mutations

**Figure 1:**
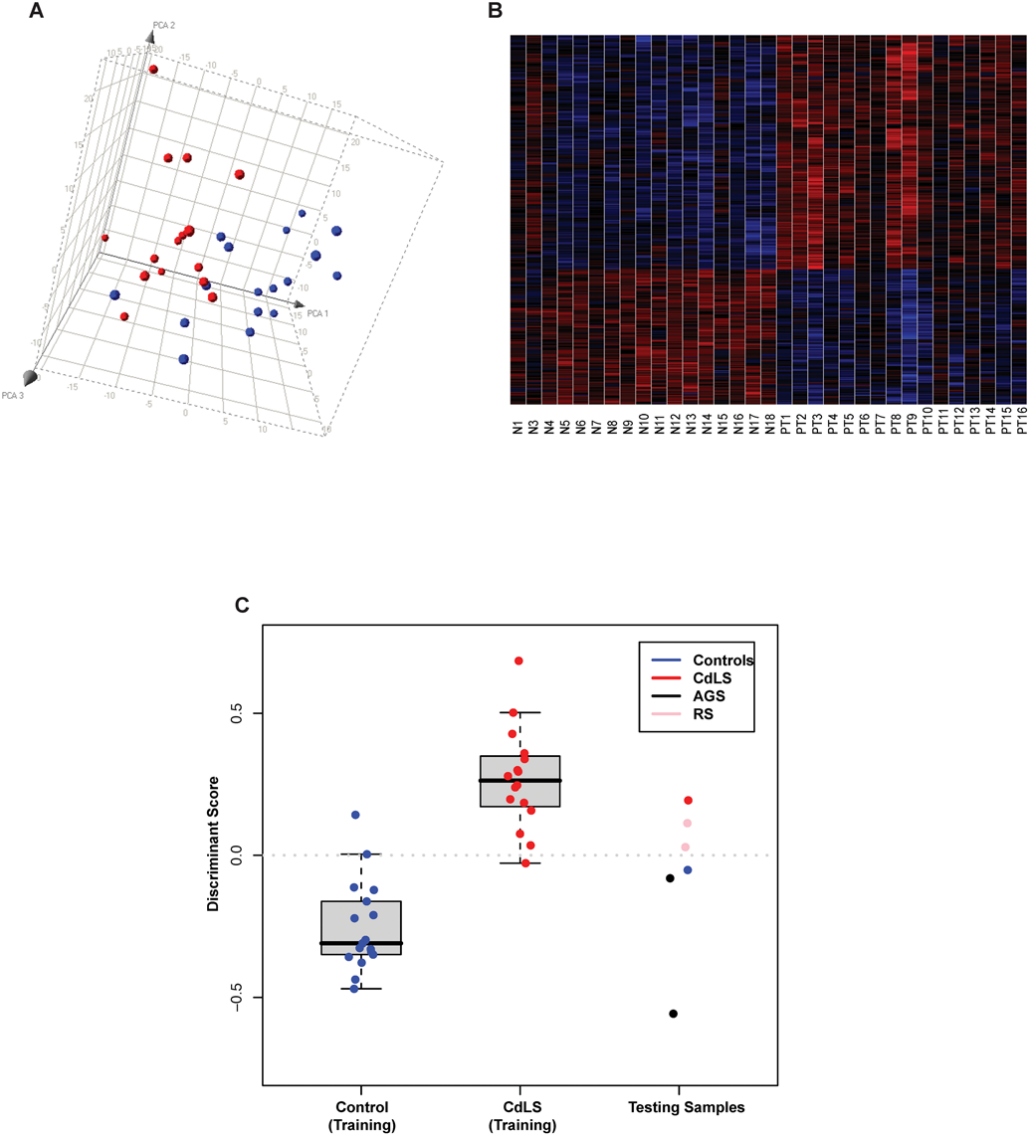
Classifications of the 33 training samples by gene expression. (A) Unsupervised clustering of 17 healthy controls (blue dots) and 16 severe CdLS probands (red dots) by principle component analysis (PCA) of the 27,995 probe sets actively transcribed in LCLs. The separation between the training groups indicates that controls and probands have different gene expression patterns. (B) Heatmap showing that the identified 420 probe sets (FDR<0.01) are expressed dramatically differently between CdLS probands (PT) and healthy controls (N). Red represents genes that are upregulated and blue represents genes that are downregulated. The left 17 columns represent control samples, and the right 16 columns represent proband samples. Rows display gene expression levels. (C) Nearest centroid classifications of the 33 training samples and six testing samples. Among the training samples, two healthy controls and one CdLS proband were misclassified after Leave-One-Out cross validation. Among the testing samples, CdLS probands and two RBS probands were classified into CdLS group whereas the one healthy control and two probands with AGS were classified into the control group.

LCLs from 16 severely affected CdLS probands with *NIPBL* protein-truncating mutations as well as 17 age, gender, and race matched healthy controls were used as training samples for assays on the Affymetrix HG-U133 plus 2.0 expression arrays, six additional individuals including one CdLS proband, one healthy control, two RBS probands (a related cohesinopathy), and two Alagille syndrome (AGS) probands (an unrelated multisystem dominant developmental disorder caused by disruption in the Notch signaling pathway) served as testing samples (Table S1A and S1B). 27,995 probe sets (12,740 nonredundant genes) were considered to be expressed in human LCLs. Unsupervised sample clustering by principle component analysis (PCA) of all the expressed probe sets was able to separate probands from controls in the training set indicating these two groups have different gene expression patterns (Figure 1A). Differential expression of these 27,995 probe sets was ranked by *F* = (between group variance)/(within group variance). Permutation analysis was performed 100 times and false discovery rate (FDR) was calculated for each *F* score, whereas redundancy was collapsed by keeping the ones with the highest *F* scores. We have identified a group of 1,915 probe sets (1,501 nonredundant genes) with FDR<0.05 and 420 probe sets (339 nonredundant genes) with FDR<0.01 (Tables S2 and S3) that are differentially expressed in CdLS. Heatmap representation of the expression levels of these genes clearly demonstrates that the expression of the 420 probe sets is remarkably different between CdLS probands and controls (Figure 1B). *NIPBL* itself had the highest ranking, with FDR = 0 and a fold change of −1.34.

In order to examine whether CdLS probands could be differentiated from controls through expression profiling, Leave-One-Out cross validation was performed on the training set. The top 400 probe sets were selected on each of the 33 rounds that corresponded to an FDR≈0.01. The left-out samples were successfully classified into two distinct groups using nearest centroid method with the exception of two controls and one proband that were misclassified (Figure 1C). The area under the receiver operating characteristic (ROC) curve is 0.985 with test accuracy of 91% (95% confidence interval = 76%–98%). Nearest centroid classification method was further performed on the six testing samples based on the identified 420 probe sets (FDR<0.01). The one healthy control and two individuals with AGS were classified as controls; one CdLS and two RBS probands were classified as probands (Figure 1C and Table S4). RBS is due to the mutations in the *ESCO2* gene that also regulates cohesin, whereas AGS is an independent genetic disorder due primarily to mutations in the *JAG1* gene, a member of the Notch signaling pathway. Thus, although limited to only two samples, it appears that RBS shares a similar transcription profile with CdLS, consistent with these two disorders being caused by disruption of the cohesin pathway. It is of interest that whereas the two RBS probands were classified as CdLS, their discriminant scores (DS) were actually midway between the scores of CdLS probands and the controls suggesting RBS might have an intermediate transcription profile to CdLS and controls (Figure 1C).

### Specific Gene Expression Is Tightly Associated with Phenotypic Severity among Different Groups of CdLS Probands

Clustering-based feature selection was carried out on the 339 nonredundant genes (420 probe sets, FDR<0.01) to identify independent pathways or functional groups. Five clusters were discovered (Table S5) and 32 genes (Table S6) were chosen for further custom array validation according to smaller FDR, bigger fold change, and less redundancy.

**Figure 2:**
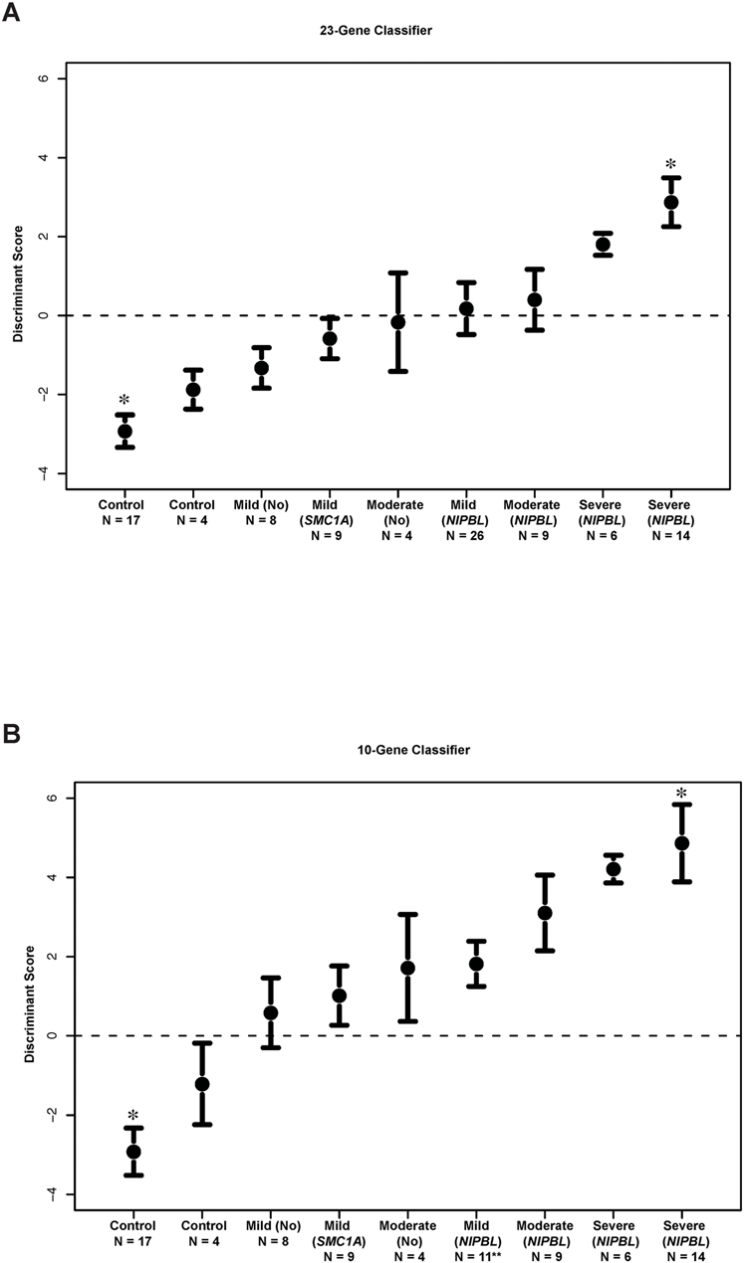
Classifier genes are identified for CdLS. Clear progression of discriminant score (DS) from low to high is correlated with the phenotype from unaffected → mild → moderate → severely affected with CdLS. (A) The 23-gene classifier separates CdLS probands with *NIPBL* mutations from the rest of the individuals. Healthy controls, probands with other genetic disorders, CdLS probands with *SMC1A* mutations, and CdLS probands with no gene mutation identified are distinctly separated from each other in a progressive manner correlated with phenotypic severity. (B) The ten-gene classifier differentially categorizes all CdLS probands from non-CdLS individuals and plots correlate to the severity of the CdLS probands. Healthy controls are labeled as “Control,” disease severity is described as “Mild,” “Moderate,” and “Severe” CdLS probands with *NIPBL* mutations, *SMC1A* mutations or no identified gene mutation are labeled as “*NIPBL*,” “*SMC1A*,” or “No,” respectively. *, training samples; **, number of mild cases with an *NIPBL* mutation was reduced from 26 in (A) to 11 in (B) with the other 15 cases having been used as training samples.

A cohort of 101 samples including individuals with different phenotypes (healthy, CdLS, or other disorders) and various gene mutations (Table S7) were measured on custom arrays carrying 56 probes mapped to the 32 selected genes (Tables S6 and S8). We have followed a step-wise procedure to identify classifiers according to different CdLS subgroups, and applied nearest centroid classifications on all 101 samples (Table S9A and S9B). Detailed analysis is described in Text S1. A 23-gene classifier can be used to categorize CdLS probands with *NIPBL* mutations from the rest of the samples including non-CdLS, CdLS probands with *SMC1A* mutations, and CdLS probands without an identifiable gene mutation. This indicates that the expression of these 23 genes is tightly correlated to *NIPBL* function. To improve the generality of the classifier, we randomly selected 15 mild probands with *NIPBL* mutations as a new training group. Expression of ten of the 23 genes was significantly different between this group and the original 17 controls and was also capable of subclassifying all CdLS probands from non-CdLS controls, regardless of the gene mutations or clinical presentations of the probands. This suggests that expression of these ten genes is affected by a CdLS specific disease process. For both classifications, the expression levels of the classifier genes are tightly correlated to disease severity. A clear progression of increasing discriminant scores (DS) can be seen from healthy controls through mild, moderate, and severe CdLS probands (Figure 2A and 2B). In addition, we have identified three genes *NFATC2*, *PAPSS2*, and *ZNF608* that could be used as biomarkers for CdLS (Figure S1A and S1B). The phenotype associated gene expression profiles strongly suggest either direct or indirect roles for the identified genes.

### Cohesin Binding Is Involved in Gene Expression in Human Cells

Cohesin is a multisubunit complex constructed from SMC1A, SMC3, RAD21, and SCC3 subunits. Mutations in *SMC1A* or *SMC3* and the cohesin regulator *NIPBL* lead to the human developmental disorder CdLS. To test the hypothesis that cohesin regulates transcription through its chromatin binding activity and that this association is regulated by NIPBL activity we undertook whole genome mapping of cohesin binding sites in LCLs from two healthy controls and one severely affected CdLS proband with an *NIPBL* mutation. Because of our inability to identify an effective antibody with high specificity against NIPBL or SMC1A, we chose an antibody against RAD21 (one of the other key components of the cohesin complex) to map genome-wide cohesin binding sites. Chromatin immunoprecipitation (ChIP) using a polyclonal antibody against human RAD21 was performed and products were hybridized on Affymatrix 2.0 tiling arrays. The score of model-based analysis of tiling-arrays algorithm (MAT) was calculated and probes were mapped to genomic positions. Peaks representing genomic regions bound by hRAD21 were identified with a *p*<10^−6^ and FDR<0.01.

**Figure 3:**
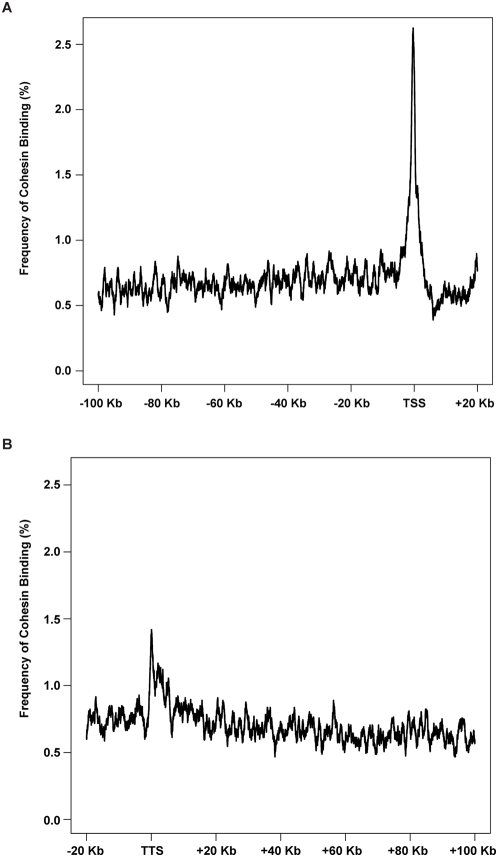
Cohesin binding analyzed in 15,162 unique transcripts demonstrates preferential binding to TSSs and TTSs. (A) The frequency of cohesin binding has a sharp peak around TSS and falls to the background level upstream of this peak. (B) The frequency of cohesin binding has another peak around TTS. The height of this peak is about half that of the peak height seen at TSS. Similarly the regions downstream of this peak have a cohesin binding frequency close to the background level.

**Figure 4:**
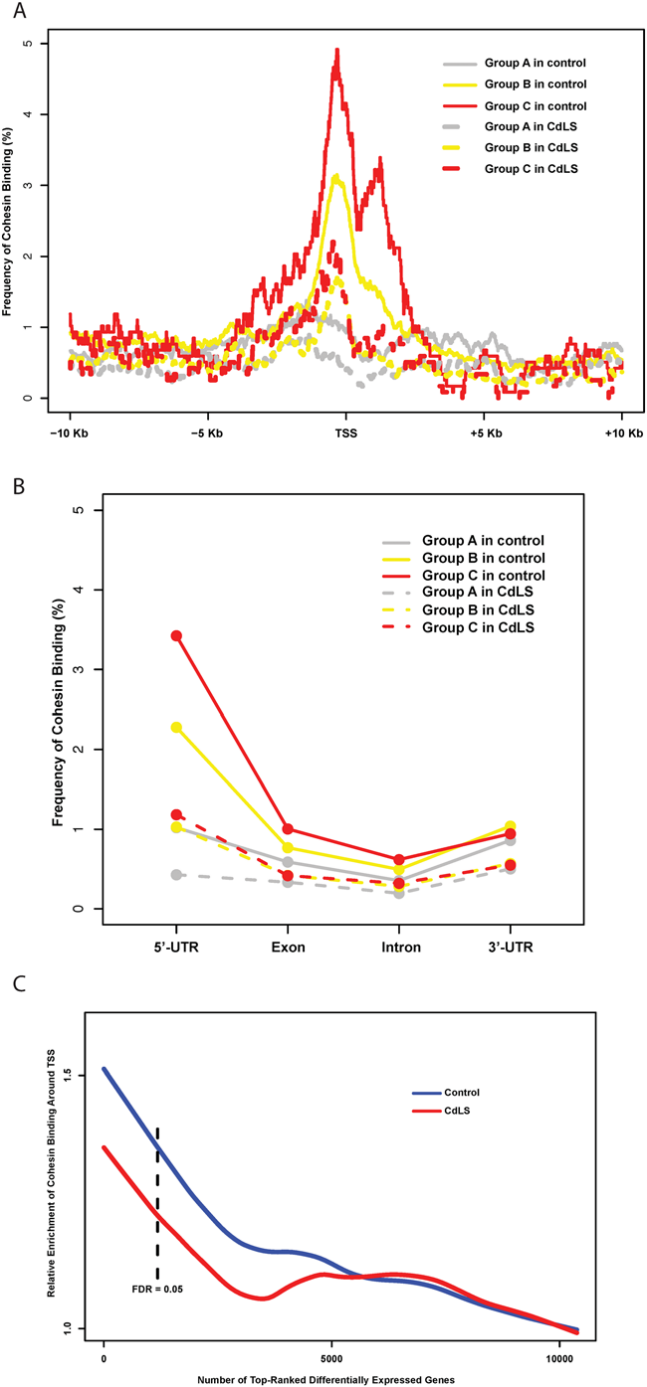
Frequency of cohesin binding around the TSS as related to transcriptional status in LCLs. Group A (silver), nontranscribed silent genes in LCLs (4,784 unique Refseq mRNAs); group B (yellow), genes without expression alterations between controls and CdLS probands (9,199 unique RefSeq mRNAs); group C (red), genes differentially expressed in CdLS (FDR < 0.05) (1,179 unique RefSeq mRNAs). (A) Frequency of cohesin binding at the TSS of group C genes is much lower in CdLS than in control. Group B genes have a moderate reduction, and group A genes have little change. Overall cohesin binding around the TSS is greatest for those genes that are actively transcribed in LCLs and especially in those genes that are misexpressed in CdLS. (B) Within the intragenic regions, 5′-UTRs of the actively transcribed genes (groups B and C) have higher cohesin binding frequency in control than other intragenic regions whereas group A genes have frequency close to the background level in all regions. In CdLS, the frequency dropped in all three gene groups in CdLS and the difference between different gene groups and regions tends to diminish. (C) Cohesin binding within 2 kb around TSS is enriched in differentially expressed genes. The 10,378 unique genes expressed in LCLs are ranked by their *F* scores. The reference enrichment is the percentage of genes having cohesin binding within 2 kb (+/− 1 kb) around TSS. The relative enrichment is calculated as the value of cohesin binding enrichment in top-ranked genes over the reference enrichment. The relative enrichment point is calculated for the total number of genes prior to the point on the *x*-axis. The numbers on *x*-axis denote the statistical ranks. The curves are smoothed by the LOWESS algorithm.

The 54,675 probe sets on Affymetrix HG-U133 plus 2.0 expression array can be unambiguously mapped to 15,162 unique RefSeq mRNAs including 10,378 transcribed and 4,784 nontranscribed genes in LCLs. 78% of the 15,162 mapped genes do not harbor intragenic cohesin sites (Here, “intragenic” means genomic region from the transcription start site [TSS] of a gene to the transcription termination site [TTS] of the same gene), and cohesin binds at variable distances outside those genes. 22% of the 15,162 mapped genes harbor intragenic cohesin sites, this number is reduced to 19.0% in the silent nontranscribed genes (*p*≤7.2e−6) and no change in the disease neutral genes (22.9%, *p*≤0.0864); on the contrary, more of the differentially expressed genes harbor cohesin sites (27.0%, *p*≤7.44e−5) (http://145.18.230.98/Service/Statistics/Binomial_proportions.html) (Table S10) suggesting a correlation between intragenic cohesin binding and gene expression. For the 22% of genes with intragenic cohesin sites, cohesin preferentially binds to a narrowed region surrounding the TSSs or the TTSs with frequency at the TTSs only half of that at the TSSs. The 100-kb regions spanning upstream and downstream of the genes have only background levels of cohesin binding (Figure 3A and 3B). Among controls, the degree of cohesin binding within +/− 1 kb of the TSSs is greatest for those genes that are actively transcribed and especially in those genes that are differentially expressed in the *NIPBL* mutant cell lines, whereas the silent nontranscribed genes have the same, or lower level, of enrichment as the background level (Figure 4A). In addition, cohesin binding is enriched at the 5′-UTRs only for actively transcribed genes, with no binding difference at exons, introns, or 3′-UTRs between the actively transcribed and silent genes (Figure 4B). Identification of overrepresented cohesin binding near promoters suggests that cohesin may regulate gene expression as a transcription factor. In spite of this, the majority of the expressed genes (78%) do not harbor any cohesin binding sites in their intragenic regions, indicating most of the genes in the human genome may be regulated by cohesin independent pathways or cohesin is involved in their expression regulation through an alterative mechanism. We further evaluated 13 genomic loci based on their gene expression alterations to validate their cohesin binding status by the more sensitive method of ChIP-quantitative PCR (qPCR). Out of these 13 loci, two regions are equally bound by cohesin in both healthy and CdLS cells, two regions are not bound by cohesin in either healthy or CdLS cells, and the remaining nine loci demonstrated significant loss of cohesin binding in CdLS cells as compared to control cells. The ChIP-qPCR results are consistent with cohesin binding alterations detected by ChIP array studies (Figure S2A and S2B, Table S11).

### Reduced Cohesin Binding Correlates with Transcription Dysregulation in CdLS

The total number of cohesin binding sites is reduced by 29.7% (9,530 versus 13,560) in the examined CdLS proband, but the total number of binding sites at TSSs is reduced by 43.4% (448 versus 792) in the same proband, suggesting that cohesin is more likely to be removed from TSSs (43.4% versus 29.7%, *p* = 5.9e−8) (Table 1). The 10,378 genes expressed in LCLs have been statistically ranked for their misexpression in CdLS probands as described above. In the controls, there exist 666 LCL expressed genes that have cohesin binding sites mapped to the +/− 1-kb vicinity of TSSs, and 107 of them are identified as differentially expressed in CdLS (FDR<0.05). In CdLS, only 376 such genes have cohesin sites around their TSSs (376 versus 666, reduced by 43.5%), only 53 of the 107 differentially expressed genes still maintain their TSS/cohesin association, whereas the rest have all lost their TSS cohesin binding sites (53 versus 107, reduced by 50.5%) in the proband (Table 2). At the TSSs, the number of cohesin sites on differentially expressed genes is significantly reduced in CdLS, whereas the reduction is moderate for the nondifferentially expressed genes, and only minimal for those silent nontranscribed genes (Figure 4A). The binding between cohesin and TSSs of expressed genes is highly correlated to the CdLS phenotype. In our identified panel of differentially expressed genes in CdLS, Fisher testing on the ChIP data shows that these genes tend to attract more cohesin to their TSSs in control cells under the healthy condition (*p* = 10e−4) than the neutral genes do, whereas under the diseased condition in CdLS cells, those genes tend to lose their capability to recruit cohesin and associate with cohesin at a similar level to the neutral genes and have lost their statistically significant difference (*p* = 0.1) (Table 2). This 2-kb region (+/− 1 kb surrounding the TSS) was further analyzed for the entire group of 10,378 genes expressed in LCLs that have been ranked for their differential expression in CdLS probands as described above. Cohesin enrichment was clearly identified in control cells for the top ranked genes and a dramatic decrease in binding is demonstrated in the CdLS cells, suggesting that the genes that harbor more cohesin sites around the promoter regions are more likely to be misexpressed in CdLS (Figure 4C). Moreover, this difference was even more remarkable if we narrowed the analyzed region to the +/− 100-bp central area surrounding TSSs (Figure S3).

**Table 1:** Cohesin associated to the +/− 1-kb vicinities of TSSs among three different groups of genes in control and CdLS LCLs.

Group	Expression Status in LCLs			Genes with Binding Sites Present at±1-kb Region Surrounding TSS	
	Control Cells	CdLS Cells (FDR<0.05)	Unique RefSeq Genes (15,162 in Total)	Control Cells (792 in Total)	CdLS Cells (448 in Total)
**A**	No	No	4,784	126	72
**B**	Yes	No (FDR>0.05)	9,199	559	323
**C**	Yes	Yes (FDR<0.05)	1,179	107	53

Group A is not transcribed in LCLs, groups B and C are transcribed in LCLs, whereas group C is differentially expressed in CdLS (FDR<0.05).

**Table 2:** Differentially expressed genes tend to lose their cohesin binding at TSSs in CdLS samples.

Binding Sites Present	Yes/No/Total	Number of Differentially Expressed Genes (FDR<0.05)		Total
		Yes	No	
±**1-kb Vicinity of TSSs in controls (Fisher**'**s test ** ***p*** ** = 0.0001, OR = 1.54 [1.23–1.92])**	**Yes**	107	559	666
	**No**	1,072	8,640	9,712
	**Total**	1,179	9,199	10,378
±**1-kb Vicinity of TSSs in CdLS (Fisher**'**s test ** ***p*** ** = 0.1, OR = 1.29 [0.94–1.75])**	**Yes**	53	323	376
	**No**	1,126	8,876	10,002
	**Total**	1,179	9,199	10,378

Cohesin preferentially binds to the TSSs among differentially expressed genes in healthy controls, *p* = 0.0001. Differentially expressed genes lose cohesin binding at their TSSs in CdLS. The frequency is reduced to the same level as the genes without differential expression, *p* = 0.1.

To summarize, in control LCLs cohesin preferentially binds to transcribed genes at the TSSs as compared to the silent nontranscribed genes. The binding sites are even more enriched for the differentially expressed genes. In CdLS, cells tend to lose cohesin binding globally, however the cohesin sites at TSSs are more likely to be lost, most notably for the differentially expressed genes where loss of cohesin binding at the TSSs results in a binding frequency approaching the background level. The preferential binding to promoter regions suggests cohesin may play a role as a transcription factor.

**Figure 5:**
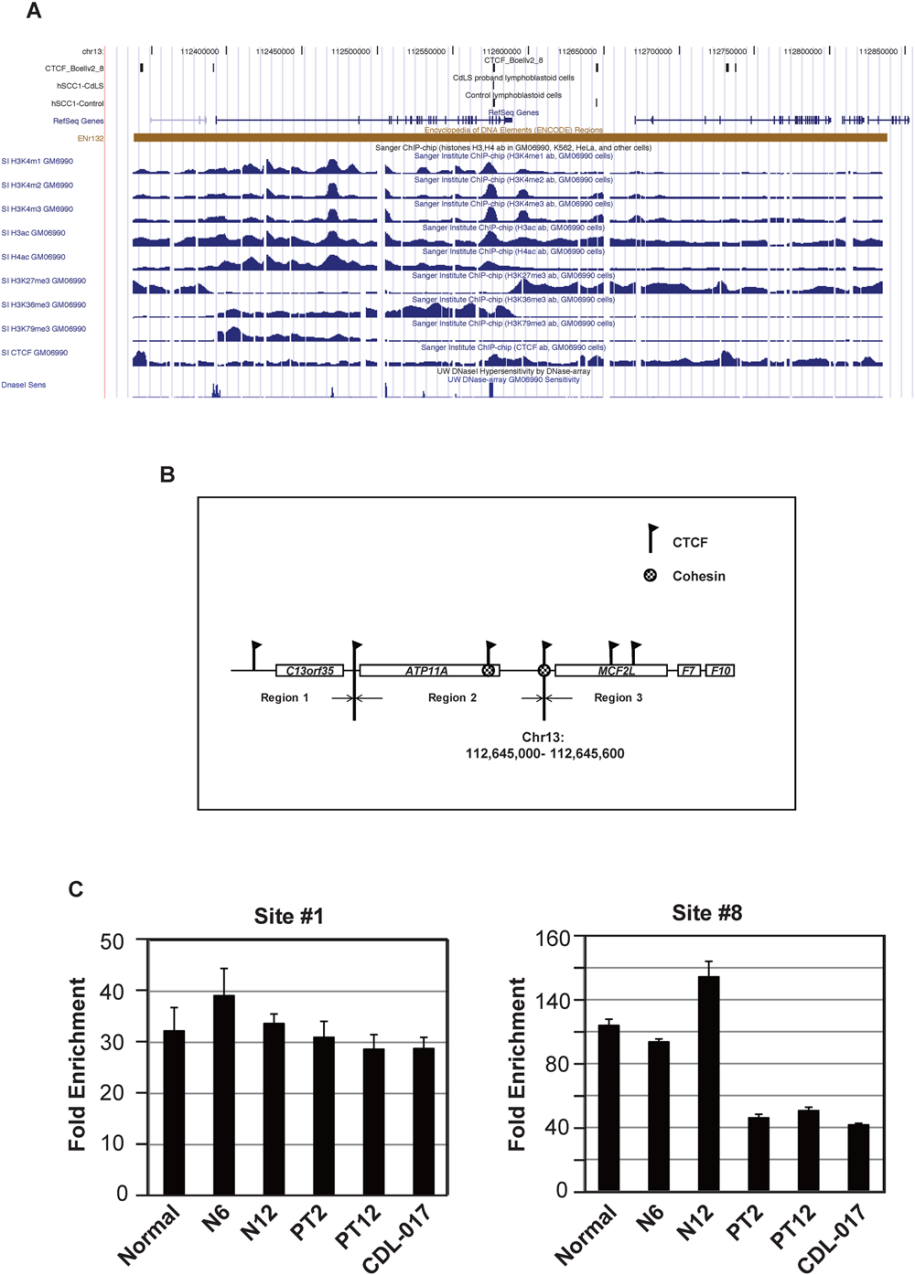
Cohesin and CTCF colocalize and separate the active chromatin region from the repressive chromatin region. The cohesin site at this position is lost in CdLS, thus the silencing epigenetic signal from region 3 is able to migrate into region 2, which harbors *ATP11A* and downregulates its transcription. (A) Screen shot of ENCODE ENr132 region from the UCSC genome browser is displaying histone methylation and acetylation status, CTCF binding sites, and DNaseI sensitivity sites on this region in GM06990 cells (from Sanger Institute and University of Washington databases, respectively). hSCC1-Control and hSCC1-CdLS are custom tracks. hSCC1-Control track indicates the results of whole genome cohesin binding analysis in LCLs from controls, whereas hSCC1-CdLS track indicates the results of whole genome cohesin binding analysis in LCLs from the CdLS proband; data on CTCF_Bcell2_8 track are adapted from Wendt et al. [10]. (B) Schematic of ENr132 locus as in (A). Five genes located in three regions are displayed. Two cohesin and six CTCF binding sites are shown. Cohesin and CTCF colocalize at Chromosome 13: 112,645,000–112,645,600, which separates region 2 from region 3. Cohesin binding at this position was lost in CdLS proband. (C) ChIP-qPCR validation in three different healthy controls “Normal,” “N6,” and “N12” and three additional CdLS probands “PT2,” “PT12,” and “CDL-017.” Cohesin bound to this locus was dramatically reduced among CdLS probands including a proband with an *SMC1A* mutation (CDL-017). Sites 1 and 2 are positive controls, site 8 spans Chromosome 13: 112,645,000–112,645,600.

Recently cohesin has been functionally linked to CTCF, an insulator capable of blocking enhancers or preventing the spread of epigenetic signals [15]. In our study, the ion transporter protein ATP11A is significantly downregulated in CdLS (FDR = 0.027), although the fold change is small (−1.24). *ATP11A* locates within ENCODE region ENr132 on Chromosome 13 with four other genes. Therefore, the ENCODE datasets obtained from GM6990, a similar EBV-transformed human B cell line (http://genome.ucsc.edu/, http://www.sanger.ac.uk/PostGenomics/encode/data-access.shtml), were able to be adapted for our analysis [21]. There are six CTCF and two cohesin binding sites in this area, both cohesin sites overlap with CTCF sites. In controls, this area can be split into three chromatin regions according to multiple histone modification makers (Figure 5A and 5B) [22]–[24], and cohesin and CTCF colocalize at the border. Region 1 harbors only one gene *C13orf35*, which is not expressed in LCLs. Region 3 harbors three genes, *MCF2L*, *F7*, and *F10*, which are all expressed comparably in LCLs from both controls and probands. The ENCODE study has shown that chromatin-silencing marker H3K27me3 is enriched in region 3, but open chromatin markers H3K4 me1/me2/me3, H3K36me3, and H3K79me3, and DNaseI sensitive sequences are underrepresented, indicating chromatin in this region is condensed and transcription repressed [22]–[24]. In region 2, on the other hand, H3K4 is highly methylated, H3 tails are vastly acetylated, and multiple DNaseI sensitive sites appeared; meanwhile, H3K27 methylation level is quite low indicating region 2 is an active open chromatin domain. *ATP11A* is the only gene located in region 2 and differentially expressed in CdLS. Of note, the cohesin binding site between regions 2 and 3 at Chromosome 13: 112,645,000–112,645,600 is lost in CdLS (Figure 5A and 5B). ChIP-qPCR was then performed using specific primers to amplify this binding locus in an expanded sample set including three healthy controls and three CdLS probands (Figure 5C). Two of the three probands, PT2 and PT12, have *NIPBL* truncating mutations with severely affected clinical features and have been included in the whole genome expression array studies as described above; the third proband CDL-017 has a mutation in the *SMC1A* gene and manifests a much milder phenotype (Tables S1 and S7). Cohesin binding site 1 (Chromosome 3: 79653256–79653385), which was not lost in CdLS according to our qualitative array analysis was therefore used as a positive binding control. By quantitative PCR assays, the enrichment of cohesin bound to site 1 was not found to be changed between controls and the probands, which is consistent with the array findings. However, cohesin binding was dramatically reduced, within Chromosome 13: 112,645,000–112,645,600 among CdLS probands including the individual with the *SMC1A* mutation (Figure 5C). Although cohesin binding was not completely lost in CdLS by ChIP-qPCR, the result is consistent with the missing binding peak seen in the qualitative ChIP array analysis. Although this dataset is limited, it suggests that cohesin possesses a function as an insulator/boundary protein, in addition, functional NIPBL is required for this process. With disruption in the *NIPBL* mutated or cohesin subunit *SMC1A* mutated human cells, the silent chromatin signals from region 3 appear to be able to cross the boundary and migrate into region 2 to inhibit *ATP11A* transcription. Cohesin and CTCF may function cooperatively at this locus owing to their colocalization. In addition, both CTCF binding sites remained intact in CdLS, which may explain why the downregulation of *ATP11A* was not dramatic (−1.24).

### Data Mining, Gene Ontology, Function, and Pathway Analyses

Ingenuity Pathways Analysis (IPA) (Ingenuity Systems, Inc., http://www.ingenuity.com) was used to analyze the identified differentially expressed genes. Out of the 339 genes with FDR<0.01, 150 genes are documented in cancer, neurological, hematological, skeletal and muscular, and dermatological diseases; 150 genes are identified as major players in embryonic and tissue development, hematological and immune system development and functions; in addition, 153 genes have well established cellular and molecular functions in cell death, cell proliferation, and cell cycle regulation. We have further analyzed the biological functions and canonical pathways mediated by the 23- and 10-gene classifiers and the three biomarkers as validated by target array (Table S12). Interestingly, more than 60% (15 out of 23) of the identifier genes harbor intragenic cohesin binding sites, which is much higher than the average genome level (22%). Moreover, some of these genes have completely lost their cohesin association in CdLS (Table S12). Both groups of classifier genes are tightly related to pathways of cell death, cellular development, and tissue morphology. 12 out of these 23 genes are involved in 47 known biological functions or disease conditions. Five of these 12 genes are also part of the 10-gene classifier, including two genes, *NFATC2* and *PAPSS2,* which are the identified biomarkers for CdLS. The 23-gene classifier could differentiate CdLS probands with *NIPBL* mutations suggesting the expression of these 23 genes are tightly controlled by NIPBL; whereas the 10-gene classifier is less powerful and only able to identify CdLS probands without the ability to differentiate subgroups of probands with different gene mutations, suggesting that these ten genes are related to terminal events during CdLS pathogenesis. Therefore, the common five genes, *PAPSS2*, *NFATC2*, *MAP3K5*, *LTB*, and *PHF16*, which are involved in multiple reported events by IPA and are shared by the two classifiers, might be involved in cellular functions that are universally affected in CdLS. Presumably mutations in *NIPBL*, *SMC1A*, or *SMC3*, or as of yet unidentified CdLS causing gene mutations, will all result in alterations of the related biological functions controlled by these five genes. On the other hand, the seven unique genes with functional roles that were excluded from the 10-gene classifier, *KIFAP3*, *AIM1*, *BBS9 (PTHB1)*, *TSPAN12*, *TRERF*, *ARHGAP24*, and *ID3*, probably represent cellular functions affected more specifically by *NIPBL* mutations. Four genes, *PAPSS2*, *NFATC2*, *MAP3K5*, *ADCY1*, were identified to be involved in 32 canonical pathways by IPA; they also regulate multiple biological functions as mentioned above. *ADCY1* is the single gene out of the above four genes that exists in the 23-gene classifier but is excluded from the 10-gene classifier; thus the specific canonical pathways regulated by *ADCY1* (i.e. B cell receptor signaling, RAR activation, sulfur metabolism, and endoplasmic reticulum stress pathways), could largely depend on normal functions of NIPBL. Two out of the three biomarkers, *NFATC2* and *PAPSS2,* are reported to be involved in multiple biological functions and canonical pathways by IPA analysis. The third biomarker ZNF608 is a novel protein with very minimal known functions. However, the zinc finger protein family members are known to be the major players in many molecular and cellular pathways.

One of the biomarkers, NFATC2, is involved in multiple signaling pathways during development and affecting skeletal myogenesis, chondrogenesis, axon growth, and guidance [25],[26]. Two NFATC negative regulators both locate to the Down syndrome critical region of human Chromosome 21, *Nfatc2* ^−/−^ and *Nfatc4* ^−/−^ double-knockout mice have physical and cognitive features resembling human Down syndrome [27]. Dysregulation of NFATC2 in the postnatal nervous system may contribute to mental deficiency in CdLS. Another biomarker, PAPSS2, plays a pivotal role in the biosynthesis of sulfate donors for sulfotransferase reactions. Its activity is important for normal skeletal development; recessive mutations on *PASS2* cause the genetic disorder spondyloepimetaphyseal dysplasia (SEMD), Pakistani type and degenerative osteoarthritis [28]. *Papss2* ^−/−^ knockout mice have shortened limbs, reduced axial skeletal length, and complex facial features. Its transcripts were also present in the heart and brain in mouse embryos [29].

## Discussion

Cohesin consists of four major proteins SMC1A, SMC3, SCC1, and SCC3. NIPBL plays a role in shuttling cohesin onto and off the chromatin, although the exact mechanism of its action is poorly understood. All proteins in this pathway are evolutionally conserved from yeast to human [30]. Cell-cycle related sister chromatid cohesion, DNA repair, and homologous rearrangement are well established roles for the cohesin apparatus. A role for cohesin in regulating gene expression has also been proposed and appears to be more sensitive to subtle dosage alterations of the cohesin apparatus and its regulators than its canonical function in sister chromatid cohesion [31]. In both yeast and *Xenopus*, the loading of cohesin onto chromatin in G_1_ phase is functionally separable from the establishment of sister chromatid cohesion at S phase [32],[33]. In *Drosophila,* Nipped-B mediates interactions between the promoter and remote enhancers for *cut* and *Ultrabithorax*; heterozygous *Nipped-B* mutants diminish *cut* expression in the emergent wing margin without showing cohesion defects indicating sister chromatid cohesion is independent from cohesin regulated gene expression [34]. In mice, *Pds5b* mutants have multiple CdLS-like defects without flawed sister chromatid cohesion [35].

In humans, CdLS is caused by heterozygous loss-of-function mutations in the *NIPBL* ortholog of *Nipped-B* and, in a smaller percent of cases, by mutations in the *SMC1A* or *SMC3* cohesin subunit genes [17],[18],[36],[37]. Given the constellation of developmental abnormalities present in individuals with CdLS, with only a subset showing minor cohesion defects [20],[38], it is likely that the alterations of cohesin regulation and structure seen in these individuals result in gene expression dysregulation. We chose to use an easily accessible but a seemingly developmentally irrelevant tissue, LCLs, for these studies. We hypothesized that congenital genetic disorders might arise, in part, through dysregulation of expression of specific genes and that expression differences between affected and unaffected individuals might be present in tissues other than disease presenting tissues. These cells also provide an invaluable resource of naturally occurring mutant cohesin proteins (both structural and regulatory components of cohesin) that can be used to study the cellular processes regulated by this complex and specifically the impact on regulation of gene expression. Surprisingly these cells may also provide valuable insight into human developmental processes as well.

We have identified specific gene expression profiles for CdLS that are capable of classifying probands and tightly correlate with disease severity. Cohesin preferentially binds to promoter regions of the actively expressed genes suggesting a role as a general transcription factor. These binding sites are significantly reduced in *NIPBL* mutant CdLS samples. This result is likely due to NIPBL's direct role in cohesin loading on chromatin, which in turn affects transcriptional regulation at specific loci and would contribute to the CdLS pathogenesis. Out of the 339 dysregulated genes with FDR<0.01, 202 were upregulated (59.6%) and 137 were downregulated (40.4%), more genes were reactivated than inhibited with mutations in *NIPBL* (59.6% versus 40.4%, *p* = 3.44e−17) suggesting that NIPBL and cohesin can result in both negative (as transcriptional repression) and positive (as transcriptional activation) effects on expression. A similar percentage of upregulated and downregulated genes was also observed among the 1,501 dysregulated genes with FDR<0.05. Moreover, 71 of the above 339 genes (20.9%) and 207 of the 1501 genes (13.8%) have fold changes larger than 1.5, whereas the highest fold changes are −4.2 and +4.6, respectively. Although the majority of expression levels seemed only mildly perturbed, developmental deficits in CdLS are likely due to a cumulative change in multiple genes. Another reason for less remarkable expression differences could be the LCL tissue type used for this study, with bigger fold changes in more genes possibly present in more directly affected tissues of, e.g., brain or limb, and at specific times during embryonic development. However, it is also possible that the transcriptional dysregulation may be directly mediated by *NIPBL* through a yet uncharacterized mechanism and the reduced cohesin binding may be a secondary effect. In our study, a 30% reduction in NIPBL message was able to trigger a 29.7% (9,530 versus 13,560) reduction in cohesin binding sites in CdLS probands and further affects the transcription of specific genes.

The central components for sister chromatid cohesion, *RAD21 (SCC1)*, *SMC1A*, *SMC3*, *STAG2*, *ESCO1*, *ESCO2*, and *PDS5A* (also known as *SCC-112*), are all expressed similarly between controls and CdLS probands with *NIPBL* mutations. However, *STAG1*, *PDS5B* (also known as *APRIN*), *MAU2L (KIA0892)*, as well as several other genes with functions related to sister chromatid cohesion were significantly dysregulated in *NIPBL* mutant CdLS probands (FDR<0.05) (Table S13), suggesting that the cohesin pathway itself is affected by mutant NIPBL. *MAU2 (KIAA0892)* is the putative human homolog of *scc4* in *Caenorhabditis elegans* [31],[39]. It forms an essential loading complex with NIPBL that regulates cohesin-chromatin association, sister-chromatid pairing, and mitotic checkpoints in HeLa cells. Physical association between NIPBL and MAU2 is indispensable for their stability, as depletion of either of the two proteins would subsequently diminish the cellular level of the other one [39]. In our study, decreased NIBPL transcription (−1.33, FDR = 0) was able to upregulate the transcription of MAU-2 (+1.11, FDR = 0.026), suggesting a functional compensation may exist for cohesin loading in CdLS. A cohesin-independent mechanism has also been suggested to exist. Condensin complexes [40], origin recognition complexes (ORCs) [41], centromere complexes [42], and DNA catenation [43] have each been reported to play a role in mediating cohesin-independent sister chromatid cohesion. Genes involved in these functions are also found to have dysregulated expression in *NIPBL* mutant individuals (Table S13). This finding indicates a subset of genes regulated by NIPBL are tightly involved in sister chromosome segregation events, but expression alteration may be required to pass a certain threshold in order to induce visible cohesion defects. This observation could explain why cell lines derived from CdLS probands did not demonstrate significant sister chromatid pairing problems. In contrast to CdLS, cohesion defects have been reported in three human developmental disorders: RBS (OMIM 268300) [19], Rothmund–Thomson syndrome (RTS, OMIM 268400) [44], and α-Thalassemia/mental retardation syndrome, X-linked (ATRX, OMIM 301040) [45]. Interestingly, although the expression of the RBS disease causative gene *ESCO2* was not dysregulated in CdLS cell lines, the other two disease genes, *ATRX* and *RECQL4*, both demonstrated dysregulation in *NIPBL* mutant cell lines (Table S13).

Several cohesin targets have been identified. Steroid hormone ecdysone receptor (*EcR*), which is the *Drosophila* homolog of human *NR1H3*, was suggested to be regulated by Smc1, and *Runx3* was identified as a direct target of Rad21 in zebrafish [46],[47]. The fact that both of these genes were also significantly dysregulated (FDR<0.05) in CdLS probands with *NIBPL* mutations indicates that NIPBL may first affect cohesin proteins and subsequently dysregulate cohesin targets. Surprisingly, we did not find that cohesin directly binds to these two genes in the cell line studied, which raises the possibility that cohesin may regulate their expression over long distances. When comparing ChIP-on-chip results for Nipped-B and/or SMC1A binding sites in three different *Drosophila* cell types [48], homologs of 20 differentially expressed human genes in CdLS probands (FDR<0.05) were also found to be bound by NIPBL and cohesin (unpublished data). Eight of these 20 genes are also bound by cohesin in humans suggesting they may be cohesin targets in both *Drosophila* and humans. It also suggests that cohesin mediated transcription is a conserved biological event. Moreover, most of the binding sites were lost in CdLS cells indicating dysregulated gene expression correlates with loss of cohesin binding. Among the eight genes, *KMO*, *ELL2*, and *ARHGAP17* have cohesin binding at TSSs; *ROBO1*, *UBE2H*, *MED13L*, *RASA3*, and *PDPK1* had cohesin binding within intronic regions. One of these genes, *ROBO1* (homolog of *lea* in *Drosophila*), is of particular interest as it was found to have a fold change of 4.6, which is the largest among all the genes on the array. *ROBO1* has been associated with dyslexia, a neurocognitive disorder of language and graphic processing that could be due to the abnormal migration and maturation of neurons during early development.

We have identified groups of 23, 10, and 3 genes as CdLS classifiers or biomarkers that are capable of differentiating CdLS from non-CdLS samples. The expression levels of these genes also correlate to the phenotypic severity of this disorder, although it is not clear at this time how the dysregulation of these particular genes might contribute to the phenotypes. More than 60% of the identifier genes harbor intragenic cohesin binding sites with some of them lost in CdLS proband. The obvious overrepresentation of genes carrying intragenic cohesin binding sites among the CdLS classifier genes further suggests that expression of the dysregulated genes is tightly related to the availability of cohesin binding. Overall, the majority of genes do not carry known cohesin binding sites, indicating that cohesin may play an upstream role in regulating human genes, or cohesin may enact regulation on some of the genes through distal *cis*- or *trans*-regulatory elements. The potential role for cohesin independent NIPBL regulation can not be excluded.

Cohesin has recently been found to be physically and functionally associated with the vertebrate insulator protein CTCF. In our study cohesin binds to only ∼20% of genes intragenically. This distribution does not change much between expressed genes and silent genes, and between differentially expressed genes in CdLS and disease neutral genes. Cohesin could be involved in gene regulation, like CTCF, by either binding to promoter elements and having a direct influence on the transcriptional machinery or by binding to intergenic *cis*-elements such as insulators to control gene expression from remote distances [49]. In our study, we have detected a potential boundary effect of cohesin at the *ATP11A* gene locus that suggests, for the first time in humans, that cohesin may bind to insulators and regulate transcription. Reduced cohesin binding at this locus was further validated in three additional CdLS probands by the more sensitive ChIP-qPCR including probands with either *NIPBL* mutations or cohesin subunit *SMC1A* mutation. However, cohesin does not exactly mimic the function of CTCF, at least in LCLs. Some CTCF target genes, such as *PIM-1* [50] and *APP* [51], although expressed in LCLs, are neither dysregulated in CdLS nor do they lose cohesin binding at their regulatory regions. On the other hand, the CTCF target gene, *BRCA1* [52], was downregulated in CdLS (−1.2, FDR = 0.017) but without losing cohesin binding sites. Additional quantitative analysis or ChIP-qPCR to study more genomic loci will delineate a clearer picture of cohesin and CTCF effects on transcriptional regulation. The role cohesin plays in imprinting and X inactivation remains unclear [53].

**Figure 6:**
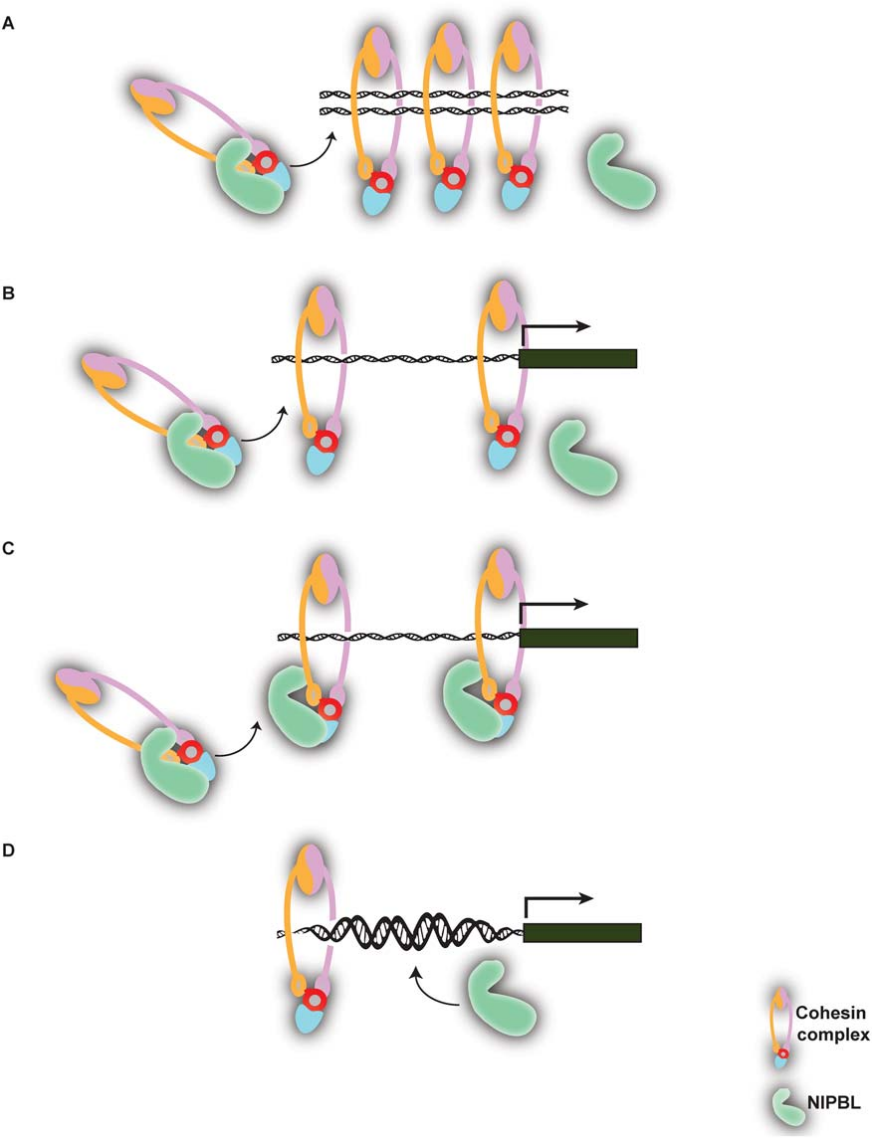
Proposed working models for cohesin and NIPBL. (A) Cohesin's canonical role in regulating sister chromatid cohesion with NIPBL acting to facilitate the loading and unloading of the cohesin complex onto the chromosomes. It is not known if NIPBL directly interacts with chromatin. This model was described by Haering et al. [54]. (B) Cohesin loading model: NIPBL loads cohesin onto chromatin at the promoters or *cis*-regulatory elements after which cohesin regulates transcription without the direct involvement of NIPBL. (C) Cohesin and NIPBL collaborative model: Cohesin and NIPBL form a protein complex that binds to promoters or *cis*-regulatory elements. The functional integrity of this complex is required for transcriptional regulation of target genes. (D) NIPBL chromatin remodeling model: NIPBL may affect the accessibility for cohesin, e.g., by changing chromatin structures, to bind to chromatin elements through yet unknown pathways. Transcriptional regulation through cohesin is secondarily affected.

In summary, we have undertaken a genome-wide approach to study gene expression and cohesin binding in *NIPBL* mutant human samples. On the basis of our data and previously reported studies, we propose that NIPBL may be involved in modulating cohesin function through various mechanisms. Besides its canonical role in regulating sister chromatid segregation proposed by Haering et al. [54] (Figure 6A), cohesin may also regulate transcription (1) as an insulator protein by acting alone or with CTCF, or (2) as a transcription factor by binding to promoter elements. While regulating transcription, NIPBL may also serve as a cohesin shuttle to chromatin that leads to decreased cohesin binding when NIPBL is mutated. Data from this study are quite consistent with this role. Whether this loading mechanism either partially overlaps with, or is completely independent from NIPBL-mediated sister chromatid cohesion remains unknown (Figure 6B). NIPBL and cohesin may very well form one protein complex binding to regulatory elements of target genes, with NIPBL mutations affecting the regulatory capacity of this complex (Figure 6C). The colocalization of NIPBL and cohesin seen in *Drosophila* studies could be consistent with this model [9]. A third possibility is that NIPBL is able to maintain an accessible chromatin structure for cohesin binding whereas defective NIPBL leads to reduced accessibility for cohesin at specific chromosomal loci (Figure 6D).

## Materials and Methods

### Ethics Statement

This study was conducted according to the principles expressed in the Declaration of Helsinki. The study was approved by the Institutional Review Board of The Children's Hospital of Philadelphia and Misakaenosono Mutsumi Developmental, Medical, and Welfare Center. All patients provided written informed consent for the collection of samples and subsequent analysis.

### Sample Collection

All participants were evaluated by one or more experienced clinicians. Gene mutations were confirmed by sequencing, and most of the cases have been previously reported by our laboratories [17],[55],[56].

### Cell Lines and Culture Condition

LCLs were grown uniformly in RPMI 1640 with 20% fetal bovine serum (FBS), 100 U penicillin/ml, 100 µg streptomycin/ml sulfate, and 1% L-glutamine. To identify differentially expressed genes between CdLS probands and controls, age and gender matched samples from 16 normal controls of European descent and 17 clinically severely affected probands of European descent with *NIPBL* protein-truncating mutations (nonsense or frameshift) were chosen as the training set for the discriminate analysis. To validate the expression pattern obtained from the training set, six samples including one healthy control, one Egyptian CdLS proband, two Roberts syndrome probands, and two Alagille probands were used as the testing set. All 39 cell lines were grown anonymously and the processing of these 39 cell lines were randomized by genotypes to eliminate batch effects that may contribute to genotype-specific gene expression. Samples are listed in Table S1A and S1B with detailed description. For custom array analysis, detailed information of these samples is listed in Table S7. Out of these 101 samples of European descent, the training set included 17 healthy controls and 14 severely affected CdLS probands with *NIPBL* protein-truncating mutations. All 31 samples were also used for the training in Affymatrix array analysis. For the testing set, all new samples were selected, which included four healthy controls, six severely affected probands, 13 moderately affected probands (nine have *NIPBL* mutations and four do not have an identifiable mutation), and 34 mildly affected probands (26 have *NIPBL* mutations and eight do not have an identifiable mutation). We have also included nine CdLS probands with *SMC1A* mutations, as well as four samples with different genetic diagnoses (two AGS, one Roberts syndrome, and one unknown multisystem genetic disorder). As above, samples were processed anonymously and randomly.

5×10^6^ exponentially growing cells were seeded in 15 ml media in a 75-ml Falcon flask, and fed exactly after 24 h. After an additional 24 h on day 3, 8 ml of the media was removed and cells were pelleted by centrifuge and RNA extraction was performed immediately.

### RNA Isolation and Affymatrix Expression Array Hybridization

Total RNA from each sample was extracted with the RNeasy Mini-kit (Qiagen), synthesis of double-stranded cDNA was performed using SuperScript Double-Stranded cDNA Synthesis kit (Invitrogen), and cleaned up with GeneChip Sample Cleanup module (Affymetrix). The resulting products were then used to synthesize biotin-labeled cRNA with Enzo Bioarray High Yield RNA Transcript Labeling kit (Enzo Life Sciences) and further fragmented to 35–200-bp oligos. All procedures were done according to manufacturer's instructions. 30 µl fragmented cRNA at the concentration of 500 ng/µl was sent for hybridization in the microarray facility at The Children's Hospital of Philadelphia. Microarray hybridizations were performed by using HG-U133 plus 2.0 GeneChips (Affymetrix). The HG-U133 plus 2.0 contains ∼54,000 25-mer probe sets that covers approximately 47,000 transcripts and variants out of which 38,500 are well-characterized human genes. After hybridization and washes, arrays were scanned and analyzed both for genes that were present and for expression level using Microarray Analysis suite (MAS) 5.0 using default settings according to manufacturer's instructions.

### Custom-Built Target Array Hybridization

The same RNA isolation process was performed as above. 32 genes were selected by clustering-based feature selection and 59 probes were designed (Table S8). Probe designing, RNA labeling, and hybridization were conducted using the Ziplex workstation (Xceed Molecular, http://www.xceedmolecular.com/). In brief, concentrations of the isolated RNA were determined by measuring the absorbance at 260 nm. All total RNA samples were of acceptable purity (ratio of the absorbance at 260 nm to 280 nm of 1.75 or greater). The integrity of the total RNA was determined to be acceptable for all samples (RNA Integrity Numbers measured with the Agilent 2100 Bioanalyzer RNA 6000 Nano assay were greater than 9.0). A custom Ziplex TipChip microarray containing oligonucleotide probes of between 35 and 50 bp for 32 genes was used to profile differences in gene expression between the LCL samples. Total RNA (500 ng) from 108 independent samples was amplified and biotin labeled with the Illumina Totalprep RNA amplification kit (Ambion). The concentrations of the labeled aRNAs were determined by measuring the absorbance at 260 nm, and 3 µg was hybridized on the custom TipChip with the Ziplex Automated Workstation protocol (Xceed Molecular). After hybridization, the Ziplex Automated workstation software automatically quantified spot intensities and reported background subtracted expression values. The Ziplex software automatically evaluated attributes of each spot to identify spots that did not conform to quality control criteria and reported the mean value of the duplicate spots of each probe that passed quality control.

### ChIP Microarray Analysis

Two healthy controls and one severely affected CdLS proband with an *NIPBL* protein-truncating mutation (G5483A) were used. Cells were crosslinked with 1% formadehyde at 70%–80% confluency for 10 min, chromatin was then prepared after quenching with 125 mM glycine and ChIP was performed as described [57] using anti-hRAD21 polyclonal antibodies (Abcam, ab992). In brief, lysates from crosslinked cells were incubated with the antibodies and preabsorbed protein A Affiprep beads (Bio-Rad) for 14 h at 4°C and for 2 h at 4°C, respectively. After washing, the beads were incubated in the elution buffer (50 mM Tris, 10 mM EDTA, 1% SDS) for 20 min at 65°C. The elutes were treated with proteinase K for 1 h at 37°C and followed by 65°C overnight incubation for crosslink reversal. The samples were then treated with RNase and phenol-chloroform purified for one time, and further purified using PCR purification kit (Qiagen) with 80 µl water used for the final elution.

The eluted chromatin was amplified and labeled with biotin then hybridized to high-density oligonucleotide tiling arrays (Human tilling 2.0R array, Affymetrix) as described [58]. A sample of DNA prepared from whole cell extract (WCE) was prepared in the same way. ChIP and WCE samples were hybridized on arrays according to the manufacturer's instructions, two technique replicates were used for each sample. After scanning and data extraction, enrichment values (ChIP/WCE) were calculated by using the MAT algorithm [59]. MAT is designed for high-density oligonucleotide tiling-array analyses in higher eukaryotes that could reduce probe-specificity biases because of genome complexity or high GC content. The resulting MAT scores are proportional to the logarithm transformed value of the fold-enrichment of the ChIP-chip samples [59]. We mapped MAT scores to positions in human genome assembly Hg 18 (NCBI Build 36). Bandwidth, MaxGap, and MinProbe parameters were set to 250, 1,000, and 12, respectively. The cutoff threshold of *p*-values was set to 1×10^−6^, which was equivalent to MAT scores higher than 4.85. FDRs were also calculated with every experiment less than 1% (Figure S4A and S4B). BED files were created, data were visualized in the Integrated Genome Browser (IGB) (http://www.affymetrix.com/support/developer/tools/download_igb.affx) and University of California Santa Cruz (UCSC) genome browser custom track (http://genome.ucsc.edu/).

### ChIP-qPCR

ChIP was performed as described above using hRAD21 and control antibodies. ChIP-qPCR analysis was performed as previously described [10]. ChIP samples (2 µl) were used for one 25-µl PCR reaction. Analyses by qPCR were performed using a Platinum SYBR Green qPCR SuperMix UDG (Invitrogen) on an ABI 7500 cycler. The results were presented as fold-enrichment over control ChIP.

### Statistical Analysis

Gene expression microarray data were processed by DNA-Chip Analyzer (dChip) (http://www.dchip.org) using PM-only background subtraction and invariant set normalization. Differential gene expression between controls and CdLS probands was ranked by the ratio of between- and within-group variance (*F* statistic). During nearest centroid classification, distance of testing samples to training group centroids was measured as their Pearson's correlation coefficient. Statistical analyses were performed within R software environment (http://www.r-project.org). PCA and heatmap plots were generated by Spotfire DecisionSite version 9.1.1 (Spotfire, Inc.). More details about data analysis are provided in Text S1.

### Accession Numbers

Genomic sequences reported in this manuscript have been submitted to NCBI GEO (http://www.ncbi.nlm.nih.gov/geo): gene expression data are under accession number GSE 12408 and ChIP-chip data are under accession number GSE 12603.

## Supporting Information

Figure S1 Expression level of three genes (*NFATC2*, *PAPSS2*, and *ZNP608*) for controls and probands. (A) 17 healthy controls and 14 severely affected CdLS probands with *NIPBL* protein truncating mutations, and (B) 101-sample cohort used for target array analysis including the same individuals as in (A). Three axes represent expression of the three genes, blue dots represent controls, including healthy participants and individuals with other genetic diagnoses; red dots represent CdLS probands.
(0.26 MB PDF)

Figure S2 ChIP-qPCR validation of 13 cohesin binding sites identified by ChIP array. RAD21 ChIP samples were obtained from the CdLS proband and the control in the ChIP array studies and were analyzed by qPCR for the presence of 13 different cohesin binding sites with site-specific primers (mean of *n* = 3; error bars +/− standard deviation [SD]) (see Table S11 for genomic addresses of the 13 sites and primer sequences). The results were presented as fold-enrichment over control ChIP (nonantibody). (A) The presence of cohesin binding at 13 examined genomic sites, sites 1 and 2 were bound equally by cohesin in both probands and control in the array studies and served as positive controls here; sites 3 and 4 did not demonstrate cohesin binding in either proband or control in the array studies and served as negative controls here; sites 5–13 are nine genomic sites where cohesin binding was lost in the CdLS cells by qualitative analysis in the array studies. Quantitative PCR has revealed the amount of cohesin bound to these sites is significantly reduced at all of the examined loci. (B) Quantitative analysis of average amount of cohesin bound to the nine examined sites revealed at least half of cohesin binding is lost in the CdLS cells.
(0.36 MB PDF)

Figure S3 Cohesin binding within +/− 100 bp around TSSs is enriched in differentially expressed genes. The 10,378 unique genes expressed in LCLs are ranked by their *F* scores. The reference enrichment is the overall percentage of genes having cohesin binding within 200 bp (+/− 100 bp) around TSSs. The relative enrichment is calculated as the value of cohesin binding enrichment in top-ranked genes over the reference enrichment. The relative enrichment point is calculated for the total number of genes prior to the point on the *x*-axis. The numbers on *x*-axis denote the number of top-ranked genes. The curves are smoothed by the LOWESS algorithm.
(0.23 MB PDF)

Figure S4 FDRs of genome-wide ChIP microarrays of (A) controls and (B) CdLS proband. The *x*-axis denotes the *p*-values and the *y*-axis denotes the average FDR percentage for each experiment. Note that the FDR is less than 1% at the threshold *p*-value = 10^−6^ adopted for the analyses performed in this study.
(0.33 MB PDF)

Table S1 39 training and testing samples were used for the whole genome expression array analyses. (A) 16 LCL samples from severely affected CdLS probands with identified protein-truncating mutations of *NIPBL* were used for the training set. (B) 17 LCL samples from healthy controls were also included in the training set for expression array analyses. An additional six samples were used as a testing set.
(0.23 MB PDF)

Table S2 1,915 probe sets representing 1,501 unique genes (FDR<0.05) are differentially expressed in CdLS.
(0.35 MB PDF)

Table S3 339 nonredundant genes represented by 420 probe sets (FDR<0.01) are differentially expressed in CdLS.
(0.24 MB PDF)

Table S4 Evaluation of Leave-One-Out cross-validation for the 33 samples in the training set. Two healthy controls and one proband were misclassified.
(0.22 MB PDF)

Table S5 Five functional independent gene clusters identified among the 339 genes (FDR<0.01) using GSEA online program and R code.
(0.24 MB PDF)

Table S6 32 genes chosen by clustering-based feature selection for custom array analysis.
(0.23 MB PDF)

Table S7 Cohort of 101 individuals of European descent selected for custom array validation. Clinical evaluation and gene mutations of this cohort are listed.
(0.27 MB PDF)

Table S8 56 probes designed for the 32 selected genes for the custom array.
(0.22 MB PDF)

Table S9 Step wise method to select the 23- and ten-gene classifiers and the three-gene biomarkers.
(0.23 MB PDF)

Table S10 Intragenic cohesin binding in mapped human RefSeq genes. The total number of mapped human RefSeq transcripts is 15,162, whereas 4,784 genes are not transcribed in LCLs (group A); 9,199 genes are transcribed but not differentially expressed in CdLS (group B); and 1,179 genes are both transcribed and differentially expressed in CdLS (group C). (A) Cohesin binding is reduced in group A genes (18.9% of 4,784 genes) but increased in group C genes (27.0% of 1,179 genes) as compared to all the mapped transcripts (22.0% of 15,162 genes). Group B genes demonstrate little change (22.9% of 9,199 genes) as compared to all mapped transcripts. In CdLS, the number of genes bound by cohesin in all the groups is significantly reduced (all transcripts, 22.0% → 16.0%; group A genes, 18.9% → 13.8%; group B genes, 22.9% → 16.8%; group C genes, 27.0% → 18.5%). (B) In both control and CdLS, when compared to the number of genes bound by cohesin in all the mapped transcripts (22.0% in control and 16.0% in CdLS), group A has a significantly reduced percentage of genes bound by cohesin (*p*≤7.2e−6 in control and *p*≤0.000187 in CdLS), whereas group C has a significantly increased percentage of genes bound by cohesin (*p*≤7.44e−5 in control and *p*≤0.0249 in CdLS), and group B does not demonstrate a statistically significant change in cohesin binding (*p*≤0.0864 in control and *p*≤0.0836 in CdLS). *p, binomial proportions comparing intragenic cohesin binding between control and CdLS; **p, binomial proportions comparing intragenic cohesin binding between individual group (A, B, or C) and all the mapped transcripts.
(0.27 MB PDF)

Table S11 Specific primer pairs used for ChIP-qPCR validation. Primer pair 1 and 2 amplify regions that are bound by cohesin equally in healthy and CdLS cells, and served here as positive controls. Primer pair 3 and 4 amplify regions that are not bound by cohesin in either healthy or CdLS cells, and served as negative controls. Primer pair 5 to 13 amplify regions that were identified as having lost cohesin binding in CdLS cells by the qualitative ChIP array studies.
(0.22 MB PDF)

Table S12 Intragenic cohesin binding in the classifier genes and the gene ontology analysis. The appearance of a cohesin binding site is described as “**+**,” binding in both CdLS proband and control cells is shown. The involvements of multiple bio-functions and canonical pathways of each gene are also listed based on the IPA analysis. The 10-gene classifier and the three biomarkers are part of the 23-gene classifier.
(0.27 MB PDF)

Table S13 Dysregulated genes (FDR<0.05) identified in CdLS probands with *NIPBL* mutations that are functionally related to cohesion pathways. Genes that have FDR between 0.05 and 0.1 are highlighted in red.
(0.23 MB PDF)

Text S1 Supporting methods and statistical analysis.
(1.31 MB PDF)

## Abbreviations

AGS: Alagille syndrome
CdLS: Cornelia de Lange syndrome
ChIP: chromatin immunoprecipitation
FDR: false discovery rate
IPA: Ingenuity Pathways Analysis
LCL: lymphoblastoid cell line
MAT: model-based analysis of tiling-arrays algorithm
*NIPBL*: Nipped-B-Like
OMIM: Online Mendelian Inheritance in Man
qPCR: quantitative PCR
RBS: Roberts-SC phocomelia syndrome
TSS: transcription start site
TTS: transcription termination site
