# Supplementary figures and images for: Transcriptional Dysregulation in NIPBL and Cohesin Mutant Human Cells
Source: PLoS Biol. 2009 May 26;7(5):e1000119. doi: 10.1371/journal.pbio.1000119 (PMC2680332; doi:10.1371/journal.pbio.1000119)

Figure S1.

A

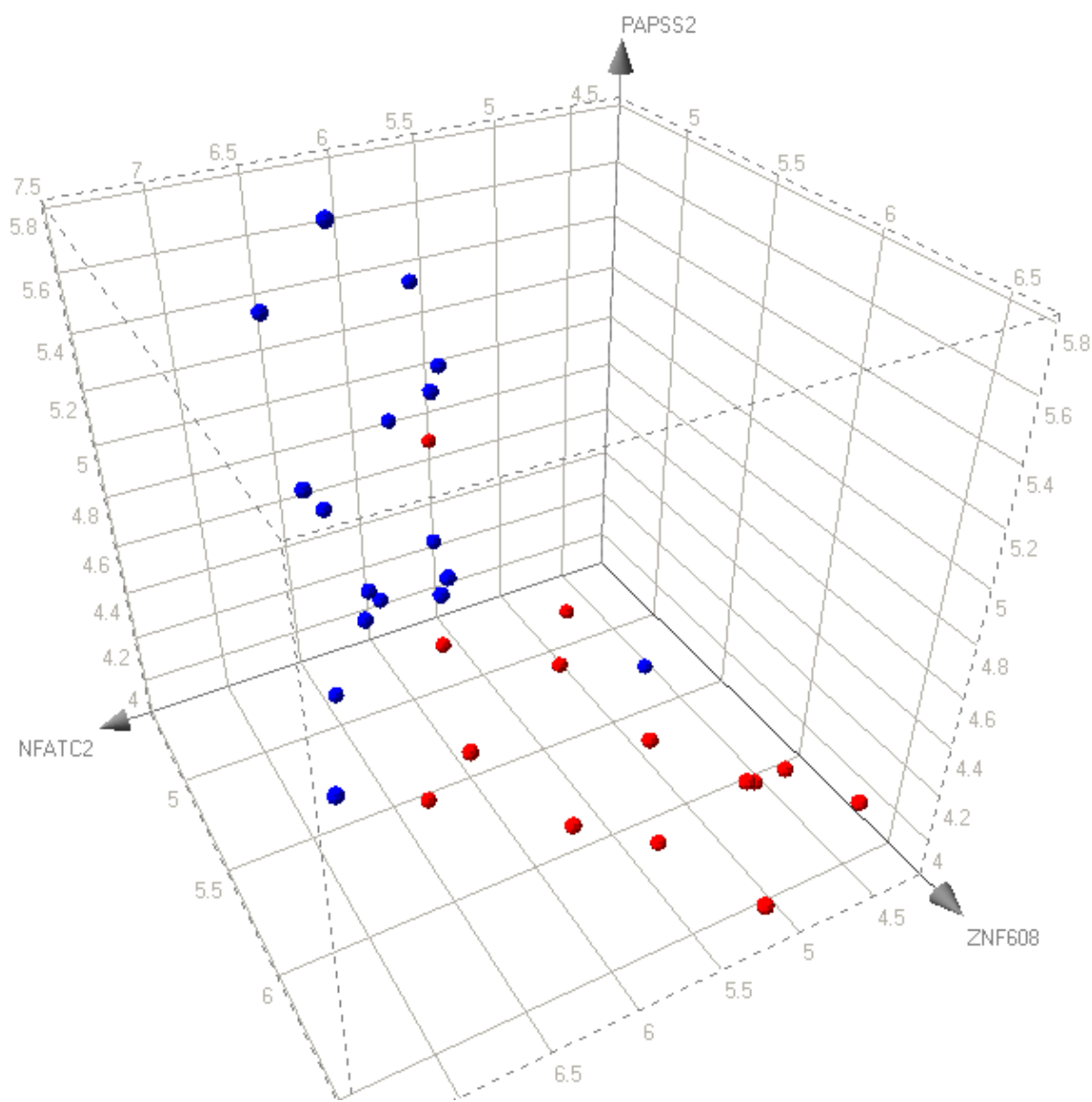

**B**

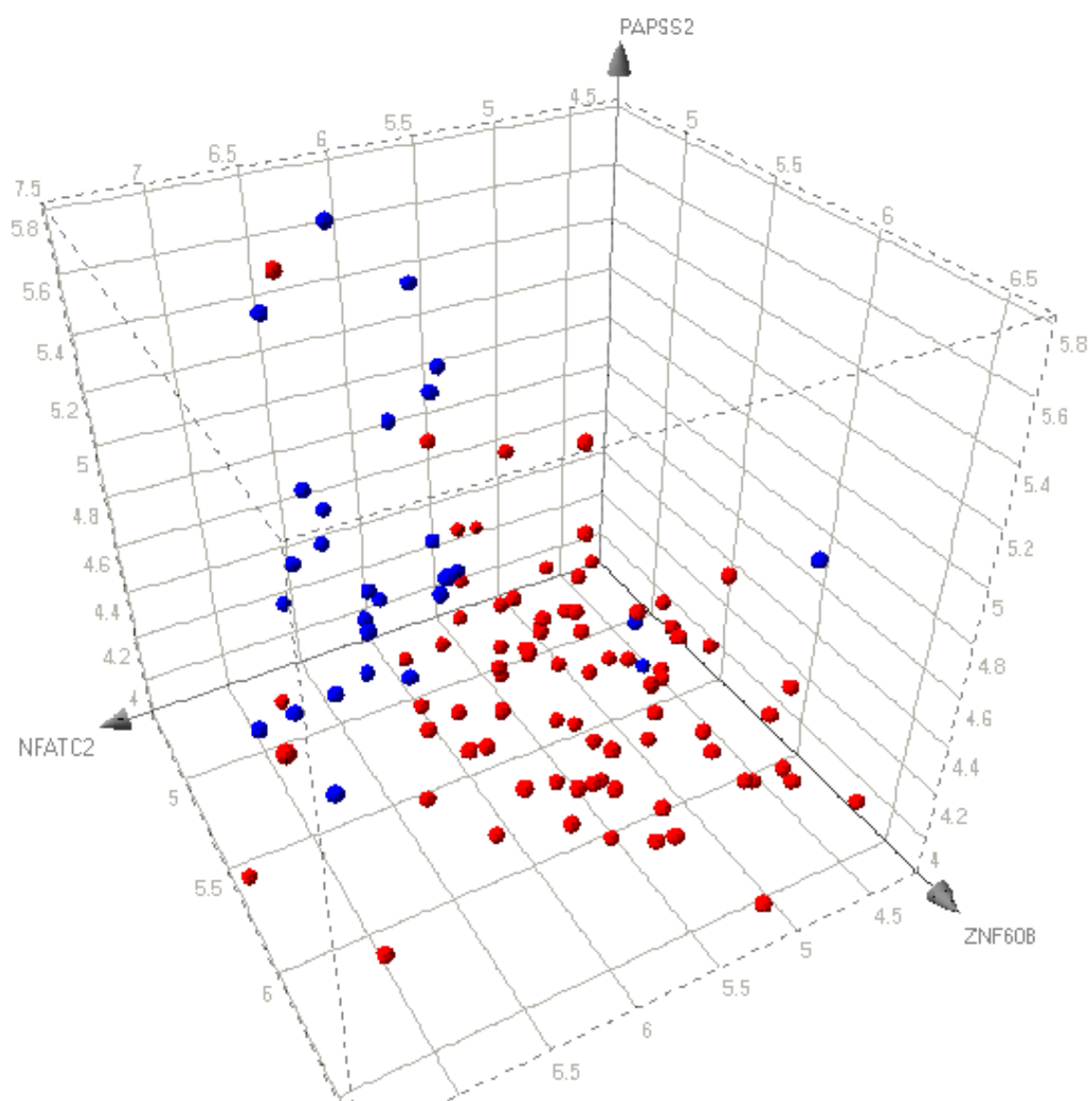

Supplement: Figure S1 — Expression level of three genes (NFATC2, PAPSS2, and ZNP608) for controls and probands. (A) 17 healthy controls and 14 severely affected CdLS probands with NIPBL protein truncating mutations, and (B) 101-sample cohort used for target array analysis including the same individuals as in (A). Three axes represent expression of the three genes, blue dots represent controls, including healthy participants and individuals with other genetic diagnoses; red dots represent CdLS probands. (0.26 MB PDF) [file pbio.1000119.s001.pdf]

Figure S2.

A

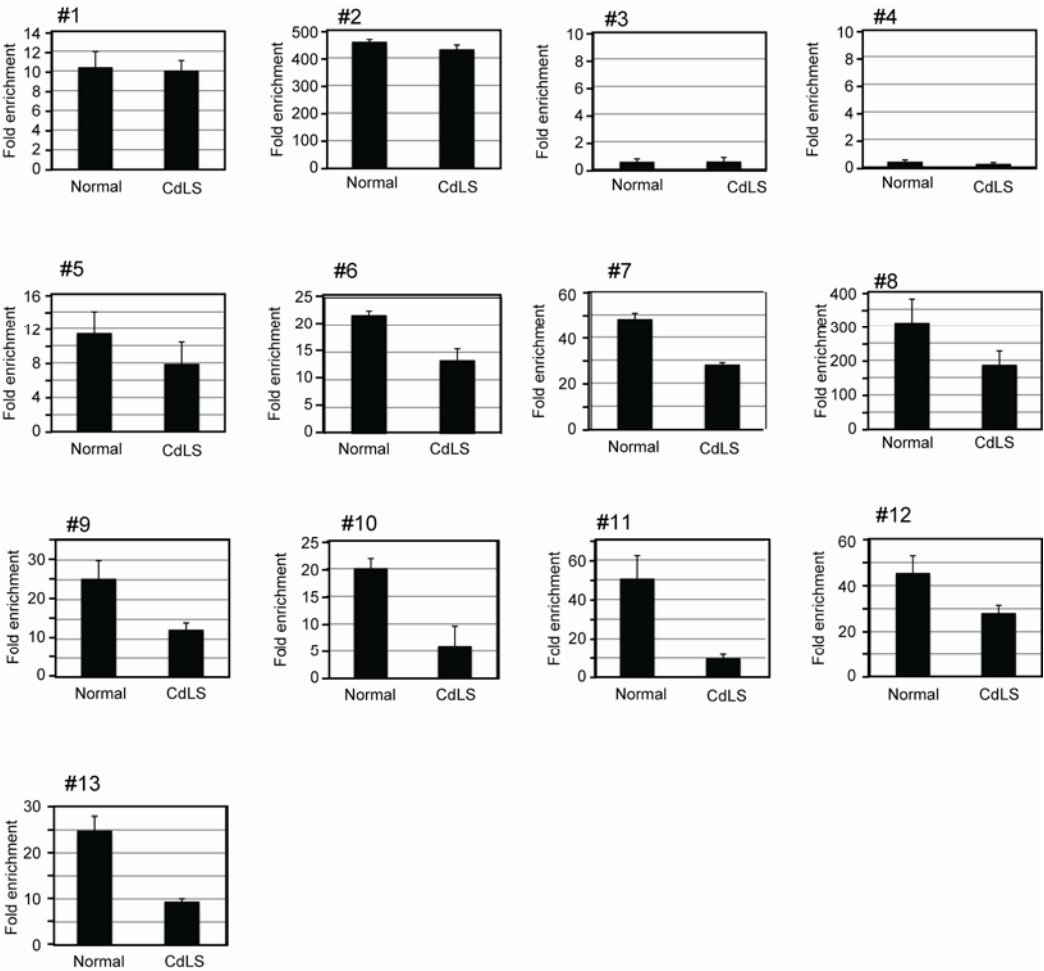

B

## Average cohesin binding

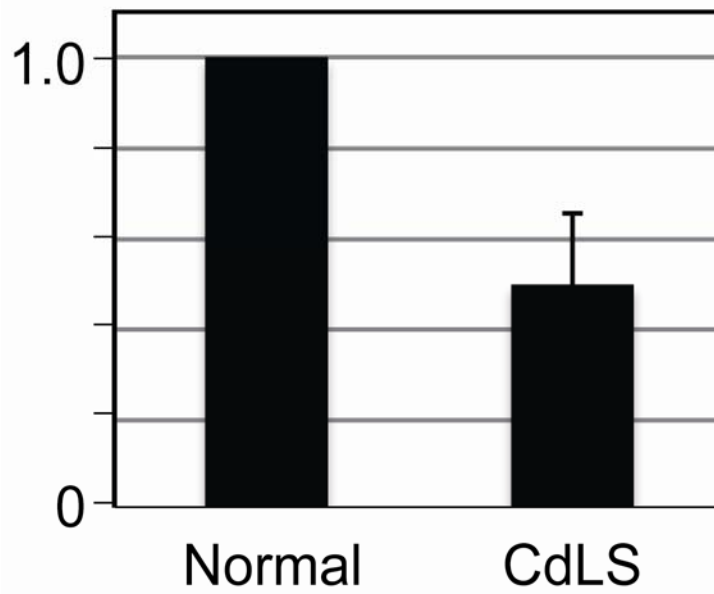

Supplement: Figure S2 — ChIP-qPCR validation of 13 cohesin binding sites identified by ChIP array. RAD21 ChIP samples were obtained from the CdLS proband and the control in the ChIP array studies and were analyzed by qPCR for the presence of 13 different cohesin binding sites with site-specific primers (mean of n = 3; error bars +/− standard deviation [SD]) (see Table S11 for genomic addresses of the 13 sites and primer sequences). The results were presented as fold-enrichment over control ChIP (nonantibody). (A) The presence of cohesin binding at 13 examined genomic sites, sites 1 and 2 were bound equally by cohesin in both probands and control in the array studies and served as positive controls here; sites 3 and 4 did not demonstrate cohesin binding in either proband or control in the array studies and served as negative controls here; sites 5–13 are nine genomic sites where cohesin binding was lost in the CdLS cells by qualitative analysis in the array studies. Quantitative PCR has revealed the amount of cohesin bound to these sites is significantly reduced at all of the examined loci. (B) Quantitative analysis of average amount of cohesin bound to the nine examined sites revealed at least half of cohesin binding is lost in the CdLS cells. (0.36 MB PDF) [file pbio.1000119.s002.pdf]

Figure S3.

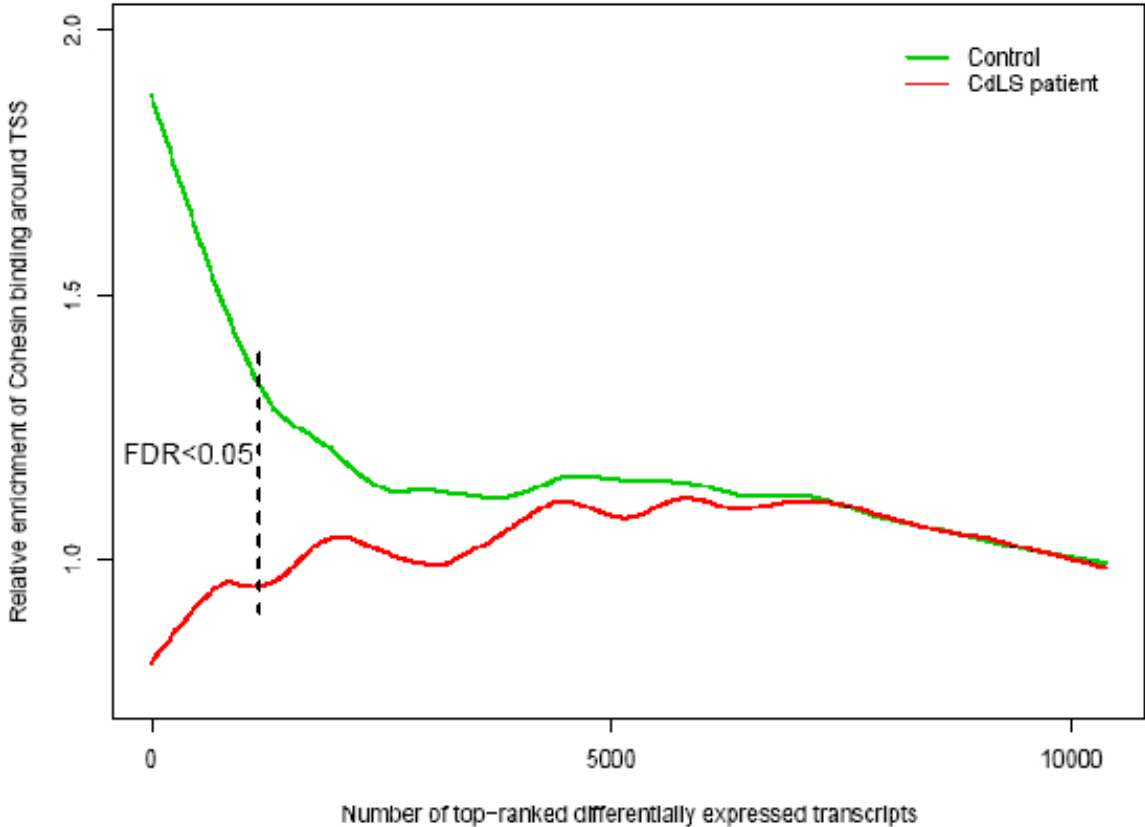

Supplement: Figure S3 — Cohesin binding within +/− 100 bp around TSSs is enriched in differentially expressed genes. The 10,378 unique genes expressed in LCLs are ranked by their F scores. The reference enrichment is the overall percentage of genes having cohesin binding within 200 bp (+/− 100 bp) around TSSs. The relative enrichment is calculated as the value of cohesin binding enrichment in top-ranked genes over the reference enrichment. The relative enrichment point is calculated for the total number of genes prior to the point on the x-axis. The numbers on x-axis denote the number of top-ranked genes. The curves are smoothed by the LOWESS algorithm. (0.23 MB PDF) [file pbio.1000119.s003.pdf]

Figure S4.

A

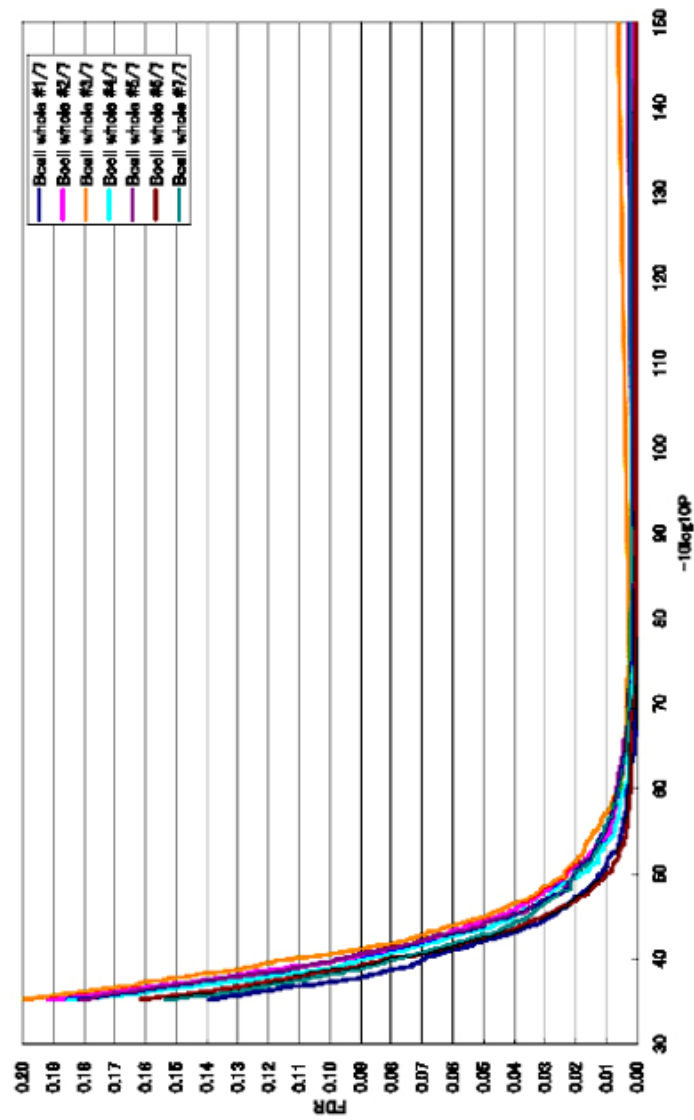

B

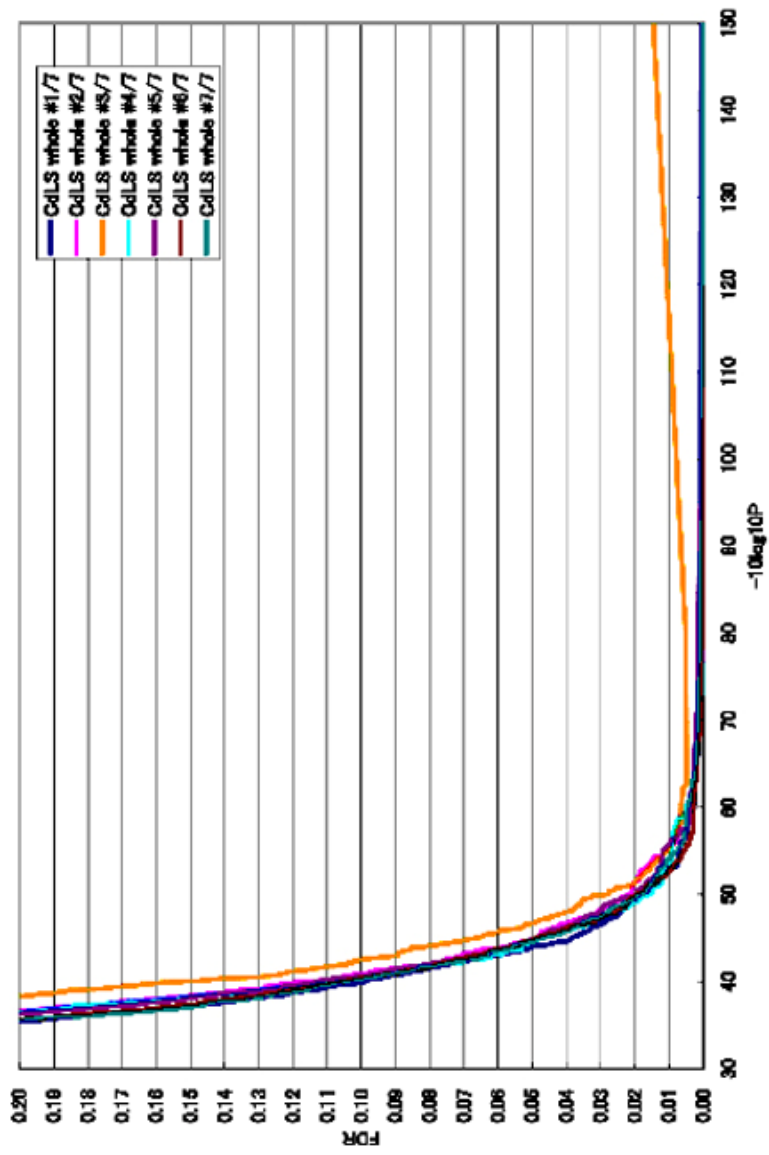

Supplement: Figure S4 — FDRs of genome-wide ChIP microarrays of (A) controls and (B) CdLS proband. The x-axis denotes the p-values and the y-axis denotes the average FDR percentage for each experiment. Note that the FDR is less than 1% at the threshold p-value = 10−6 adopted for the analyses performed in this study. (0.33 MB PDF) [file pbio.1000119.s004.pdf]
