# Supplementary material for: Transcriptional Dysregulation in NIPBL and Cohesin Mutant Human Cells
Source: PLoS Biol. 2009 May 26;7(5):e1000119. doi: 10.1371/journal.pbio.1000119 (PMC2680332; doi:10.1371/journal.pbio.1000119)
Supplement: Table S1 — 39 training and testing samples were used for the whole genome expression array analyses. (A) 16 LCL samples from severely affected CdLS probands with identified protein-truncating mutations of NIPBL were used for the training set. (B) 17 LCL samples from healthy controls were also included in the training set for expression array analyses. An additional six samples were used as a testing set. (0.23 MB PDF) [file pbio.1000119.s005.pdf]

Table S1. Thirty nine training and testing samples were used for the whole genome expression array analyses. (A) 16 LCL samples from severely affected Cornelia de Lange Syndrome probands with identified protein truncating mutations of *NIPBL* were used for the training set. (B) 17 LCL samples from healthy controls were also included in the training set for expression array analyses. An additional 6 samples were used as a testing set.

A

16 CdLS Probands (Training set)*				
**ID**	**Gender**	**Age(Yr)**	**Genotype****	**Clinical Evaluation /Picture Availability**
PT2	M	0	c.2449_2450insG / Frameshift	Severe / Yes
PT3	F	0	c.1444_1447delAGAG / Frameshift	Severe / No
PT5	F	0	c.3048_3051delATTA / Frameshift	Severe / Yes
PT12	M	0.3	c.150delG / Nonsense	Severe / No
PT15	M	0.8	c.1934delA / Nonsense	Severe / Yes
PT7	F	5.3	c.4193C>G / Nonsense	Severe / Yes
PT11	M	6.7	c.1902_1903insA / Frameshift	Severe / Yes
PT1	M	6.9	c.6407insA / Frameshift	Severe / Yes
PT9	F	7	c.742_743delCT / Frameshift	Severe / Yes
PT13	F	8.3	c.2494C>T / nonsense	Severe / Yes
PT16	F	9	c.4606C>T / Nonsense	Severe / No
PT10	M	10.5	c.2969delG / Frameshift	Severe / Yes
PT6	M	11.9	c.5167C>T / Nonsense	Severe / No
PT8	F	14.6	c.961delA / Frameshift	Severe / Yes
PT4	M	19.5	c.4606C>T / Nonsense	Severe / Yes
PT14	F	23.5	c.1546_1547insG / Frameshift	Severe / Yes

* All 16 probands are Caucasian

** Nucleotide numbering refers to the *NIPBL B* isoform cDNA sequence with GeneBank accession number NM_015384 and starting at the +1 position of the translation initiation codon

B

	ID	Gender	Age(Yr)	Genotype
17 healthy controls	N7	F	1.5	WT
	N11	F	2	WT
	N12	M	3.5	WT
	N9	M	4.5	WT
	N13	M	4.5	WT
	N8	M	5	WT
	N10	F	6.8	WT
	N5	F	7	WT
	N4	M	8	WT
	N6	M	8	WT
	N1	F	10	WT
	N17	F	10	WT
	N2	F	12	WT
	N14	M	12	WT
	N18	M	13	WT
	N16	M	14	WT
	N15	F	18.5	WT
Test set of 6 samples	PT007 *	M	0	c.4002_4005del4
	N007	M	7	WT
	AGS1	M	7	*JAG1* mutation
	AGS2	M	8	*JAG1* mutation
	RS1**	N/A	N/A	*ESCO2* mutation
	RS2**	N/A	N/A	*ESCO2* mutation

*Egyptian
** Races not known
