# Supplementary material for: Transcriptional Dysregulation in NIPBL and Cohesin Mutant Human Cells
Source: PLoS Biol. 2009 May 26;7(5):e1000119. doi: 10.1371/journal.pbio.1000119 (PMC2680332; doi:10.1371/journal.pbio.1000119)
Supplement: Table S2 — 1,915 probe sets representing 1,501 unique genes (FDR<0.05) are differentially expressed in CdLS. (0.35 MB PDF) [file pbio.1000119.s006.pdf]

Table S2. 1915 probe sets representing 1501 unique genes (FDR<0.05) are differentially expressed in CdLS.

Probe set	Gene symbol	Fold change	Rank	F_Score	FDR
213918_s_at	NIPBL	-1.338855257	1	51.26727771	0
219911_s_at	SLCO4A1	-1.612165663	2	50.95602425	0
204394_at	SLC43A1	-1.706452196	3	49.90491963	0
207108_s_at	NIPBL	-1.367935304	4	49.48880641	0
203060_s_at	PAPSS2	-3.386981249	5	45.73156232	0
203333_at	KIFAP3	1.411275843	6	45.71065661	0
205204_at	NMB	-1.172022284	7	41.14379226	0
37549_g_at	PTHB1	1.336074078	8	40.55147469	0
226580_at	BRMS1L	1.522033381	9	40.35821249	0
216388_s_at	LTB4R	-1.339783602	10	40.14798445	0
244467_at	LOC440829	-2.653690281	11	39.86365319	0
215438_x_at	GSPT1	-1.171210181	12	39.34348686	0
229332_at	GLOXD1	-2.363623094	13	39.24433603	0
204866_at	PHF16	3.853047576	14	37.31056714	0
218491_s_at	THYN1	-1.224336392	15	37.14597784	0
225914_s_at	CAB39L	1.775222675	16	35.96680168	0.000625
205352_at	SERPINI1	1.760518027	17	35.89390909	0.000588235
226611_s_at	PRR6	-1.831467373	18	35.84550039	0.000555556
225347_at	ARL8A	1.281647924	19	35.01840122	0.000526316
223711_s_at	THYN1	-1.183451022	20	34.80256116	0.0005
217995_at	SQRDL	1.36983298	21	34.37458245	0.00047619
203836_s_at	MAP3K5	1.558329159	22	33.89583134	0.000454545
207937_x_at	FGFR1	-1.223488041	23	33.66751254	0.000434783
212240_s_at	PIK3R1	1.36983298	24	33.42133732	0.000416667
231843_at	DDX55	-1.251796459	25	33.31191957	0.0004
209861_s_at	METAP2	-1.194991205	26	33.14421887	0.000769231
224648_at	GPBP1	1.251796459	27	32.49506487	0.001481481
205685_at	CD86	1.660940048	28	32.4700268	0.001428571
225639_at	SKAP2	1.445932295	29	32.40082767	0.00137931
225403_at	C9orf23	-1.232852325	30	32.10052053	0.001666667
225564_at	SPATA13	1.70054832	31	31.90737197	0.001612903
201522_x_at	SNRPN	1.25962998	32	31.58157057	0.001875
201952_at	ALCAM	1.375541818	33	31.41833745	0.002121212
204362_at	SKAP2	1.476314406	34	31.24375264	0.002058824
209079_x_at	PCDHGC3	-2.060507907	35	31.08842018	0.002
201889_at	FAM3C	1.675974269	36	30.83722507	0.001944444
212335_at	GNS	1.36983298	37	30.60804409	0.001891892
218993_at	RNMTL1	-1.145517898	38	30.36491347	0.001842105
201658_at	ARL1	1.210833084	39	30.29742196	0.001794872
204601_at	N4BP1	1.29145735	40	30.2733882	0.00175
208910_s_at	C1QBP	-1.262252032	41	30.1863054	0.001707317
225777_at	C9orf140	-1.608816742	42	30.09410665	0.001666667
221752_at	SSH1	1.480413298	43	29.64105821	0.001860465
226991_at	NFATC2	-2.114036081	44	29.57889247	0.001818182
200903_s_at	AHCY	-1.238848698	45	29.48574536	0.001777778
214214_s_at	C1QBP	-1.186736798	46	29.34170808	0.00173913

218883_s_at	MLF1IP	-1.333298677	47	29.23895026	0.001702128
228764_s_at	CHMP4A	1.219255094	48	29.22663814	0.001666667
221808_at	RAB9	1.565908593	49	29.08728343	0.001836735
212959_s_at	GNPTAB	1.473247686	50	29.07118479	0.0018
215836_s_at	PCDHGC3	-2.51925996	51	29.05528871	0.001764706
218104_at	TEX10	-1.199971382	52	28.99589877	0.001730769
205717_x_at	PCDHGC3	-2.597277205	53	28.95480313	0.001698113
219734_at	SIDT1	1.492778383	54	28.86726422	0.001666667
229828_at	AL044007	1.257013375	55	28.74280655	0.002
226610_at	PRR6	-1.717130873	56	28.56942907	0.001964286
218017_s_at	HGSNAT	1.489677463	57	28.49919824	0.002105263
207966_s_at	GLG1	1.276328769	58	28.30965716	0.002068966
200945_s_at	SEC31A	1.186736798	59	28.29606245	0.002033898
1569346_a_at	P2RX1	1.284315809	60	28.22970999	0.002
201770_at	SNRPA	-1.175276328	61	28.22604739	0.001967213
222692_s_at	FNDC3B	1.890804234	62	28.22599806	0.001935484
222693_at	FNDC3B	1.731473131	63	28.1701619	0.001904762
220768_s_at	CSNK1G3	1.257884972	64	28.13006931	0.001875
229817_at	ZNF608	2.197708435	65	28.11380396	0.001846154
218512_at	WDR12	-1.192508872	66	28.01444642	0.001969697
201847_at	LIPA	1.356604327	67	27.79513734	0.001940299
212291_at	HIPK1	1.320422841	68	27.70844328	0.001911765
211066_x_at	PCDHGC3	-2.50185816	69	27.68494306	0.001884058
227279_at	TCEAL3	1.346300069	70	27.6548758	0.001857143
228977_at	LOC729680	-1.573524891	71	27.59024184	0.001830986
208953_at	LARP5	1.148698355	72	27.55845076	0.001944444
225924_at	KIAA1450	2.620786808	73	27.48875342	0.001917808
218618_s_at	FNDC3B	2.581124981	74	27.30933593	0.002027027
210788_s_at	DHRS7	1.356604327	75	27.26192412	0.002
201813_s_at	TBC1D5	1.315854525	76	27.20296012	0.001973684
201951_at	ALCAM	1.379360922	77	27.17666771	0.001948052
212249_at	PIK3R1	1.383190629	78	27.07832288	0.001923077
219274_at	TSPAN12	2.979354926	79	27.07492539	0.001898734
227525_at	GLCCI1	1.472226862	80	27.04046532	0.001875
208093_s_at	NDEL1	1.180174343	81	26.81227922	0.001851852
225032_at	FNDC3B	2.586497864	82	26.71709853	0.002073171
201814_at	TBC1D5	1.286989247	83	26.6948926	0.002168675
212293_at	HIPK1	1.368883813	84	26.68020937	0.002142857
203867_s_at	NLE1	-1.248330549	85	26.56744941	0.002235294
221753_at	SSH1	1.515716567	86	26.54388262	0.002209302
216239_at	PTHB1	1.45296505	87	26.29818582	0.002413793
202975_s_at	RHOBTB3	2.533268507	88	26.26586562	0.002386364
208660_at	CS	-1.131314463	89	26.26365401	0.002359551
1564907_s_at	MATR3	-1.385109468	90	26.15711606	0.002555556
201614_s_at	RUVBL1	-1.229438867	91	26.12185127	0.002527473
233759_s_at	SMEK2	1.131314463	92	26.09526628	0.0025
216020_at	IFIH1	1.497960934	93	25.99360264	0.002473118
212846_at	KIAA0179	-1.276328769	94	25.9690039	0.002446809
218909_at	RPS6KC1	1.244874235	95	25.92280809	0.002421053
202559_x_at	C1orf77	-1.091263877	96	25.88979724	0.002395833
224714_at	MKI67IP	-1.149494848	97	25.85859268	0.002371134

223917_s_at	SLC39A3	-1.349102534	98	25.787545	0.002346939
242714_at	AW500340	3.088700532	99	25.73532558	0.002323232
241937_s_at	WDR4	-1.375541818	100	25.71329301	0.0024
210115_at	RPL39L	-1.319507911	101	25.66391721	0.002376238
218081_at	C20orf27	-1.319507911	102	25.65857318	0.002352941
225706_at	GLCCI1	1.409320755	103	25.58310088	0.002330097
224046_s_at	PDE7A	-1.367935304	104	25.39399583	0.002692308
201196_s_at	AMD1	-1.21167266	105	25.21385942	0.003142857
203882_at	ISGF3G	1.304050735	106	25.1599587	0.003113208
32069_at	N4BP1	1.296839555	107	25.07026102	0.003084112
235347_at	LRCH3	1.296839555	108	25.00559122	0.003240741
209917_s_at	TP53AP1	1.373636233	109	24.9940594	0.003302752
212733_at	KIAA0226	1.248330549	110	24.84382617	0.003818182
213049_at	GARNL1	1.294145654	111	24.82992881	0.003783784
222024_s_at	AKAP13	1.301341855	112	24.69837427	0.003928571
210224_at	MR1	1.282536603	113	24.68539264	0.003893805
220762_s_at	GNB1L	-1.162314108	114	24.67062632	0.003859649
201328_at	ETS2	-1.285206337	115	24.63085815	0.003826087
213194_at	ROBO1	4.61714167	116	24.59570003	0.004137931
213669_at	FCHO1	-1.351910833	117	24.58794676	0.004102564
212355_at	KIAA0323	1.272794935	118	24.49715831	0.004237288
203758_at	CTSO	1.422077411	119	24.42806809	0.004285714
205483_s_at	ISG15	1.830198336	120	24.42019902	0.00425
229305_at	MLF1IP	-1.478362431	121	24.41424292	0.004297521
210886_x_at	TP53AP1	1.340712592	122	24.37418317	0.004344262
210973_s_at	FGFR1	-2.826467288	123	24.34165321	0.004390244
228442_at	AI770171	-2.061936638	124	24.19751943	0.004435484
205733_at	BLM	-1.273677475	125	24.14487917	0.0044
209036_s_at	MDH2	-1.080725402	126	23.98848018	0.004603175
230626_at	TSPAN12	2.292624371	127	23.97367421	0.004566929
202085_at	TJP2	1.342572503	128	23.91075361	0.0046875
212742_at	ZNF364	1.155085785	129	23.86216208	0.004728682
208759_at	IKBKB	1.220946513	130	23.85677358	0.004692308
203173_s_at	MGC16824	1.179356592	131	23.8484689	0.004656489
202304_at	FNDC3A	1.348167732	132	23.82970892	0.00469697
217737_x_at	C20orf43	1.118837101	133	23.80120169	0.004661654
210241_s_at	TP53AP1	1.345367209	134	23.76947784	0.004701493
212441_at	KIAA0232	1.230291345	135	23.76316913	0.004666667
206037_at	CCBL1	-1.155886707	136	23.72382531	0.004632353
218058_at	CXXC1	-1.427014506	137	23.65260951	0.00459854
203291_at	CNOT4	1.169587664	138	23.53360052	0.004710145
208089_s_at	TDRD3	1.163120042	139	23.50236842	0.004748201
209664_x_at	NFATC1	-1.508380077	140	23.50145283	0.004714286
207565_s_at	MR1	1.227735684	141	23.4906277	0.004680851
213238_at	ATP10D	1.712376569	142	23.44158358	0.00471831
235583_at	ILDR1	1.687631592	143	23.32824533	0.004825175
201563_at	SORD	-1.375541818	144	23.29134242	0.004861111
220588_at	BCAS4	-1.433955248	145	23.27616519	0.004827586
218085_at	CHMP5	1.267512522	146	23.26927298	0.004794521
212625_at	STX10	-1.254402205	147	23.21253581	0.004897959
226249_at	SNX30	-1.479387509	148	23.20806373	0.005

208680_at	PRDX1	-1.194991205	149	23.18379298	0.004966443
225922_at	KIAA1450	2.672148157	150	23.16302267	0.004933333
236835_at	LOC645431	-1.647182035	151	23.15824982	0.004900662
203384_s_at	GOLGA1	1.243149669	152	23.09897685	0.005065789
221920_s_at	SLC25A37	-1.654046737	153	23.08852254	0.005098039
235509_at	C8orf38	-1.275444392	154	23.00920233	0.00525974
224460_s_at	L2HGDH	-1.303147149	155	23.0073584	0.005225806
202180_s_at	MVP	1.576800348	156	22.88456453	0.00525641
212543_at	AIM1	1.776453592	157	22.81685688	0.005414013
226882_x_at	WDR4	-1.618884433	158	22.79479835	0.005379747
209679_s_at	LOC57228	-1.922521857	159	22.74800409	0.005345912
225858_s_at	BIRC4	1.281647924	160	22.73632882	0.0053125
212735_at	KIAA0226	1.225185332	161	22.69011357	0.005403727
223134_at	BBX	1.293248932	162	22.68285595	0.00537037
203724_s_at	RUFY3	1.783857039	163	22.68031183	0.005337423
231517_at	ZYG11A	-1.880348405	164	22.68029788	0.005304878
1554015_a_at	CHD2	1.215036792	165	22.66391269	0.005272727
224610_at	SNHG1	-1.195819797	166	22.53445388	0.005481928
200701_at	NPC2	1.264879542	167	22.4738458	0.005449102
209534_x_at	AKAP13	1.264879542	168	22.46393358	0.00547619
202468_s_at	CTNNAL1	-1.370782805	169	22.44138575	0.005443787
226196_s_at	C14orf179	1.313121125	170	22.4324522	0.005411765
201930_at	MCM6	-1.278099363	171	22.4289937	0.005380117
203885_at	RAB21	1.220946513	172	22.4215868	0.005348837
228252_at	PIF1	-1.268391399	173	22.3963584	0.005433526
219110_at	NOLA1	-1.196648963	174	22.3959917	0.005402299
222514_at	RRAGC	1.231998073	175	22.38547768	0.005371429
210251_s_at	RUFY3	1.315854525	176	22.33668411	0.005340909
203227_s_at	TSPAN31	1.347233577	177	22.27325767	0.005367232
217733_s_at	TMSB10	1.185914499	178	22.26222683	0.005393258
221953_s_at	MMP24	-1.124278924	179	22.18802139	0.005363128
204308_s_at	KIAA0329	1.599920257	180	22.180312	0.005333333
202149_at	NEDD9	1.693490625	181	22.14393905	0.005359116
212310_at	MIA3	1.279872414	182	22.13853003	0.00532967
226008_at	NDNL2	1.568080908	183	22.11508637	0.005409836
226230_at	SMEK2	1.157490217	184	22.10629581	0.005380435
226460_at	KIAA1450	2.830388321	185	22.05442944	0.005405405
229270_x_at	LOC646044	-1.387992719	186	22.03328766	0.005376344
238510_at	ZNF720	1.249196126	187	22.02434756	0.00540107
209447_at	SYNE1	1.506290467	188	21.99176963	0.00537234
225074_at	RAB2B	1.275444392	189	21.97229885	0.005396825
214059_at	IFI44	1.721898377	190	21.96362182	0.005421053
207571_x_at	C1orf38	1.682958965	191	21.94836423	0.00539267
1555832_s_at	KLF6	1.463071221	192	21.90817083	0.005572917
217043_s_at	SYT7	1.32592576	193	21.86105937	0.005751295
231866_at	LNPEP	1.481439798	194	21.82444257	0.005721649
202144_s_at	ADSL	-1.125838586	195	21.82191512	0.005692308
218324_s_at	SPATS2	1.20163605	196	21.79946982	0.005765306
230625_s_at	TSPAN12	2.168954818	197	21.76923758	0.005786802
226713_at	CCDC50	1.55293775	198	21.75071293	0.005757576
223705_s_at	GPBP1	1.20163605	199	21.73302725	0.005778894

202395_at	NSF	1.367935304	200	21.73158652	0.00575
214299_at	TOP3A	-1.162314108	201	21.72603252	0.005721393
213271_s_at	DOPEY1	1.364147835	202	21.62729376	0.005891089
211801_x_at	SYT7	1.316766922	203	21.598363	0.005862069
211535_s_at	FGFR1	-3.652793	204	21.54830582	0.005882353
214855_s_at	GARNL1	1.277213759	205	21.54780633	0.005853659
210895_s_at	CD86	2.015307521	206	21.48200043	0.005873786
208920_at	SRI	1.658639092	207	21.47244525	0.005845411
216048_s_at	RHOBTB3	2.190104942	208	21.3213444	0.00625
212334_at	GNS	1.383190629	209	21.30381292	0.006267943
224326_s_at	PCGF6	-1.109569472	210	21.2879035	0.006285714
201393_s_at	IGF2R	1.294145654	211	21.2727173	0.006255924
1558381_a_at	GAPDHS	-1.176906737	212	21.25779932	0.006226415
203159_at	GLS	1.322254605	213	21.23781666	0.006197183
210044_s_at	LYL1	-1.566994374	214	21.23403738	0.006168224
219648_at	MREG	1.351910833	215	21.20250586	0.006186047
217743_s_at	TMEM30A	1.187559666	216	21.17776668	0.006203704
201457_x_at	BUB3	-1.165541198	217	21.16324682	0.006175115
209090_s_at	SH3GLB1	1.285206337	218	21.11298884	0.00646789
201989_s_at	CREBL2	1.250062303	219	21.06341822	0.006621005
208030_s_at	ADD1	1.265756594	220	21.03861284	0.006636364
212807_s_at	SORT1	1.86218964	221	21.03401253	0.006606335
212467_at	DNAJC13	1.097331938	222	21.03323929	0.006576577
204361_s_at	SKAP2	1.41029796	223	21.02747895	0.006547085
204286_s_at	PMAIP1	1.4054187	224	20.98871534	0.0065625
210616_s_at	SEC31A	1.225185332	225	20.98082621	0.006533333
210223_s_at	MR1	1.221793102	226	20.97971479	0.006504425
221519_at	FBXW4	1.182631	227	20.92696952	0.00660793
224722_at	MIB1	1.395710764	228	20.91704991	0.006578947
208961_s_at	KLF6	1.393777239	229	20.90234704	0.006550218
222613_at	C12orf4	1.148698355	230	20.8958541	0.006521739
213132_s_at	MCAT	-1.180174343	231	20.89467437	0.006493506
206074_s_at	HMGA1	-1.254402205	232	20.83761319	0.006508621
205403_at	IL1R2	3.179941004	233	20.82350762	0.006480687
202121_s_at	CHMP2A	1.159899655	234	20.78601141	0.006581197
217854_s_at	POLR2E	-1.137605228	235	20.77608382	0.006553191
224502_s_at	KIAA1191	1.260503392	236	20.7113424	0.006737288
225957_at	LOC153222	1.411275843	237	20.70587141	0.006793249
201988_s_at	CREBL2	1.222640278	238	20.69812101	0.006764706
201392_s_at	IGF2R	1.337927555	239	20.66690447	0.006820084
201391_at	TRAP1	-1.264879542	240	20.65908646	0.006833333
209091_s_at	SH3GLB1	1.190031696	241	20.60549152	0.006887967
221156_x_at	CCPG1	1.550786413	242	20.5950433	0.006859504
235051_at	CCDC50	1.555092072	243	20.54353203	0.00691358
235830_at	NT5DC1	1.300440147	244	20.53700112	0.006885246
220507_s_at	UPB1	1.394743666	245	20.52376531	0.006857143
203247_s_at	ZNF24	1.140763716	246	20.48734056	0.006910569
205264_at	CD3EAP	-1.375541818	247	20.47831904	0.006923077
202150_s_at	NEDD9	1.572434584	248	20.47187241	0.006895161
227701_at	C10orf118	1.33422317	249	20.46328075	0.006907631
203837_at	MAP3K5	1.478362431	250	20.44942087	0.00688

224957_at	LOC497661	1.181811547	251	20.44166374	0.00685259
222230_s_at	ACTR10	1.104964485	252	20.40981569	0.006944444
212006_at	UBXD2	1.159899655	253	20.39259011	0.006916996
203206_at	FAM53B	-1.366040257	254	20.33311152	0.007047244
203226_s_at	TSPAN31	1.248330549	255	20.26628576	0.007098039
209750_at	NR1D2	1.336074078	256	20.25447511	0.007070313
200083_at	USP22	-1.097331938	257	20.2296233	0.007120623
218132_s_at	TSEN34	1.158292806	258	20.21127462	0.007093023
228787_s_at	BCAS4	-1.411275843	259	20.20789408	0.007065637
202962_at	KIF13B	1.295940965	260	20.20505986	0.007038462
222498_at	AZI2	1.21335356	261	20.19697659	0.007011494
222408_s_at	YPEL5	1.53368266	262	20.19175886	0.006984733
218141_at	UBE2O	-1.17609125	263	20.18046276	0.006996198
203596_s_at	IFIT5	1.484523571	264	20.15757782	0.007007576
204780_s_at	FAS	1.429984986	265	20.11531807	0.007018868
243745_at	AP1S2	-1.319507911	266	20.10492009	0.006992481
219901_at	FGD6	-2.164449289	267	20.10436945	0.006966292
219458_s_at	NSUN3	1.147107024	268	20.09540575	0.006940299
228693_at	CCDC50	1.417157397	269	20.08377816	0.006914498
205105_at	MAN2A1	2.133171562	270	20.07080661	0.006925926
238012_at	DPP7	-1.309485423	271	20.06966573	0.006900369
200673_at	LAPTM4A	1.204137381	272	20.05688577	0.006875
226538_at	MAN2A1	2.087823855	273	20.03158057	0.006849817
223422_s_at	ARHGAP24	2.895876345	274	20.00954418	0.00689781
214453_s_at	IFI44	1.555092072	275	19.99063673	0.006872727
236080_at	BE276063	-1.152686347	276	19.98223312	0.006847826
229146_at	C7orf31	1.245737416	277	19.94673125	0.006895307
229865_at	FNDC3B	1.771535038	278	19.91524854	0.006870504
212282_at	TMEM97	-1.325007017	279	19.87039937	0.006989247
211623_s_at	FBL	-1.136029265	280	19.85651392	0.007
204573_at	CROT	1.48246701	281	19.8478187	0.007010676
208273_at	ZNF695	-1.798756624	282	19.84032014	0.006985816
203866_at	NLE1	-1.124278924	283	19.83767089	0.006961131
204198_s_at	RUNX3	1.254402205	284	19.83414097	0.00693662
201133_s_at	PJA2	1.130530567	285	19.79426282	0.007017544
218020_s_at	ZFAND3	1.252664439	286	19.7930056	0.006993007
223177_at	GLI3	1.215036792	287	19.785002	0.006968641
218043_s_at	AZI2	1.231144413	288	19.77436931	0.007048611
214749_s_at	ARMCX6	1.136029265	289	19.76501121	0.007024221
225700_at	GLCCI1	1.4054187	290	19.73513956	0.007068966
231927_at	ATF6	1.267512522	291	19.70979744	0.007113402
202318_s_at	SENP6	1.141554707	292	19.6735369	0.007123288
201892_s_at	IMPDH2	-1.172834949	293	19.60304307	0.007303754
222281_s_at	AW517716	2.781763943	294	19.60149288	0.007278912
212400_at	FAM102A	-1.486582984	295	19.59988563	0.007254237
226682_at	LOC283666	4.019455282	296	19.58972732	0.00722973
205920_at	SLC6A6	-1.551861709	297	19.58452941	0.007205387
212048_s_at	YARS	-1.165541198	298	19.58335954	0.007181208
222360_at	DPH5	-1.171210181	299	19.5723672	0.007157191
225522_at	AAK1	1.314031627	300	19.5614363	0.007166667
204033_at	TRIP13	-1.270150983	301	19.55146743	0.00717608

213245_at	ADCY1	-2.768299432	302	19.51717354	0.007284768
222593_s_at	SPATS2	1.222640278	303	19.5136846	0.007260726
1555137_a_at	FGD6	-2.197708435	304	19.46101095	0.007368421
227767_at	CSNK1G3	1.29056249	305	19.44168948	0.007442623
227697_at	SOCS3	2.781763943	306	19.42198031	0.00748366
218136_s_at	SLC25A37	-1.282536603	307	19.41480924	0.00752443
226391_at	NDUFB2	1.276328769	308	19.34540368	0.007694805
219863_at	HERC5	1.43296165	309	19.34082214	0.007669903
202284_s_at	CDKN1A	1.325007017	310	19.32058948	0.007677419
204510_at	CDC7	-1.281647924	311	19.30145813	0.007717042
222235_s_at	GALNACT-2	1.341642225	312	19.25763664	0.007948718
213581_at	PDCD2	-1.184271612	313	19.24406306	0.007923323
226799_at	AK026881	-1.79129134	314	19.23736867	0.007929936
222781_s_at	C9orf40	-1.283425898	315	19.23388155	0.007904762
201272_at	AKR1B1	-1.115739322	316	19.20225276	0.008006329
207826_s_at	ID3	-1.775222675	317	19.19192054	0.008012618
225331_at	CCDC50	1.366987452	318	19.17511459	0.008050314
209421_at	MSH2	-1.289668251	319	19.15533211	0.008056426
224404_s_at	FCRL5	2.726405215	320	19.15368155	0.00803125
206106_at	MAPK12	-1.370782805	321	19.15267996	0.008006231
212341_at	YIPF6	1.193335743	322	19.15111724	0.007981366
218048_at	COMMD3	1.266634254	323	19.13389498	0.007987616
1554806_a_at	FBXO8	1.224336392	324	19.10906782	0.00808642
228205_at	TKT	-1.145517898	325	19.08936923	0.008061538
227968_at	PDDC1	-1.176906737	326	19.08079019	0.00803681
205641_s_at	TRADD	1.300440147	327	19.07747423	0.008042813
223209_s_at	SELS	1.43893358	328	19.04714135	0.008109756
202976_s_at	RHOBTB3	2.494931144	329	19.03967571	0.008176292
204285_s_at	PMAIP1	1.366040257	330	19.00035811	0.00830303
226267_at	JDP2	-1.52414483	331	19.00014596	0.008277946
213521_at	PTPN18	-1.387030969	332	18.9956032	0.008253012
223018_at	NOB1	-1.125838586	333	18.98187324	0.008228228
211729_x_at	BLVRA	1.529436278	334	18.91383645	0.008413174
210785_s_at	C1orf38	1.69466487	335	18.91323429	0.00838806
238520_at	TRERF1	-2.355445579	336	18.88238073	0.008422619
212498_at	AF056433	1.219255094	337	18.83775704	0.008694362
218590_at	PEO1	-1.144724161	338	18.83375621	0.008668639
202788_at	MAPKAPK3	-1.317679952	339	18.81371906	0.008672566
201641_at	BST2	1.212512819	340	18.77507273	0.008735294
201990_s_at	CREBL2	1.362258035	341	18.75992253	0.008826979
223738_s_at	PGM2	-1.095811766	342	18.7578217	0.00880117
201193_at	IDH1	1.232852325	343	18.75110687	0.00877551
213073_at	ZFYVE26	1.308578071	344	18.75104208	0.00875
208783_s_at	CD46	1.189207115	345	18.74039468	0.008724638
206175_x_at	ZNF222	1.194163187	346	18.73654017	0.008757225
201851_at	SH3GL1	1.156688184	347	18.73494258	0.008731988
201710_at	MYBL2	-1.257884972	348	18.72255955	0.008735632
40612_at	DOPEY1	1.306765254	349	18.71256481	0.008767908
204128_s_at	RFC3	-1.260503392	350	18.71221335	0.008742857
223892_s_at	TMBIM4	1.183451022	351	18.70776698	0.008774929
212036_s_at	PNN	-1.124278924	352	18.70186349	0.00875

203773_x_at	BLVRA	1.528376521	353	18.69646256	0.008753541
203097_s_at	RAPGEF2	1.745935182	354	18.69345157	0.008728814
216899_s_at	SKAP2	1.4063932	355	18.68624513	0.008704225
217974_at	TM7SF3	1.43097652	356	18.67998764	0.008707865
243521_at	AW590862	1.16634937	357	18.67009104	0.008683473
222620_s_at	DNAJC1	1.285206337	358	18.65275777	0.008715084
201972_at	ATP6V1A	1.264879542	359	18.63521674	0.008857939
216251_s_at	TTLL12	-1.28877463	360	18.62689754	0.008916667
201311_s_at	SH3BGRL	1.159095952	361	18.62460875	0.008891967
226122_at	PLEKHG1	1.715941061	362	18.61963794	0.008922652
226262_at	AA534526	-1.223488041	363	18.60173283	0.008898072
228478_at	AA889954	1.271031689	364	18.59215069	0.008873626
207098_s_at	MFN1	1.370782805	365	18.57051901	0.008931507
225202_at	RHOBTB3	2.421666168	366	18.56711421	0.008961749
201999_s_at	DYNLT1	1.356604327	367	18.55892699	0.008991826
224696_s_at	WDR22	1.139183377	368	18.52647375	0.009103261
221746_at	UBL4A	-1.146312186	369	18.5212777	0.009078591
207339_s_at	LTB	-2.153972752	370	18.51205551	0.009135135
224468_s_at	C19orf48	-1.197478705	371	18.46907183	0.00916442
235812_at	C16orf69	1.154285418	372	18.45574572	0.009166667
221918_at	PCTK2	1.355664327	373	18.42806489	0.009276139
206055_s_at	SNRPA1	-1.176906737	374	18.41777844	0.009304813
202078_at	COPS3	-1.109569472	375	18.41231945	0.00928
229350_x_at	PARP10	1.237132479	376	18.32679747	0.009574468
222401_s_at	TMEM50A	1.143930973	377	18.31809476	0.009549072
226440_at	DUSP22	1.292352831	378	18.29806551	0.009603175
244422_at	AI494573	-1.424050196	379	18.28941094	0.009577836
236266_at	LOC283666	2.388326374	380	18.28035911	0.009552632
208700_s_at	TKT	-1.194991205	381	18.27728823	0.009553806
203955_at	KIAA0649	1.387030969	382	18.26965586	0.009581152
215930_s_at	CTAGE5	1.325007017	383	18.25455076	0.009634465
207621_s_at	PEMT	-1.138394029	384	18.2412241	0.009635417
210250_x_at	ADSL	-1.167967395	385	18.24039054	0.00961039
222914_s_at	TMEM121	-1.151089491	386	18.23996872	0.009585492
202534_x_at	DHFR	-1.300440147	387	18.22883146	0.009638243
214114_x_at	FASTK	1.125058485	388	18.20812408	0.009690722
209004_s_at	FBXL5	1.214194884	389	18.20354838	0.00966581
222845_x_at	TMBIM4	1.167967395	390	18.18686271	0.009692308
1555464_at	IFIH1	1.365093718	391	18.15879678	0.009744246
201075_s_at	SMARCC1	-1.244874235	392	18.15096746	0.009719388
226333_at	AV700030	-2.289448321	393	18.13919279	0.009745547
221511_x_at	CCPG1	1.695839929	394	18.13685941	0.009720812
218671_s_at	ATPIF1	-1.118061851	395	18.13262328	0.009696203
209418_s_at	THOC5	-1.095811766	396	18.13028055	0.009671717
223178_s_at	NT5DC1	1.264003098	397	18.10434955	0.009748111
231690_at	AI962352	1.45195828	398	18.07800654	0.009773869
225458_at	LOC25845	-1.20664392	399	18.06718127	0.009749373
218161_s_at	CLN6	-1.260503392	400	18.05322556	0.0098
209900_s_at	SLC16A1	-1.232852325	401	18.04478882	0.009875312
203732_at	TRIP4	1.163120042	402	18.04369278	0.009850746
202911_at	MSH6	-1.199971382	403	18.04229553	0.009826303

218581_at	ABHD4	1.33422317	404	18.04116719	0.00980198
238190_at	TUFM	-1.115739322	405	18.03868592	0.009802469
209194_at	CETN2	1.136816973	406	18.03048097	0.009778325
221788_at	PGM3	1.23370717	407	18.01323065	0.00980344
227056_at	KIAA0141	1.260503392	408	18.00179268	0.009803922
222156_x_at	CCPG1	1.793776319	409	17.98866449	0.009828851
227802_at	AI075999	1.693490625	410	17.96476059	0.009829268
201968_s_at	PGM1	1.209994089	411	17.95548608	0.009854015
226809_at	FLJ30428	-1.577893682	412	17.92872184	0.009951456
203194_s_at	NUP98	-1.17609125	413	17.92591333	0.009927361
212150_at	KIAA0143	1.195819797	414	17.91918071	0.009951691
213134_x_at	BTG3	1.257013375	415	17.91156051	0.009927711
238695_s_at	RAB39B	1.276328769	416	17.91126502	0.009903846
1559220_at	BG025779	-1.144724161	417	17.90520689	0.009880096
228763_at	CHMP4A	1.182631	418	17.90290783	0.009856459
223217_s_at	NFKBIZ	1.64832417	419	17.89830127	0.009856802
200977_s_at	TAX1BP1	1.270150983	420	17.87151735	0.00997619
201312_s_at	SH3BGRL	1.163120042	421	17.84377618	0.010118765
201276_at	RAB5B	1.286097483	422	17.81288128	0.010165877
217783_s_at	YPEL5	1.576800348	423	17.74178732	0.010307329
212378_at	GART	-1.163120042	424	17.71925349	0.010400943
221521_s_at	GINS2	-1.29145735	425	17.70468751	0.010423529
41037_at	TEAD4	-2.179504224	426	17.66096316	0.010539906
225484_at	TSGA14	-1.258757174	427	17.659537	0.01058548
210907_s_at	PDCD10	1.110338834	428	17.65533541	0.010560748
226941_at	BF439325	1.28788163	429	17.65366849	0.010536131
234749_s_at	WDR51A	-1.187559666	430	17.65224787	0.010511628
208671_at	SERINC1	1.204972315	431	17.63858239	0.010487239
203303_at	DYNLT3	1.38991822	432	17.62485713	0.010486111
1552257_a_at	TTLL12	-1.236275261	433	17.62031941	0.010461894
205632_s_at	PIP5K1B	1.571345033	434	17.57193466	0.01062212
209457_at	DUSP5	1.528376521	435	17.55676123	0.010689655
203832_at	SNRPF	-1.122721422	436	17.54054944	0.010665138
209974_s_at	BUB3	-1.123499903	437	17.52456065	0.010709382
218456_at	C1QDC1	1.350974085	438	17.5088239	0.010799087
211790_s_at	MLL2	-1.126619228	439	17.50233069	0.010774487
224561_s_at	MORF4L1	1.107264584	440	17.50200809	0.01075
215548_s_at	SCFD1	1.134455485	441	17.48459234	0.010816327
211593_s_at	MAST2	-1.217566019	442	17.47705864	0.010791855
223506_at	ZC3H8	-1.155085785	443	17.44939294	0.010902935
232800_at	LOC730943	1.461044379	444	17.42650206	0.011013514
224720_at	MIB1	1.293248932	445	17.42637128	0.010988764
227423_at	LRRC28	1.292352831	446	17.4228293	0.010964126
218564_at	RFWD3	-1.277213759	447	17.39308861	0.011006711
217598_at	CINP	1.257884972	448	17.39303367	0.010982143
225076_s_at	ZNFX1	1.322254605	449	17.34642907	0.011180401
204853_at	ORC2L	-1.172022284	450	17.33732731	0.011244444
226268_at	RAB21	1.231998073	451	17.32983249	0.011286031
218116_at	C9orf78	-1.10343374	452	17.32045115	0.011261062
201479_at	DKC1	-1.146312186	453	17.3154306	0.011346578
202777_at	SHOC2	1.176906737	454	17.27989443	0.011409692

233842_x_at	C20orf43	1.096571589	455	17.27049216	0.011384615
212694_s_at	PCCB	-1.231998073	456	17.25241723	0.011403509
219435_at	C17orf68	1.147902414	457	17.23496991	0.011466083
223834_at	CD274	1.788809804	458	17.22850774	0.011528384
215933_s_at	HHEX	-2.318191904	459	17.22065382	0.011503268
217759_at	TRIM44	1.167158102	460	17.20335348	0.011608696
226195_at	C14orf179	1.283425898	461	17.20089964	0.011583514
201384_s_at	NBR1	1.218410264	462	17.19101444	0.011645022
230803_s_at	ARHGAP24	1.504203751	463	17.18123245	0.01161987
201412_at	LRP10	1.20664392	464	17.1688648	0.011702586
225541_at	RPL22L1	-1.099616149	465	17.15788394	0.011698925
214205_x_at	TXNL2	-1.110338834	466	17.15523409	0.01167382
218073_s_at	TMEM48	-1.309485423	467	17.15158359	0.011648822
227458_at	AI608902	1.859609885	468	17.13221332	0.011688034
219317_at	POLI	1.424050196	469	17.12115364	0.011663113
220140_s_at	SNX11	1.403471726	470	17.1145288	0.011723404
205812_s_at	TMED9	1.212512819	471	17.11277079	0.011698514
207480_s_at	MEIS2	-2.334316204	472	17.11031943	0.011673729
224884_at	AKAP13	1.242288282	473	17.10871828	0.011691332
204890_s_at	LCK	-1.466116757	474	17.10536441	0.011666667
226295_at	ITFG2	1.156688184	475	17.10448518	0.011642105
220022_at	ZNF334	1.73868912	476	17.08136354	0.011764706
228049_x_at	AA523172	-1.395710764	477	17.07859252	0.011761006
222478_at	VPS36	1.21335356	478	17.07061717	0.011736402
217007_s_at	ADAM15	-1.209994089	479	17.06147989	0.0117119
206833_s_at	ACYP2	1.294145654	480	17.02985027	0.011875
217982_s_at	MORF4L1	1.118061851	481	17.0250842	0.011850312
210426_x_at	RORA	2.615342697	482	17.00159516	0.011950207
222516_at	AP3M1	1.10343374	483	16.97449831	0.012049689
209417_s_at	IFI35	1.441928871	484	16.96952347	0.012045455
231899_at	ZC3H12C	1.492778383	485	16.96677919	0.012020619
214794_at	DLST	-1.300440147	486	16.96257639	0.011995885
217884_at	NAT10	-1.153485605	487	16.95147403	0.011991786
204798_at	MYB	-1.522033381	488	16.9473698	0.012008197
226733_at	PFKFB2	1.403471726	489	16.93354391	0.01208589
217466_x_at	RPS2	-1.038139271	490	16.93038046	0.012061224
230100_x_at	PAK1	1.547564994	491	16.92864518	0.01209776
1729_at	TRADD	1.327765158	492	16.92307063	0.012073171
209151_x_at	CTNND2	-1.625631204	493	16.88695451	0.012494929
202741_at	PRKACB	1.631274987	494	16.88549947	0.012469636
215714_s_at	SMARCA4	-1.356604327	495	16.88136155	0.012444444
210479_s_at	RORA	2.804998501	496	16.8614705	0.012520161
219237_s_at	DNAJB14	1.231144413	497	16.8077351	0.012857143
203195_s_at	NUP98	-1.085229372	498	16.80019406	0.012831325
1559946_s_at	RUVBL2	-1.112650121	499	16.79473914	0.012865731
201738_at	EIF1B	1.141554707	500	16.77548496	0.01294
229595_at	CHCHD4	-1.175276328	501	16.76578932	0.013013972
208094_s_at	CCDC130	1.150291893	502	16.7579265	0.013067729
55616_at	PERLD1	1.110338834	503	16.7533599	0.01304175
206052_s_at	SLBP	-1.162314108	504	16.73981134	0.013075397
201628_s_at	RRAGA	1.118837101	505	16.7395143	0.013049505

225000_at	PRKAR2A	-1.162314108	506	16.73390534	0.013023715
234672_s_at	TMEM48	-1.278985581	507	16.73037351	0.012998028
211153_s_at	TNFSF11	-1.986184991	508	16.72764239	0.012992126
226170_at	AA151838	-1.191682575	509	16.72693856	0.012966601
201336_at	VAMP3	1.231144413	510	16.72670107	0.012941176
210001_s_at	SOCS1	1.62788637	511	16.72507252	0.012915851
218446_s_at	FAM18B	1.205807828	512	16.71854453	0.012910156
218528_s_at	RNF38	1.147902414	513	16.71415153	0.01288499
218845_at	DUSP22	1.286989247	514	16.71116436	0.012859922
228363_at	BIRC4	1.256142381	515	16.70069385	0.012873786
212279_at	TMEM97	-1.360370852	516	16.68655365	0.012945736
236160_at	AA765470	1.159899655	517	16.68534194	0.012920696
229784_at	MGC16121	-1.32408891	518	16.65188983	0.012992278
208699_x_at	TKT	-1.244011653	519	16.63822207	0.013082852
201692_at	OPRS1	-1.742308384	520	16.63721926	0.013057692
240281_at	RFT1	-1.100378609	521	16.63615008	0.01303263
207169_x_at	DDR1	1.345367209	522	16.60576475	0.013045977
225227_at	AW294869	1.409320755	523	16.58625991	0.013154876
212058_at	SR140	-1.133669413	524	16.5842462	0.013129771
221652_s_at	C12orf11	-1.120389214	525	16.58380937	0.013104762
203179_at	GALT	-1.175276328	526	16.58156403	0.013079848
218942_at	PIP5K2C	1.221793102	527	16.53868351	0.013244782
202191_s_at	GAS7	1.74956953	528	16.5281536	0.013257576
204205_at	APOBEC3G	1.271913007	529	16.52276631	0.013232514
202027_at	C22orf5	1.372684431	530	16.52252962	0.013207547
220089_at	L2HGDH	-1.272794935	531	16.51338537	0.013258004
211956_s_at	EIF1	1.062895674	532	16.49880377	0.013327068
224986_s_at	PDPK1	1.117287138	533	16.48782322	0.013320826
212281_s_at	TMEM97	-1.29145735	534	16.46517656	0.01340824
204118_at	CD48	1.219255094	535	16.4546078	0.013383178
212130_x_at	EIF1	1.175276328	536	16.45399042	0.013358209
222212_s_at	LASS2	-1.232852325	537	16.44692998	0.013351955
242997_at	AW664311	-1.121166078	538	16.44375413	0.013327138
205548_s_at	BTG3	1.257013375	539	16.44153797	0.013302412
212218_s_at	FASN	-1.563739286	540	16.4406518	0.013277778
222444_at	ARMCX3	1.347233577	541	16.43478857	0.013327172
201413_at	HSD17B4	1.17772279	542	16.41303277	0.013413284
218571_s_at	CHMP4A	1.145517898	543	16.41288337	0.013388582
1552287_s_at	AFG3L1	-1.165541198	544	16.39229642	0.013455882
205024_s_at	RAD51	-1.181811547	545	16.38917337	0.013504587
218477_at	TMEM14A	1.394743666	546	16.3697687	0.013608059
217978_s_at	UBE2Q1	1.064370182	547	16.35814246	0.013656307
226507_at	PAK1	1.371733289	548	16.34392085	0.013667883
209354_at	TNFRSF14	1.284315809	549	16.34026478	0.013642987
225433_at	GTF2A1	1.181811547	550	16.33134522	0.013654545
218215_s_at	NR1H2	1.147902414	551	16.32775462	0.013647913
209574_s_at	C18orf1	1.511519928	552	16.3216262	0.013623188
203530_s_at	STX4	1.172834949	553	16.31015032	0.013652803
1555037_a_at	IDH1	1.224336392	554	16.30348989	0.013646209
1552279_a_at	PCFT	-1.128964405	555	16.29948405	0.013675676
214857_at	C10orf95	-1.143930973	556	16.29897588	0.013651079

205393_s_at	CHEK1	-1.215036792	557	16.29483702	0.013626571
204546_at	KIAA0513	1.417157397	558	16.29380595	0.013602151
206011_at	CASP1	3.226567037	559	16.27267537	0.013685152
204045_at	TCEAL1	1.151887642	560	16.24459737	0.013767857
214484_s_at	OPRS1	-1.793776319	561	16.24068578	0.013743316
217750_s_at	UBE2Z	1.134455485	562	16.21207545	0.013914591
211138_s_at	KMO	1.445932295	563	16.19948394	0.01401421
212305_s_at	MIA3	1.176906737	564	16.19053023	0.013989362
203593_at	CD2AP	1.195819797	565	16.18105749	0.014123894
219972_s_at	C14orf135	1.23370717	566	16.1618771	0.014204947
206566_at	SLC7A1	-1.327765158	567	16.11965969	0.014585538
213293_s_at	TRIM22	1.28877463	568	16.11891527	0.014559859
225402_at	TP53RK	-1.135242102	569	16.10937564	0.014586995
201076_at	NHP2L1	-1.202469249	570	16.10575596	0.014561404
205540_s_at	RRAGB	1.151887642	571	16.10481406	0.014535902
212908_at	DNAJC16	1.149494848	572	16.08641614	0.01458042
205811_at	POLG2	-1.160703914	573	16.08304681	0.014554974
222946_s_at	C1orf135	-1.260503392	574	16.08211627	0.014529617
216551_x_at	PLCG1	-1.164733586	575	16.06459213	0.014643478
223564_s_at	GNB1L	-1.171210181	576	16.05982317	0.014618056
227031_at	AV681975	1.279872414	577	16.05574408	0.014592721
204610_s_at	CCDC85B	-1.309485423	578	16.05341436	0.014567474
223501_at	AW151360	1.527317498	579	16.0483717	0.014594128
204554_at	PPP1R3D	1.208317843	580	16.03183096	0.014586207
221790_s_at	LDLRAP1	-1.440929749	581	16.00689293	0.014767642
213939_s_at	RUFY3	1.80000386	582	15.98977054	0.014845361
206042_x_at	SNRPN	1.219255094	583	15.98210552	0.014819897
203785_s_at	DDX28	-1.183451022	584	15.98146719	0.014794521
218550_s_at	LRRC20	-1.109569472	585	15.98129334	0.014769231
212227_x_at	EIF1	1.167158102	586	15.97172614	0.014829352
201859_at	PRG1	1.139183377	587	15.9699701	0.01483816
238992_at	POLI	1.381274448	588	15.96820254	0.014812925
214150_x_at	ATP6V0E1	1.167158102	589	15.96670595	0.014787776
201776_s_at	KIAA0494	1.138394029	590	15.95853459	0.014830508
48808_at	DHFR	-1.325007017	591	15.94006837	0.014856176
218803_at	CHFR	1.22010051	592	15.92567615	0.015
222621_at	DNAJC1	1.246601194	593	15.92528008	0.014974705
1555865_at	LOC255512	-1.167967395	594	15.91570453	0.015016835
210101_x_at	SH3GLB1	1.175276328	595	15.91397119	0.014991597
226178_at	SOCS4	1.125058485	596	15.91228232	0.014966443
226091_s_at	MRFAP1	1.143930973	597	15.90078533	0.014941374
232965_at	LOC400684	-1.173648178	598	15.89602219	0.01493311
227905_s_at	AZI2	1.155085785	599	15.89262523	0.01490818
1556601_a_at	SPATA13	1.320422841	600	15.89171798	0.014883333
231013_at	W80446	1.264879542	601	15.88905645	0.014908486
203612_at	BYSL	-1.161508732	602	15.88819964	0.014883721
225483_at	VPS26B	-1.223488041	603	15.88458097	0.014859038
202897_at	SIRPA	1.45195828	604	15.86907872	0.014900662
206499_s_at	RCC1	-1.396678532	605	15.85869544	0.014975207
202021_x_at	EIF1	1.167967395	606	15.85573034	0.014950495
203555_at	PTPN18	-1.248330549	607	15.81952325	0.015074135

235507_at	PCMTD1	1.284315809	608	15.81884914	0.015049342
221825_at	ANGEL2	1.136816973	609	15.8149741	0.015024631
212786_at	KIAA0350	1.245737416	610	15.80776843	0.015098361
1555882_at	SPIN3	1.517819253	611	15.80089528	0.01507365
48580_at	CXXC1	-1.22858698	612	15.79241258	0.015081699
204004_at	PAWR	-1.486582984	613	15.79237909	0.015057096
242136_x_at	MGC70870	1.720705261	614	15.79158112	0.015032573
207819_s_at	ABCB4	1.747145792	615	15.78667047	0.015073171
219123_at	ZNF232	-1.232852325	616	15.77371457	0.015146104
1554261_at	KBTBD9	-1.167967395	617	15.75524591	0.015299838
222751_at	HERPUD2	1.289668251	618	15.73861848	0.01538835
222488_s_at	DCTN4	1.199971382	619	15.70526515	0.015476575
202796_at	SYNPO	1.488645255	620	15.70414085	0.015451613
209009_at	ESD	-1.119612889	621	15.70000353	0.015426731
201074_at	SMARCC1	-1.144724161	622	15.68977089	0.015498392
225849_s_at	SFT2D1	1.189207115	623	15.68310449	0.015473515
204439_at	IFI44L	1.596596773	624	15.6826449	0.015448718
208898_at	ATP6V1D	1.149494848	625	15.66763627	0.015504
239973_at	AW450675	-1.572434584	626	15.65081204	0.015527157
219998_at	HSPC159	-1.183451022	627	15.6485422	0.01553429
209150_s_at	TM9SF1	1.171210181	628	15.63404098	0.015541401
1560316_s_at	GLCCI1	1.366987452	629	15.62517259	0.01554849
221641_s_at	ACOT9	1.308578071	630	15.60937031	0.015730159
209958_s_at	PTHB1	1.171210181	631	15.60063371	0.01570523
201098_at	COPB2	1.113421618	632	15.59916711	0.01568038
202317_s_at	UBE4B	1.093535457	633	15.59294559	0.015655608
225859_at	BIRC4	1.221793102	634	15.58583726	0.015709779
231858_x_at	DKFZp761E198	-1.152686347	635	15.5854549	0.015685039
228910_at	AI870617	1.43097652	636	15.58517587	0.015660377
200875_s_at	NOL5A	-1.21335356	637	15.58477951	0.015635793
203429_s_at	C1orf9	1.243149669	638	15.5830828	0.015611285
236250_at	ST6GALNAC2	-1.210833084	639	15.57363844	0.015665102
203733_at	DEXI	1.298638603	640	15.56639483	0.015703125
238566_at	BF592775	1.397646972	641	15.55894434	0.015709828
210975_x_at	FASTK	1.118061851	642	15.55667313	0.015685358
226390_at	STARD4	1.307671349	643	15.54944004	0.015769829
218999_at	TMEM140	1.720705261	644	15.54926445	0.015745342
214179_s_at	NFE2L1	1.188383105	645	15.50120373	0.015984496
208869_s_at	GABARAPL1	1.52414483	646	15.50003643	0.015959752
211254_x_at	RHAG	-1.106497353	647	15.49787708	0.015981453
225642_at	KTI12	1.217566019	648	15.49681096	0.01595679
202641_at	ARL3	1.168777249	649	15.48974661	0.01596302
200839_s_at	CTSB	1.4054187	650	15.48682237	0.015938462
203103_s_at	PRPF19	-1.269270886	651	15.48676415	0.015913978
203701_s_at	TRMT1	-1.165541198	652	15.48620686	0.015889571
234926_s_at	C20orf43	1.095052471	653	15.47878738	0.015926493
AFFX-HUMISGI	STAT1	1.25353302	654	15.47222096	0.015902141
228359_at	STS-1	-2.770218936	655	15.45072669	0.016015267
218004_at	BSDC1	1.165541198	656	15.43895821	0.016067073
210463_x_at	TRMT1	-1.174461971	657	15.43824621	0.016042618
204160_s_at	ENPP4	1.697015803	658	15.43601897	0.016018237

205945_at	IL6R	-2.793357065	659	15.42465481	0.016009105
217445_s_at	GART	-1.289668251	660	15.40479298	0.016045455
224644_at	AV724183	1.136816973	661	15.39960518	0.016051437
211951_at	NOLC1	-1.143138335	662	15.36792376	0.016193353
1554670_at	GGA1	-1.249196126	663	15.35516075	0.016229261
200814_at	PSME1	1.127400412	664	15.3548443	0.016204819
229236_s_at	SFXN4	-1.225185332	665	15.35142128	0.016180451
204197_s_at	RUNX3	1.245737416	666	15.35083608	0.016156156
217249_x_at	AC004544	1.095052471	667	15.34833612	0.016206897
218575_at	ANAPC1	-1.090507733	668	15.30980471	0.016467066
221030_s_at	ARHGAP24	1.241427492	669	15.30521675	0.016442451
208628_s_at	YBX1	-1.059218335	670	15.30357515	0.01641791
208794_s_at	SMARCA4	-1.215879283	671	15.2967788	0.016453055
202676_x_at	FASTK	1.125838586	672	15.28469562	0.016517857
201555_at	MCM3	-1.21335356	673	15.27435614	0.016627043
223414_s_at	LYAR	-1.237990291	674	15.27360162	0.016602374
1554827_a_at	ADCY7	-1.22858698	675	15.26885858	0.016711111
1569368_at	LOC648921	-1.257884972	676	15.26052848	0.016686391
201828_x_at	FAM127A	1.256142381	677	15.25769849	0.016735598
200887_s_at	STAT1	1.207480591	678	15.2565246	0.016710914
213733_at	MYO1F	-2.102345818	679	15.25310024	0.016686303
241948_at	FAM122A	1.481439798	680	15.24706722	0.016705882
226370_at	KLHL15	1.219255094	681	15.24287249	0.016681351
212443_at	NBEAL2	-1.586667686	682	15.21766382	0.016832845
230060_at	CDCA7	-1.361314116	683	15.2024893	0.016866764
215884_s_at	UBQLN2	1.124278924	684	15.19800649	0.016944444
208786_s_at	MAP1LC3B	1.244011653	685	15.18949729	0.016948905
218634_at	PHLDA3	1.638073396	686	15.18735911	0.016924198
1559064_at	NUP153	-1.159899655	687	15.16976671	0.017045124
211851_x_at	BRCA1	-1.200803427	688	15.16951166	0.017020349
215983_s_at	UBXD6	-1.190031696	689	15.15646335	0.01701016
228415_at	AA205444	-1.435944511	690	15.14951198	0.017
1556151_at	ITFG1	1.227735684	691	15.14827059	0.016975398
230442_at	MTHFSD	-1.235418637	692	15.14360483	0.016950867
204630_s_at	GOSR1	1.129747215	693	15.14100912	0.016926407
212411_at	IMP4	-1.246601194	694	15.13916806	0.016945245
203225_s_at	RFK	-1.311302014	695	15.11864838	0.017064748
228314_at	BE877357	2.09216988	696	15.1117885	0.01704023
218664_at	MECR	-1.139183377	697	15.07365446	0.017259684
211576_s_at	SLC19A1	-1.321338406	698	15.06440272	0.017292264
227674_at	ZNF585A	1.167967395	699	15.05934487	0.017353362
243109_at	MCTP2	2.842184086	700	15.04313116	0.0174
202211_at	ARFGAP3	1.219255094	701	15.03855273	0.01746077
209336_at	PWP2	-1.16634937	702	15.03642282	0.017435897
210749_x_at	DDR1	1.295042999	703	15.03589273	0.017411095
207128_s_at	ZNF223	1.424050196	704	15.03309379	0.017386364
1555522_s_at	C2orf4	-1.121943481	705	15.02323694	0.01741844
211639_x_at	C12orf32	2.854029011	706	15.02237714	0.017393768
217299_s_at	NBN	1.245737416	707	15.02105336	0.017369165
235055_x_at	MUC4	-1.168777249	708	15.00837453	0.017514124
218763_at	STX18	1.151089491	709	15.00723945	0.017489422

208793_x_at	SMARCA4	-1.391846392	710	15.00711311	0.017464789
212088_at	PMPCA	-1.132098902	711	14.99592693	0.017538678
201172_x_at	ATP6V0E1	1.155886707	712	14.9895714	0.01755618
219258_at	TIPIN	-1.16634937	713	14.97021828	0.017587658
217839_at	TFG	1.154285418	714	14.96817488	0.017661064
209927_s_at	C1orf77	-1.119612889	715	14.96434192	0.017636364
204836_at	GLDC	-1.62113024	716	14.95798756	0.017667598
230075_at	RAB39B	1.315854525	717	14.95200001	0.017642957
222386_s_at	COPZ1	1.138394029	718	14.94294863	0.017660167
205512_s_at	AIFM1	-1.139973273	719	14.93475058	0.017732962
214728_x_at	SMARCA4	-1.209155676	720	14.93362853	0.017708333
211197_s_at	ICOSLG	-1.235418637	721	14.93018485	0.017683773
218614_at	C12orf35	-1.235418637	722	14.9236176	0.017700831
218191_s_at	LMBRD1	1.282536603	723	14.91122442	0.017828492
212380_at	KIAA0082	1.130530567	724	14.89055847	0.017955801
225722_at	FAM78B	-1.180992661	725	14.88489538	0.017972414
226159_at	LOC285636	1.129747215	726	14.87575231	0.018002755
208858_s_at	FAM62A	-1.248330549	727	14.87408039	0.017977992
219484_at	HCFC2	1.159095952	728	14.8676222	0.018049451
235962_at	AI336192	1.126619228	729	14.86212264	0.018024691
215747_s_at	RCC1	-1.446934886	730	14.84425455	0.018109589
212342_at	YIPF6	1.162314108	731	14.84084101	0.018084815
223502_s_at	TNFSF13B	1.53049677	732	14.82611745	0.018128415
209075_s_at	ISCU	1.359428242	733	14.81179341	0.018199181
229287_at	BE326214	1.352848231	734	14.80536335	0.018201635
219543_at	PBLD	1.316766922	735	14.80428342	0.018176871
211661_x_at	PTAFR	-2.228387302	736	14.79090387	0.018206522
201456_s_at	BUB3	-1.16634937	737	14.78035456	0.018222524
222464_s_at	C10orf119	-1.20664392	738	14.77716897	0.018292683
217542_at	CPM	1.402499251	739	14.77418618	0.01826793
224015_s_at	MRPS25	-1.110338834	740	14.74457897	0.018459459
203275_at	IRF2	1.149494848	741	14.73538088	0.018529015
213021_at	GOSR1	1.132098902	742	14.72922478	0.018584906
206278_at	PTAFR	-2.005552872	743	14.72074435	0.018559892
202431_s_at	MYC	-1.365093718	744	14.71796822	0.018629032
205306_x_at	KMO	1.486582984	745	14.71792284	0.018604027
217664_at	JMJD2B	-1.162314108	746	14.71607906	0.018579088
227856_at	C4orf32	1.434949535	747	14.71570532	0.018554217
203158_s_at	GLS	1.333298677	748	14.70640594	0.018596257
204267_x_at	PKMYT1	-1.167967395	749	14.69770718	0.018664887
227268_at	LOC51136	-1.339783602	750	14.69527639	0.01864
230492_s_at	RP5-1022P6.2	1.277213759	751	14.67597964	0.01873502
202076_at	BIRC2	1.30224419	752	14.67498548	0.018710106
208206_s_at	RASGRP2	-1.514666316	753	14.66941728	0.018818061
201236_s_at	BTG2	1.293248932	754	14.664334	0.018793103
200657_at	SLC25A5	-1.07549439	755	14.65499147	0.018807947
217809_at	BZW2	-1.788809804	756	14.64301617	0.018835979
1559705_s_at	PHKA2	-1.115739322	757	14.6355539	0.018863937
222477_s_at	TM7SF3	1.232852325	758	14.63331246	0.01883905
205687_at	UBPH	1.147107024	759	14.6322324	0.018814229
203480_s_at	OTUD4	1.273677475	760	14.63170117	0.018789474

204891_s_at	LCK	-1.427014506	761	14.59712993	0.018961892
204781_s_at	FAS	1.358486285	762	14.58811677	0.019002625
233893_s_at	KIAA1530	1.247465572	763	14.57300291	0.019003932
201188_s_at	ITPR3	-1.256142381	764	14.56065692	0.019070681
201478_s_at	DKC1	-1.188383105	765	14.54482834	0.019189542
221474_at	MRLC2	1.114193651	766	14.54012939	0.019164491
203584_at	TTC35	1.158292806	767	14.51800854	0.019322034
224945_at	BTBD7	1.186736798	768	14.51227821	0.019296875
224872_at	DIP2B	1.209155676	769	14.50469913	0.019362809
229267_at	ANAPC1	-1.135242102	770	14.50404207	0.019337662
212641_at	HIVEP2	1.426025717	771	14.49811353	0.019351492
222420_s_at	UBE2H	1.20664392	772	14.49649784	0.019326425
209626_s_at	OSBPL3	1.32592576	773	14.49393752	0.019301423
212430_at	RBM38	-1.380317353	774	14.49231117	0.019276486
219849_at	ZNF671	1.164733586	775	14.49064542	0.019251613
200743_s_at	TPP1	1.241427492	776	14.48876259	0.019342784
209778_at	TRIP11	1.169587664	777	14.48769763	0.019317889
224637_at	BF211019	1.130530567	778	14.48552914	0.019293059
216231_s_at	B2M	1.056285625	779	14.47413675	0.019370988
228607_at	OAS2	1.425037614	780	14.46196615	0.019461538
219371_s_at	KLF2	-2.458877735	781	14.46050259	0.01943662
220750_s_at	LEPRE1	1.114966219	782	14.45920824	0.019514066
238423_at	SYTL3	1.689972769	783	14.44287717	0.019527458
202475_at	TMEM147	-1.237132479	784	14.43566297	0.019566327
231968_at	AK025416	1.151089491	785	14.43250488	0.019541401
1554690_a_at	TACC1	1.246601194	786	14.42336863	0.019554707
201858_s_at	PRG1	1.29056249	787	14.42243905	0.01952986
208921_s_at	SRI	1.258757174	788	14.41082998	0.019543147
206592_s_at	AP3D1	1.157490217	789	14.4057257	0.019594423
209302_at	POLR2H	-1.119612889	790	14.39431349	0.019670886
203755_at	BUB1B	-1.189207115	791	14.37088202	0.019898862
226191_at	AI431788	1.136816973	792	14.36381641	0.02
218500_at	C8orf55	-1.273677475	793	14.35878003	0.020025221
222838_at	SLAMF7	1.426025717	794	14.35421496	0.02
235567_at	LOC283666	1.606587994	795	14.35242646	0.019974843
224787_s_at	RAB18	1.129747215	796	14.3450512	0.020062814
218146_at	GLT8D1	1.32592576	797	14.34357562	0.020037641
227152_at	C12orf35	-1.30224419	798	14.33934037	0.020213033
209357_at	CITED2	1.557249382	799	14.33692726	0.020187735
205070_at	ING3	1.193335743	800	14.3344166	0.0201625
226353_at	SPPL2A	1.215036792	801	14.32761711	0.020249688
200838_at	CTSB	1.368883813	802	14.32544282	0.020224439
232735_at	ANKRD34	-1.200803427	803	14.32185465	0.020199253
225680_at	DKFZp434K181	-1.204972315	804	14.31502388	0.020223881
218815_s_at	TMEM51	2.020902893	805	14.31356772	0.020198758
202843_at	DNAJB9	1.657489809	806	14.30188116	0.020260546
202265_at	BMI1	1.351910833	807	14.30024763	0.02023544
224511_s_at	TXNL5	-1.122721422	808	14.29676565	0.020309406
228438_at	TRPA1	1.335148303	809	14.29048234	0.020284302
213430_at	RUFY3	1.277213759	810	14.27039577	0.02045679
213527_s_at	ZNF688	1.219255094	811	14.26697791	0.020468557

202932_at	YES1	1.397646972	812	14.2656753	0.02044335
218819_at	INTS6	1.138394029	813	14.25808013	0.020516605
239891_x_at	RAB12	1.620006947	814	14.24683883	0.020577396
213315_x_at	CXorf40A	1.136029265	815	14.23468384	0.020613497
208117_s_at	LAS1L	-1.091263877	816	14.22281727	0.02067402
201459_at	RUVBL2	-1.121166078	817	14.21422798	0.020648715
219691_at	SAMD9	1.344434994	818	14.20046791	0.020672372
204960_at	PTPRCAP	-1.425037614	819	14.20002041	0.020647131
224428_s_at	CDCA7	-1.556170353	820	14.19733857	0.020707317
225629_s_at	ZBTB4	1.283425898	821	14.19438595	0.020682095
202787_s_at	MAPKAPK3	-1.361314116	822	14.19131606	0.020656934
212209_at	THRAP2	-1.888184838	823	14.19125456	0.020631835
235222_x_at	BIRC4	1.194991205	824	14.18661416	0.020667476
224819_at	TCEAL8	1.155085785	825	14.18586595	0.020642424
225460_at	SEC22C	1.149494848	826	14.18566002	0.020617433
242406_at	C1orf186	1.613283518	827	14.17987135	0.020737606
209300_s_at	NECAP1	1.132883885	828	14.17523977	0.02071256
219618_at	IRAK4	1.114193651	829	14.17419699	0.020687575
220011_at	C1orf135	-1.240567298	830	14.17122286	0.020662651
204777_s_at	MAL	1.919858522	831	14.16910458	0.020722022
219004_s_at	C21orf45	-1.183451022	832	14.16308159	0.020697115
209519_at	NCBP1	-1.373636233	833	14.16257445	0.020672269
217929_s_at	KIAA0319L	1.183451022	834	14.15917983	0.020707434
226328_at	KLF16	-1.203303026	835	14.1518438	0.020682635
219248_at	THUMPD2	-1.175276328	836	14.14638747	0.020777512
209627_s_at	OSBPL3	1.35754498	837	14.13350597	0.020812425
201553_s_at	LAMP1	1.187559666	838	14.12026715	0.020835322
201912_s_at	GSPT1	-1.167158102	839	14.11204247	0.020870083
211450_s_at	MSH6	-1.312211255	840	14.10038905	0.020928571
209573_s_at	C18orf1	1.339783602	841	14.09794176	0.021034483
235103_at	MAN2A1	1.964279191	842	14.09610295	0.021009501
228042_at	ADPRH	1.278985581	843	14.09327886	0.020984579
204862_s_at	NME3	1.209994089	844	14.09231321	0.020959716
220661_s_at	ZNF692	-1.144724161	845	14.08628784	0.021029586
228737_at	C20orf100	-1.986184991	846	14.08168996	0.021004728
227216_at	RLTPR	-1.328685814	847	14.07911258	0.021086187
238792_at	PCNX	1.327765158	848	14.06536384	0.021108491
230281_at	C16orf46	-1.138394029	849	14.04874913	0.021213192
218147_s_at	GLT8D1	1.385109468	850	14.04609994	0.021188235
223684_s_at	SMUG1	1.108800644	851	14.04608306	0.021163337
232472_at	FNDC3B	1.42800398	852	14.04293691	0.021138498
244631_at	LOC389834	2.556198726	853	14.03619186	0.021184056
200980_s_at	PDHA1	-1.112650121	854	14.02531395	0.021241218
202239_at	PARP4	1.197478705	855	14.02428668	0.021216374
225222_at	HIAT1	1.172022284	856	14.01875782	0.021285047
212146_at	PLEKHM2	1.115739322	857	13.99049941	0.021330222
1552701_a_at	COP1	1.584469622	858	13.98535948	0.021363636
205895_s_at	NOLC1	-1.257013375	859	13.96472368	0.02145518
1557985_s_at	CEP78	-1.114193651	860	13.96437822	0.021430233
209795_at	CD69	2.077718207	861	13.95071921	0.021521487
224043_s_at	UPB1	1.260503392	862	13.93519279	0.021670534

226978_at	PPARA	1.304954948	863	13.92671459	0.02170336
200957_s_at	SSRP1	-1.199139914	864	13.92580244	0.021678241
212379_at	GART	-1.21335356	865	13.92473961	0.021653179
228093_at	ZNF599	-1.246601194	866	13.91232141	0.02169746
203771_s_at	BLVRA	1.453972517	867	13.9014072	0.021787774
222347_at	LOC644450	2.140577397	868	13.89556321	0.021843318
226011_at	CCDC12	-1.118061851	869	13.89028086	0.021818182
228355_s_at	NDUFA12L	-1.158292806	870	13.88423109	0.021908046
205978_at	KL	-2.239225777	871	13.88004313	0.021882893
242260_at	MATR3	-1.902636553	872	13.87848258	0.021926606
219074_at	TMEM34	1.121943481	873	13.86941155	0.021981672
208595_s_at	MBD1	-1.22603486	874	13.86939399	0.021956522
215903_s_at	MAST2	-1.184271612	875	13.85737673	0.022045714
222534_s_at	C14orf173	-1.241427492	876	13.85731961	0.022020548
201327_s_at	CCT6A	-1.151089491	877	13.85542543	0.021995439
204113_at	CUGBP1	-1.304954948	878	13.85379222	0.021970387
226982_at	ELL2	1.434949535	879	13.84323065	0.022047782
230696_at	ATP2A1	-1.220946513	880	13.83021586	0.022113636
214426_x_at	CHAF1A	-1.167967395	881	13.82897591	0.022190692
203266_s_at	MAP2K4	1.126619228	882	13.82365574	0.022165533
210643_at	TNFSF11	-2.568631618	883	13.81883332	0.022242356
1552628_a_at	HERPUD2	1.267512522	884	13.81830529	0.022217195
221905_at	CYLD	1.278099363	885	13.8166838	0.02219209
225982_at	UBTF	-1.140763716	886	13.80428432	0.022268623
210293_s_at	SEC23B	1.154285418	887	13.80006134	0.022243517
212559_at	PRKAR1B	-1.33422317	888	13.79650713	0.02231982
218088_s_at	RRAGC	1.186736798	889	13.7905099	0.022294713
233021_at	AW292739	-1.119612889	890	13.78849459	0.022393258
205822_s_at	HMGCS1	-1.714752073	891	13.78614947	0.022368126
1567081_x_at	CLN6	-1.246601194	892	13.77637663	0.022387892
220370_s_at	USP36	-1.273677475	893	13.75614693	0.02256439
224336_s_at	DUSP16	1.294145654	894	13.7560318	0.02253915
217870_s_at	CMPK	1.121943481	895	13.73361553	0.022681564
202294_at	STAG1	1.158292806	896	13.7305995	0.02265625
223288_at	USP38	1.190031696	897	13.7284013	0.022697882
218921_at	SIGIRR	-1.23370717	898	13.7263169	0.022672606
221923_s_at	NPM1	-1.113421618	899	13.72432025	0.022647386
225364_at	STK4	1.108032348	900	13.71787258	0.022711111
217760_at	TRIM44	1.162314108	901	13.7152538	0.022685905
231647_s_at	FCRL5	2.718856484	902	13.70396081	0.022749446
215719_x_at	FAS	1.364147835	903	13.70291697	0.022724252
225144_at	BMPR2	1.236275261	904	13.69583698	0.022787611
37943_at	ZFYVE26	1.133669413	905	13.6925226	0.022762431
223226_x_at	SSBP4	-1.379360922	906	13.68838823	0.022825607
204374_s_at	GALK1	-1.261377409	907	13.68811798	0.022800441
203920_at	NR1H3	-1.123499903	908	13.67962317	0.02284141
230499_at	BIRC3	1.366987452	909	13.67120397	0.022816282
207395_at	BTN1A1	-1.494849249	910	13.66604711	0.022824176
219329_s_at	C2orf28	1.107264584	911	13.65941774	0.022854007
209501_at	CDR2	1.167967395	912	13.6575438	0.022828947
209578_s_at	POFUT2	1.2397077	913	13.65566699	0.022803943

211368_s_at	CASP1	2.425025638	914	13.65552978	0.022778993
1559444_at	AL833029	-1.101905116	915	13.64423393	0.022808743
212222_at	PSME4	1.237990291	916	13.64400128	0.022783843
211708_s_at	SCD	-1.607701981	917	13.64270577	0.022758997
243252_at	AA173465	1.384149716	918	13.6406311	0.022734205
203976_s_at	CHAF1A	-1.276328769	919	13.63944781	0.022752992
206059_at	ZNF91	1.226884977	920	13.63936613	0.022728261
222686_s_at	FLJ11151	-1.365093718	921	13.6362969	0.022703583
222019_at	VILL	-1.146312186	922	13.6357918	0.022678959
1552626_a_at	TMEM163	-1.72428709	923	13.62874541	0.022708559
228879_at	SNORA76	-1.304050735	924	13.62013764	0.022683983
217936_at	ARHGAP5	1.65175533	925	13.61864486	0.022756757
218905_at	INTS8	-1.096571589	926	13.61712462	0.022732181
218443_s_at	DAZAP1	-1.142346247	927	13.60586731	0.022815534
1554348_s_at	MGC13017	-1.139183377	928	13.60159597	0.022790948
225559_at	C3orf19	1.142346247	929	13.60134161	0.022766416
224162_s_at	FBXO31	1.163120042	930	13.59168327	0.022806452
223503_at	TMEM163	-3.050403307	931	13.58738004	0.022857143
224467_s_at	PDCD2L	-1.331451613	932	13.57260099	0.022972103
223336_s_at	RAB18	1.169587664	933	13.57177504	0.022947481
235577_at	AL036451	1.17609125	934	13.56906492	0.022997859
207113_s_at	TNF	-1.53261996	935	13.56603023	0.022973262
223449_at	SEMA6A	1.77399261	936	13.56426327	0.022948718
227164_at	SFRS1	-1.20664392	937	13.56324085	0.022924226
205992_s_at	IL15	1.496922987	938	13.56269112	0.022899787
223403_s_at	POLR1B	-1.155886707	939	13.55960797	0.022971246
224713_at	MKI67IP	-1.124278924	940	13.55932706	0.022946809
209080_x_at	TXNL2	-1.125838586	941	13.55859904	0.022922423
208709_s_at	NRD1	1.145517898	942	13.55698521	0.022898089
204837_at	MTMR9	1.257013375	943	13.54756166	0.022958643
232309_at	LOC202181	-1.216722359	944	13.54332832	0.022934322
203966_s_at	PPM1A	1.270150983	945	13.5432727	0.022910053
225622_at	PAG1	-1.529436278	946	13.53474175	0.02294926
218521_s_at	UBE2W	1.200803427	947	13.53428713	0.022925026
209732_at	CLEC2B	2.360348687	948	13.51597892	0.023175105
1558201_s_at	SLC4A1AP	1.096571589	949	13.51478589	0.023150685
221732_at	CANT1	1.108800644	950	13.51301311	0.023126316
212451_at	KIAA0256	1.426025717	951	13.50347701	0.023196635
214152_at	CCPG1	1.444930398	952	13.50189076	0.023172269
200096_s_at	ATP6V0E1	1.139973273	953	13.49929102	0.023273872
214564_s_at	PCDHGC3	-1.217566019	954	13.49728534	0.023249476
214028_x_at	TDRD3	1.21167266	955	13.492477	0.023225131
202377_at	AW026535	1.25962998	956	13.49219836	0.023200837
205607_s_at	SCYL3	1.232852325	957	13.49135213	0.023176594
202416_at	DNAJC7	-1.377450046	958	13.48967381	0.023204593
205194_at	PSPH	-1.158292806	959	13.47829224	0.023242961
205013_s_at	ADORA2A	1.281647924	960	13.46400655	0.023333333
227784_s_at	COG1	1.155886707	961	13.4623341	0.023309053
225429_at	BF437011	1.126619228	962	13.44608998	0.023492723
201500_s_at	PPP1R11	1.165541198	963	13.44603843	0.023468328
203232_s_at	ATXN1	1.447938172	964	13.44315211	0.023443983

219279_at	DOCK10	1.429984986	965	13.43440101	0.02346114
215346_at	CD40	1.426025717	966	13.42835469	0.023519669
57082_at	LDLRAP1	-1.143138335	967	13.42520779	0.023495346
213437_at	RUFY3	1.22603486	968	13.41946431	0.023533058
218947_s_at	PAPD1	-1.142346247	969	13.41084887	0.023508772
218409_s_at	DNAJC1	1.240567298	970	13.40859353	0.02357732
231973_s_at	ANAPC1	-1.155085785	971	13.40652619	0.023553038
227611_at	TARSL2	1.473247686	972	13.40483751	0.023528807
227517_s_at	CENPL	-1.224336392	973	13.40192	0.023504625
200714_x_at	OS9	1.219255094	974	13.40168384	0.023480493
74694_s_at	RABEP2	1.231998073	975	13.3961082	0.023507692
218481_at	EXOSC5	-1.244874235	976	13.3810813	0.02357582
205417_s_at	DAG1	1.172022284	977	13.37968122	0.023664278
204283_at	FARS2	-1.167158102	978	13.36288953	0.023711656
202194_at	TMED5	1.207480591	979	13.34772709	0.02381001
214151_s_at	CCPG1	1.373636233	980	13.34323969	0.023785714
211366_x_at	CASP1	2.370185542	981	13.33296572	0.023893986
225879_at	TSEN54	-1.173648178	982	13.31818774	0.024002037
204521_at	C12orf24	-1.25962998	983	13.30945632	0.024089522
222691_at	SLC35B3	1.150291893	984	13.30664955	0.024065041
209788_s_at	ARTS-1	1.644900137	985	13.30520053	0.024040609
214736_s_at	ADD1	1.227735684	986	13.30400955	0.024016227
224726_at	MIB1	1.313121125	987	13.29794492	0.024042553
208725_at	EIF2S2	-1.114193651	988	13.2972805	0.024018219
222528_s_at	SLC25A37	-1.393777239	989	13.29360027	0.023993933
204161_s_at	ENPP4	1.682958965	990	13.28285794	0.024060606
209970_x_at	CASP1	2.355445579	991	13.27716004	0.024106963
202978_s_at	CREBZF	-1.141554707	992	13.27095759	0.024082661
210024_s_at	UBE2E3	1.25962998	993	13.26830441	0.024159114
222105_s_at	NKIRAS2	1.110338834	994	13.26766333	0.024134809
206220_s_at	RASA3	-1.210833084	995	13.26028562	0.024110553
212911_at	DNAJC16	1.134455485	996	13.25746566	0.024136546
1555758_a_at	CDKN3	-1.187559666	997	13.25469017	0.024112337
222499_at	MRPS16	-1.155085785	998	13.24793171	0.024118236
208916_at	SLC1A5	-1.339783602	999	13.24273506	0.024094094
221796_at	NTRK2	2.37841423	1000	13.23793058	0.02415
205145_s_at	MYL5	1.171210181	1001	13.23598414	0.024125874
226024_at	COMMD1	1.212512819	1002	13.22952058	0.024211577
228469_at	PPID	1.282536603	1003	13.22579334	0.024187438
225397_at	CCDC32	1.286097483	1004	13.22354163	0.024163347
201494_at	PRCP	1.273677475	1005	13.22280308	0.024139303
202244_at	PSMB4	1.100378609	1006	13.22229346	0.024115308
214283_at	TMEM97	-1.232852325	1007	13.21904625	0.024210526
53912_at	SNX11	1.256142381	1008	13.21838329	0.024186508
215148_s_at	APBA3	1.214194884	1009	13.21675578	0.024162537
221502_at	KPNA3	-1.129747215	1010	13.21425468	0.024138614
201786_s_at	ADAR	1.104198847	1011	13.20987401	0.024223541
219328_at	DDX31	-1.113421618	1012	13.18960565	0.024407115
231811_at	FAM19A3	-1.180992661	1013	13.18436612	0.024383021
228980_at	RFFL	1.281647924	1014	13.18223117	0.024358974
204328_at	TMC6	1.192508872	1015	13.17967546	0.024413793

210457_x_at	HMGA1	-1.550786413	1016	13.17512045	0.024389764
212303_x_at	BG026366	-1.144724161	1017	13.17313559	0.024365782
224847_at	CDK6	1.383190629	1018	13.1704928	0.024341847
224851_at	CDK6	1.418140036	1019	13.15477332	0.024474975
229123_at	AI652703	1.281647924	1020	13.15340968	0.02445098
209606_at	PSCDBP	1.298638603	1021	13.15177432	0.024427032
226217_at	SLC30A7	1.198309021	1022	13.15109726	0.024403131
202905_x_at	NBN	1.227735684	1023	13.13742491	0.024643206
209142_s_at	UBE2G1	-1.174461971	1024	13.13356164	0.024619141
201755_at	MCM5	-1.255271991	1025	13.13279586	0.024595122
213509_x_at	CES2	1.125058485	1026	13.13102167	0.02457115
228749_at	KIAA1571	1.615521555	1027	13.12887158	0.024703019
221778_at	KIAA1718	-1.465100875	1028	13.12744196	0.024678988
1552360_a_at	TIRAP	1.250062303	1029	13.11282762	0.024771623
204357_s_at	LIMK1	1.229438867	1030	13.10848396	0.024893204
201970_s_at	NASP	-1.150291893	1031	13.10373961	0.024869059
218997_at	POLR1E	-1.159899655	1032	13.10129957	0.024844961
209464_at	AURKB	-1.215879283	1033	13.09877843	0.024898354
1560117_at	ABHD1	-1.144724161	1034	13.0910848	0.024874275
224185_at	TP53	-1.237132479	1035	13.09061959	0.024850242
220659_s_at	C7orf43	1.184271612	1036	13.08485472	0.024913127
204211_x_at	EIF2AK2	1.191682575	1037	13.07930567	0.024975892
218594_at	HEATR1	-1.105730653	1038	13.07758427	0.02495183
203050_at	TP53BP1	1.147107024	1039	13.074314	0.024927815
241682_at	KLHL23	-1.265756594	1040	13.07260746	0.024903846
41329_at	SCYL3	1.2397077	1041	13.07144414	0.024879923
200015_s_at	2-Sep	1.098854218	1042	13.06948598	0.025
221539_at	EIF4EBP1	-1.197478705	1043	13.06339474	0.024976031
224870_at	KIAA0114	-1.425037614	1044	13.06076359	0.024952107
227165_at	C13orf3	-1.29145735	1045	13.05754976	0.025014354
224871_at	FAM79A	1.23370717	1046	13.05615721	0.02499044
209939_x_at	CFLAR	1.426025717	1047	13.0526424	0.024966571
214177_s_at	PBXIP1	1.465100875	1048	13.05183213	0.024942748
216252_x_at	FAS	1.364147835	1049	13.04828024	0.024985701
204005_s_at	PAWR	-1.398616083	1050	13.04228881	0.024961905
210950_s_at	FDFT1	-1.17609125	1051	13.03883463	0.025052331
202735_at	EBP	-1.196648963	1052	13.03001176	0.025028517
223076_s_at	NSUN2	-1.109569472	1053	13.0235646	0.025071225
224606_at	KLF6	1.337000495	1054	13.02355433	0.025047438
210720_s_at	APBA2BP	-1.147107024	1055	13.02336674	0.025023697
213103_at	STARD13	1.537940831	1056	13.01746857	0.025066288
225337_at	ABHD2	1.152686347	1057	13.01226837	0.025042573
227141_at	TYW3	1.328685814	1058	13.00527742	0.025122873
227430_at	ZC3H10	1.090507733	1059	12.99423026	0.025184136
227728_at	AA886888	1.330529041	1060	12.9919624	0.025160377
236190_at	TLN1	1.502119927	1061	12.98670863	0.025202639
205554_s_at	DNASE1L3	2.089271526	1062	12.97853175	0.025291902
202065_s_at	PPFIA1	-1.350037985	1063	12.97658535	0.025268109
202646_s_at	CSDE1	1.131314463	1064	12.97518648	0.025244361
201132_at	HNRPH2	1.135242102	1065	12.97331798	0.025220657
210162_s_at	NFATC1	-1.453972517	1066	12.96781498	0.025318949

202809_s_at	INTS3	1.141554707	1067	12.96298747	0.02529522
225176_at	LNPEP	1.340712592	1068	12.95119466	0.025365169
212647_at	RRAS	1.282536603	1069	12.95045566	0.025341441
225672_at	GOLGA2	1.127400412	1070	12.94584839	0.025392523
224809_x_at	TINF2	1.111879158	1071	12.94279732	0.025368814
200911_s_at	TACC1	1.128964405	1072	12.93949752	0.025466418
230345_at	AI654547	1.271913007	1073	12.93836056	0.025442684
218001_at	MRPS2	-1.22603486	1074	12.92812877	0.025558659
202228_s_at	NPTN	1.154285418	1075	12.92329346	0.025534884
233655_s_at	FAM29A	-1.209155676	1076	12.92324222	0.025511152
227477_at	ZMYND19	-1.154285418	1077	12.91954687	0.025571031
233878_s_at	XRN2	-1.147902414	1078	12.91664572	0.02554731
218619_s_at	SUV39H1	-1.205807828	1079	12.91019325	0.025523633
1553436_at	LOC727928	1.957483301	1080	12.90848731	0.025666667
241483_at	AA156795	2.581124981	1081	12.90720887	0.025642923
211162_x_at	SCD	-1.631274987	1082	12.90489963	0.025619224
204096_s_at	ELL	1.155085785	1083	12.90136522	0.025595568
225157_at	MLXIP	-1.42800398	1084	12.8982251	0.025710332
1560228_at	SNAI3	-1.178539408	1085	12.89772559	0.025686636
224832_at	DUSP16	1.319507911	1086	12.89304209	0.025662983
AFFX-HUMISGI	STAT1	1.295940965	1087	12.89253387	0.025639374
200670_at	XBP1	1.446934886	1088	12.8828057	0.025735294
226104_at	RNF170	1.413233644	1089	12.87581126	0.025831038
1559867_at	LOC729013	1.194163187	1090	12.8737108	0.025807339
218974_at	FLJ10159	-1.462057448	1091	12.84950184	0.026076994
218302_at	PSENEN	1.156688184	1092	12.84764715	0.026053114
209944_at	ZNF410	1.093535457	1093	12.8466394	0.026029277
220161_s_at	EPB41L4B	-1.536875181	1094	12.83938066	0.026096892
202375_at	SEC24D	1.274560627	1095	12.83660192	0.026073059
227184_at	BF508702	-2.148008943	1096	12.83303106	0.02604927
201453_x_at	RHEB	1.092777739	1097	12.83153789	0.026025524
1552310_at	C15orf40	1.151089491	1098	12.82700801	0.026111111
226119_at	PCMTD1	1.382232207	1099	12.82661591	0.026087352
240106_at	GNPTAB	1.235418637	1100	12.82261061	0.026063636
205292_s_at	HNRPA2B1	-1.074004472	1101	12.8223333	0.026039964
227329_at	ZBTB46	1.183451022	1102	12.82161552	0.026016334
212642_s_at	HIVEP2	1.382232207	1103	12.81846915	0.026083409
213754_s_at	PAIP1	1.150291893	1104	12.81195881	0.026059783
218069_at	XTP3TPA	-1.170398641	1105	12.80979968	0.026117647
203414_at	MMD	1.379360922	1106	12.80109801	0.026094033
230656_s_at	CIRH1A	-1.127400412	1107	12.79708865	0.026205962
225621_at	ALG2	1.183451022	1108	12.79638222	0.02618231
221795_at	NTRK2	1.667862088	1109	12.78747903	0.026275924
212432_at	GRPEL1	-1.151089491	1110	12.77773194	0.026387387
220565_at	CCR10	-1.522033381	1111	12.77578103	0.026363636
1554014_at	CHD2	1.232852325	1112	12.75600468	0.026546763
236436_at	SLC25A45	1.183451022	1113	12.75559818	0.026522911
204759_at	RCBTB2	1.475291457	1114	12.75312867	0.026499102
212505_s_at	KIAA0892	1.098092814	1115	12.75286359	0.026475336
212186_at	ACACA	-1.167158102	1116	12.74998158	0.026550179
231124_x_at	LY9	1.502119927	1117	12.74424996	0.02652641

222988_s_at	TMEM9	1.236275261	1118	12.7335224	0.026654741
240681_at	AW118997	1.229438867	1119	12.73289334	0.02663092
226749_at	MRPS9	-1.127400412	1120	12.72868768	0.026678571
223886_s_at	RNF146	1.254402205	1121	12.72499788	0.026654773
201802_at	SLC29A1	-1.289668251	1122	12.72110141	0.026631016
225120_at	PURB	1.145517898	1123	12.72076333	0.026607302
205480_s_at	UGP2	1.078480432	1124	12.7164918	0.026717082
229269_x_at	SSBP4	-1.342572503	1125	12.71444231	0.026693333
218884_s_at	GUF1	-1.180174343	1126	12.71251949	0.026669627
215842_s_at	ATP11A	-1.243149669	1127	12.70939092	0.026796806
233825_s_at	CD99L2	1.262252032	1128	12.70936667	0.02677305
207176_s_at	CD80	1.371733289	1129	12.70820935	0.026749336
224965_at	GNG2	-1.318593614	1130	12.69818213	0.026831858
39582_at	CYLD	1.187559666	1131	12.69059846	0.026808134
239277_at	AI559696	1.209994089	1132	12.68882876	0.026943463
204147_s_at	TFDP1	-1.283425898	1133	12.68609158	0.026919682
229070_at	C6orf105	2.347296357	1134	12.68600036	0.026895944
212709_at	NUP160	-1.171210181	1135	12.68533786	0.026872247
46323_at	CANT1	1.122721422	1136	12.68293793	0.026848592
200086_s_at	COX4I1	1.048989328	1137	12.68080988	0.026824978
201891_s_at	B2M	1.057750964	1138	12.67820615	0.026880492
219120_at	C2orf44	-1.066585781	1139	12.67797867	0.026856892
214009_at	MSL3L1	1.424050196	1140	12.67418917	0.026833333
1554493_s_at	THADA	-1.082975046	1141	12.67191001	0.026809816
233936_s_at	ZNF403	1.240567298	1142	12.66566262	0.026908932
227094_at	DHTKD1	1.193335743	1143	12.66284267	0.026885389
202587_s_at	AK1	-1.257013375	1144	12.66024289	0.026861888
226221_at	KIAA1432	1.196648963	1145	12.65937535	0.026951965
236280_at	AI225238	1.258757174	1146	12.65279085	0.026928447
1555360_a_at	DNAJC11	-1.128964405	1147	12.64720068	0.027061901
200773_x_at	PTMA	-1.051172909	1148	12.64628478	0.027038328
213375_s_at	CG018	1.45296505	1149	12.64599531	0.027014795
203042_at	LAMP2	1.243149669	1150	12.64514911	0.026991304
206474_at	PCTK2	1.231144413	1151	12.63940563	0.027089487
218376_s_at	MICAL1	1.252664439	1152	12.63853171	0.027065972
216860_s_at	GDF11	-1.465100875	1153	12.63835758	0.027042498
242878_at	BF061275	1.215036792	1154	12.6383566	0.027019064
1564906_at	SNHG4	-1.237990291	1155	12.62724691	0.027090909
201832_s_at	PAK1	1.127400412	1156	12.61702138	0.027179931
217858_s_at	ARMCX3	1.240567298	1157	12.61639773	0.027156439
213278_at	MTMR9	1.165541198	1158	12.61571013	0.027132988
1053_at	RFC2	-1.232852325	1159	12.60957291	0.027195858
AFFX-HUMISGI	STAT1	1.247465572	1160	12.60588081	0.027172414
200620_at	TMEM59	1.193335743	1161	12.60350966	0.027149009
202067_s_at	LDLR	-1.32592576	1162	12.60263219	0.027125645
213210_at	TAF6L	-1.147107024	1163	12.59801915	0.0272227
226662_at	AW085575	1.181811547	1164	12.59084771	0.027199313
221864_at	TMEM142C	1.262252032	1165	12.5887843	0.027321888
216095_x_at	MTMR1	1.143138335	1166	12.58578287	0.027298456
215223_s_at	SOD2	-1.320422841	1167	12.58299059	0.027275064
209375_at	XPC	1.193335743	1168	12.56724804	0.027542808

209751_s_at	TRAPPC2	1.094293701	1169	12.56096919	0.027519247
1554415_at	TAF5L	-1.171210181	1170	12.55945771	0.027623932
237759_at	CD48	1.435944511	1171	12.55075025	0.027600342
209284_s_at	C3orf63	1.163120042	1172	12.54984511	0.027696246
235857_at	KCTD11	1.159095952	1173	12.54938779	0.027672634
1556183_at	FLJ40330	-2.148008943	1174	12.5481749	0.027649063
224753_at	CDCA5	-1.168777249	1175	12.54727591	0.027625532
243976_at	AW779916	1.162314108	1176	12.54528887	0.027602041
1568730_at	AW195885	-1.203303026	1177	12.54335305	0.02757859
239100_x_at	W03928	1.279872414	1178	12.53691576	0.027674024
217722_s_at	NGRN	1.190856849	1179	12.53330114	0.027650551
212413_at	6-Sep	-1.244011653	1180	12.53256199	0.027627119
219045_at	RHOF	1.231998073	1181	12.52603992	0.027730737
226478_at	TM7SF3	1.331451613	1182	12.52149998	0.027707276
202825_at	SLC25A4	-1.304050735	1183	12.51188254	0.027810651
218676_s_at	PCTP	1.394743666	1184	12.49422086	0.027989865
211458_s_at	GABARAPL1	2.435132037	1185	12.4886125	0.028059072
235451_at	SMAD5	1.260503392	1186	12.48813809	0.028035413
226128_at	C1orf58	1.190031696	1187	12.48747721	0.028011794
225785_at	REEP3	1.21335356	1188	12.48692852	0.027988215
220892_s_at	PSAT1	-1.462057448	1189	12.48469637	0.027964676
205644_s_at	SNRPG	-1.101905116	1190	12.48387325	0.027941176
215705_at	PPP5C	-1.086734863	1191	12.48075529	0.027917716
225826_at	MMAB	-1.197478705	1192	12.47689162	0.028028523
223776_x_at	TINF2	1.108800644	1193	12.47453918	0.028005029
202664_at	WIPF1	1.180992661	1194	12.4681123	0.028115578
227261_at	KLF12	1.69466487	1195	12.46185943	0.02809205
209777_s_at	SLC19A1	-1.269270886	1196	12.45929251	0.028152174
229109_s_at	BLVRA	1.284315809	1197	12.45444846	0.028128655
210407_at	PPM1A	1.278985581	1198	12.45358191	0.028105175
225485_at	TSGA14	-1.264003098	1199	12.44758691	0.028198499
210962_s_at	AKAP9	1.219255094	1200	12.44666293	0.028175
225901_at	PTPMT1	-1.144724161	1201	12.44322622	0.02815154
213196_at	ZNF629	1.268391399	1202	12.4418961	0.02812812
200959_at	FUS	-1.223488041	1203	12.43689451	0.028179551
206273_at	C18orf43	-1.333298677	1204	12.43023676	0.028156146
40225_at	GAK	1.193335743	1205	12.42987883	0.028240664
44040_at	FBXO41	-1.114193651	1206	12.42073897	0.028217247
225290_at	ETNK1	1.121166078	1207	12.4174767	0.028342999
227704_at	C19orf12	1.133669413	1208	12.41743524	0.028319536
202462_s_at	DDX46	-1.090507733	1209	12.41665919	0.028296112
209063_x_at	PAIP1	1.142346247	1210	12.41616758	0.028272727
201760_s_at	WSB2	1.237990291	1211	12.41612885	0.028249381
227395_at	BE672045	1.190856849	1212	12.41009997	0.028226073
223387_at	ZFYVE1	1.229438867	1213	12.40982975	0.028367683
218231_at	NAGK	1.341642225	1214	12.40978249	0.028344316
40446_at	PHF1	1.236275261	1215	12.39953543	0.028427984
212259_s_at	PBXIP1	1.38991822	1216	12.39902033	0.028404605
218770_s_at	TMEM39B	-1.106497353	1217	12.39608335	0.028381265
226468_at	AA778521	1.139973273	1218	12.39492542	0.028357964
213241_at	PLXNC1	1.32317144	1219	12.39138807	0.028334701

202733_at	P4HA2	1.894740141	1220	12.38092435	0.02845082
203282_at	GBE1	1.306765254	1221	12.3774218	0.028566749
212330_at	TFDP1	-1.143930973	1222	12.3695629	0.028674304
207132_x_at	PFDN5	1.146312186	1223	12.36226229	0.028650859
200742_s_at	TPP1	1.215879283	1224	12.35672263	0.02870915
201073_s_at	SMARCC1	-1.169587664	1225	12.35509776	0.028685714
238513_at	PRRG4	1.599920257	1226	12.35483626	0.028662316
221847_at	BF665706	1.140763716	1227	12.35206197	0.028638957
1554780_a_at	PHTF2	-1.094293701	1228	12.35062982	0.028615635
206518_s_at	RGS9	-1.242288282	1229	12.3454615	0.028706265
200807_s_at	HSPD1	-1.077733145	1230	12.34464385	0.028682927
208758_at	ATIC	-1.139183377	1231	12.34181581	0.028659626
205126_at	VRK2	1.252664439	1232	12.3366393	0.028766234
212345_s_at	CREB3L2	1.204972315	1233	12.33544923	0.028742903
226395_at	LOC286170	1.327765158	1234	12.33204918	0.028719611
215992_s_at	RAPGEF2	1.404444876	1235	12.32083713	0.028809717
1552703_s_at	CASP1	1.842928372	1236	12.30408067	0.028996764
242890_at	HELLS	-1.184271612	1237	12.30399834	0.028973323
214368_at	RASGRP2	-1.613283518	1238	12.30277723	0.028949919
202218_s_at	FADS2	-1.347233577	1239	12.30163679	0.028926554
231857_s_at	FLJ21839	-1.176906737	1240	12.29917953	0.029016129
200965_s_at	ABLIM1	-2.059080167	1241	12.29842486	0.028992748
229569_at	AW572379	1.33422317	1242	12.29720358	0.028969404
201458_s_at	BUB3	-1.17772279	1243	12.28856505	0.029026549
203059_s_at	PAPSS2	-1.236275261	1244	12.28824645	0.029003215
231873_at	BMPR2	1.148698355	1245	12.28420132	0.02897992
218670_at	PUS1	-1.152686347	1246	12.28310097	0.028956661
212961_x_at	CXorf40B	1.132098902	1247	12.2782377	0.029013633
231793_s_at	CAMK2D	1.615521555	1248	12.27549606	0.028990385
1556346_at	COTL1	-1.616641738	1249	12.27544878	0.028967174
226921_at	UBR1	1.143930973	1250	12.27479796	0.028944
212101_at	KPNA6	1.101141598	1251	12.27193514	0.028920863
205394_at	CHEK1	-1.199971382	1252	12.26681106	0.029017572
212520_s_at	SMARCA4	-1.173648178	1253	12.26167381	0.028994413
202644_s_at	TNFAIP3	1.318593614	1254	12.25606766	0.02907496
202240_at	PLK1	-1.274560627	1255	12.25160471	0.029051793
218562_s_at	ADD3	1.147902414	1256	12.25145486	0.029028662
202074_s_at	OPTN	1.281647924	1257	12.24805467	0.029101034
201115_at	POLD2	-1.151887642	1258	12.2468824	0.029077901
202107_s_at	MCM2	-1.181811547	1259	12.24294964	0.029054805
211949_s_at	NOLC1	-1.264879542	1260	12.23791146	0.029103175
202200_s_at	SRPK1	-1.099616149	1261	12.22920044	0.029167328
205660_at	OASL	1.469168633	1262	12.22824984	0.029144216
219687_at	HHAT	1.316766922	1263	12.22677152	0.02912114
239696_at	PRPSAP2	-1.180174343	1264	12.224171	0.029098101
208779_x_at	DDR1	1.270150983	1265	12.22126535	0.029075099
208732_at	RAB2	1.167967395	1266	12.22097144	0.029052133
202842_s_at	DNAJB9	1.481439798	1267	12.21539137	0.029194949
212043_at	TGOLN2	1.164733586	1268	12.20591979	0.029290221
216237_s_at	MCM5	-1.155886707	1269	12.20566856	0.029267139
202858_at	U2AF1	-1.098092814	1270	12.20233119	0.029244094

218518_at	C5orf5	1.229438867	1271	12.20213354	0.029221086
210205_at	B3GALT4	1.21335356	1272	12.20026815	0.029198113
211367_s_at	CASP1	2.572194967	1273	12.20021007	0.029175177
55692_at	ELMO2	1.169587664	1274	12.19812796	0.029309262
213378_s_at	DDX11	-1.231998073	1275	12.19649606	0.029286275
207375_s_at	IL15RA	1.308578071	1276	12.19163309	0.029263323
223441_at	SLC17A5	1.236275261	1277	12.18417274	0.029326547
226222_at	KIAA1432	1.21167266	1278	12.18225317	0.029303599
203035_s_at	PIAS3	1.133669413	1279	12.17917087	0.029366693
225978_at	FAM80B	-1.64832417	1280	12.17674102	0.02934375
201186_at	LRPAP1	1.268391399	1281	12.1705837	0.029320843
212185_x_at	MT2A	1.796264746	1282	12.16767189	0.029438378
202933_s_at	YES1	1.440929749	1283	12.16474136	0.029415433
227985_at	AI928513	1.673652485	1284	12.16286757	0.029392523
208982_at	PECAM1	1.626758396	1285	12.1571108	0.029509728
218272_at	FLJ20699	1.226884977	1286	12.15611752	0.029486781
209147_s_at	PPAP2A	1.36983298	1287	12.14626005	0.0295338
235196_at	AA447464	1.167967395	1288	12.1458577	0.02951087
208152_s_at	DDX21	-1.144724161	1289	12.13309689	0.029588829
217800_s_at	NDFIP1	1.516767545	1290	12.12910479	0.029728682
216515_x_at	PTMA	-1.074004472	1291	12.12297114	0.029705655
228954_at	LYSMD4	1.261377409	1292	12.11399373	0.029798762
219875_s_at	C1orf121	-1.175276328	1293	12.10979437	0.029876257
210786_s_at	FLI1	-1.333298677	1294	12.1083582	0.029853168
218065_s_at	TMEM9B	1.153485605	1295	12.10550715	0.029830116
209084_s_at	RAB28	1.157490217	1296	12.10377865	0.029807099
221566_s_at	NOL3	1.159899655	1297	12.0973905	0.029922899
1552676_at	UCN3	-1.130530567	1298	12.09646977	0.029899846
226603_at	SAMD9L	1.437936533	1299	12.09442294	0.029876828
200741_s_at	RPS27	1.028826708	1300	12.09383957	0.029853846
228941_at	AW295395	1.346300069	1301	12.08946063	0.029930822
213052_at	PRKAR2A	-1.151887642	1302	12.08782901	0.029907834
228671_at	KIAA0672	-1.136029265	1303	12.07788692	0.029953952
209980_s_at	SHMT1	-1.289668251	1304	12.07575221	0.029930982
205011_at	LOH11CR2A	1.172022284	1305	12.07211812	0.029908046
207996_s_at	C18orf1	1.654046737	1306	12.07129519	0.029885145
244103_at	C1orf55	1.159899655	1307	12.05800405	0.030091813
213346_at	C13orf27	-1.209155676	1308	12.05160538	0.030068807
203904_x_at	CD82	1.343503426	1309	12.05051254	0.030045837
223135_s_at	BBX	1.198309021	1310	12.0369001	0.030259542
210908_s_at	PFDN5	1.131314463	1311	12.0361846	0.030236461
232008_s_at	BBX	1.230291345	1312	12.02610777	0.030320122
203906_at	IQSEC1	1.328685814	1313	12.02424557	0.03029703
222553_x_at	OXR1	1.283425898	1314	12.02399035	0.030273973
211071_s_at	MLLT11	1.32317144	1315	12.01605194	0.030372624
205414_s_at	KIAA0672	-1.711190051	1316	12.01444323	0.030349544
227203_at	FBXL17	1.199971382	1317	12.01233827	0.0303265
218595_s_at	HEATR1	-1.087488391	1318	12.01104251	0.03030349
204093_at	CCNH	-1.091263877	1319	12.00706279	0.030394238
209994_s_at	ABCB1	1.549711862	1320	12.00604152	0.030371212
235399_at	KLC3	-1.117287138	1321	12.00400043	0.030348221

229934_at	N39230	-1.365093718	1322	12.00070114	0.030325265
210563_x_at	CFLAR	1.458009379	1323	11.99405619	0.030445956
218552_at	ECHDC2	1.470187336	1324	11.98814982	0.030536254
233656_s_at	VPS54	1.151887642	1325	11.98263025	0.030513208
1554102_a_at	TMTC4	-1.209994089	1326	11.98032025	0.030490196
224406_s_at	FCRL5	2.248557848	1327	11.97206627	0.03066315
63305_at	PKNOX2	-1.106497353	1328	11.97028516	0.03064006
225663_at	ACBD5	1.172834949	1329	11.97001142	0.030617005
222037_at	MCM4	-1.375541818	1330	11.96487101	0.030736842
212754_s_at	MON2	1.139183377	1331	11.96289231	0.030713749
201056_at	GOLGB1	1.226884977	1332	11.96090125	0.030690691
224669_at	SYS1	1.319507911	1333	11.9607369	0.030667667
219603_s_at	ZNF226	1.293248932	1334	11.96072298	0.030644678
201113_at	TUFM	-1.172834949	1335	11.95877194	0.030794007
235082_at	BG024649	1.220946513	1336	11.95833711	0.030770958
242873_at	KLRC4	2.103803558	1337	11.95250975	0.030747943
202486_at	AFG3L2	-1.126619228	1338	11.94842815	0.030859492
222883_at	C1orf163	-1.298638603	1339	11.94317113	0.030836445
1557192_at	C1orf136	-1.196648963	1340	11.9430771	0.030813433
211067_s_at	GAS7	1.491744027	1341	11.93196119	0.030969426
226601_at	SLC30A7	1.214194884	1342	11.92009275	0.031050671
225850_at	SFT2D1	1.160703914	1343	11.90789979	0.031221147
201929_s_at	PKP4	-1.352848231	1344	11.90164857	0.031197917
221811_at	PERLD1	1.105730653	1345	11.90149474	0.031174721
202510_s_at	TNFAIP2	1.604362333	1346	11.88632654	0.031426449
226331_at	BF508813	1.280759861	1347	11.88455883	0.031403118
223263_s_at	FGFR1OP2	1.171210181	1348	11.88125327	0.031379822
221613_s_at	ZFAND6	-1.258757174	1349	11.88083875	0.03135656
212985_at	BF115739	-2.431758566	1350	11.88045236	0.031333333
200927_s_at	RAB14	1.096571589	1351	11.87703219	0.031428571
214688_at	TLE4	1.394743666	1352	11.86933028	0.031531065
235152_at	FAM111B	1.248330549	1353	11.86807911	0.031507761
204127_at	RFC3	-1.163926534	1354	11.86415587	0.03148449
235241_at	FLJ90709	1.124278924	1355	11.86329358	0.031461255
222993_at	MRPL37	-1.142346247	1356	11.86234032	0.031438053
217766_s_at	TMEM50A	1.127400412	1357	11.85897953	0.031591746
202429_s_at	PPP3CA	1.611048582	1358	11.85279314	0.031568483
221664_s_at	F11R	1.330529041	1359	11.84393063	0.031707138
220495_s_at	C5orf14	1.235418637	1360	11.84260074	0.031683824
208447_s_at	PRPS1	-1.289668251	1361	11.83239737	0.031778104
219342_at	CASD1	1.127400412	1362	11.8313398	0.031754772
203146_s_at	GABBR1	1.165541198	1363	11.8261741	0.031870873
204905_s_at	EEF1E1	-1.20163605	1364	11.82538892	0.031847507
236831_at	CCDC50	1.349102534	1365	11.82301593	0.031824176
232589_at	AK021551	-1.225185332	1366	11.8226305	0.031800878
227586_at	TMEM170	1.159899655	1367	11.8197957	0.031880029
203405_at	DSCR2	-1.152686347	1368	11.80977297	0.031995614
240671_at	GYPC	-1.130530567	1369	11.80650488	0.031972243
231303_at	C21orf42	1.53261996	1370	11.80598143	0.031948905
222666_s_at	RCL1	-1.237990291	1371	11.80562409	0.031925602
207956_x_at	APRIN	-1.162314108	1372	11.80258623	0.031902332

202378_s_at	LEPROT	1.592176198	1373	11.79826219	0.032032047
202532_s_at	DHFR	-1.43097652	1374	11.78620051	0.032219796
218396_at	VPS13C	1.193335743	1375	11.77992172	0.032363636
214546_s_at	P2RY11	-1.210833084	1376	11.77970337	0.032340116
1555864_s_at	PDHA1	-1.102669163	1377	11.77744157	0.03231663
209969_s_at	STAT1	1.345367209	1378	11.77618129	0.032293179
218241_at	GOLGA5	1.139973273	1379	11.77455367	0.032269761
201209_at	HDAC1	-1.096571589	1380	11.76214248	0.03234058
214718_at	GATAD1	1.224336392	1381	11.7617703	0.032317161
212457_at	TFE3	1.199139914	1382	11.75877083	0.032416787
226166_x_at	STK36	1.129747215	1383	11.7582419	0.032393348
1558080_s_at	LOC144871	1.395710764	1384	11.75424036	0.032369942
227896_at	BCCIP	-1.170398641	1385	11.74946509	0.032534296
228765_at	GTF2IRD2	1.226884977	1386	11.73964268	0.032662338
212323_s_at	VPS13D	1.089752112	1387	11.73775188	0.032638789
226049_at	ERC1	1.212512819	1388	11.73747894	0.032615274
227066_at	MOBKL2C	1.151887642	1389	11.73103096	0.032591793
204216_s_at	ZC3H14	-1.095052471	1390	11.73072762	0.032568345
204300_at	PET112L	-1.134455485	1391	11.72432689	0.032717469
222701_s_at	CHCHD7	-1.164733586	1392	11.72197235	0.032693966
202046_s_at	GRLF1	-1.321338406	1393	11.71486747	0.032770998
238587_at	STS-1	-1.736280455	1394	11.70682414	0.032898135
218866_s_at	POLR3K	-1.224336392	1395	11.70675999	0.032874552
211519_s_at	KIF2C	-1.217566019	1396	11.70235205	0.032851003
220262_s_at	EGFL9	-1.084477409	1397	11.68965742	0.033092341
230672_at	AA521283	-1.21335356	1398	11.68539178	0.03306867
218984_at	PUS7	-1.279872414	1399	11.68315381	0.033045032
202230_s_at	CHERP	-1.105730653	1400	11.67523139	0.033164286
200832_s_at	SCD	-1.377450046	1401	11.67487695	0.033140614
225725_at	AL533234	1.391846392	1402	11.67115142	0.033116976
210564_x_at	CFLAR	1.483494934	1403	11.67037601	0.033093371
224797_at	ARRDC3	1.42899414	1404	11.66824879	0.033162393
236585_at	KIAA1147	1.162314108	1405	11.66326654	0.03313879
216520_s_at	TPT1	1.030968319	1406	11.65685172	0.033250356
208955_at	DUT	-1.289668251	1407	11.65306769	0.033226724
233960_s_at	LOC115110	1.107264584	1408	11.64785894	0.033352273
233849_s_at	ARHGAP5	1.56265576	1409	11.64489	0.033328602
201837_s_at	SUPT7L	1.098854218	1410	11.64328963	0.033304965
212948_at	CAMTA2	1.144724161	1411	11.64257336	0.033281361
200812_at	CCT7	-1.091263877	1412	11.6420988	0.03325779
223062_s_at	PSAT1	-1.403471726	1413	11.63601608	0.033368719
205153_s_at	CD40	1.390881972	1414	11.62393628	0.033451202
222426_at	MAPKAP1	1.212512819	1415	11.61893568	0.0335053
217781_s_at	ZFP106	-1.099616149	1416	11.61519146	0.033481638
226399_at	AL121021	1.203303026	1417	11.61099439	0.03345801
208828_at	POLE3	-1.242288282	1418	11.60897465	0.033568406
212207_at	THRAP2	-1.352848231	1419	11.60807984	0.03354475
228330_at	C6orf113	1.110338834	1420	11.60786028	0.033521127
224484_s_at	BRMS1L	1.316766922	1421	11.60328655	0.033497537
227682_at	BE645154	1.199971382	1422	11.60311641	0.03347398
224628_at	C2orf30	1.286097483	1423	11.60011411	0.033450457

226850_at	SUMF1	1.185092771	1424	11.59787685	0.033560393
1554672_at	TTC26	1.258757174	1425	11.59783282	0.033536842
223490_s_at	EXOSC3	-1.237132479	1426	11.5965146	0.033513324
204182_s_at	ZBTB43	1.183451022	1427	11.59494613	0.033489839
224703_at	WDR22	1.161508732	1428	11.59451444	0.033466387
222495_at	C1orf119	1.231144413	1429	11.59052242	0.033442967
209001_s_at	ANAPC13	1.159899655	1430	11.59019905	0.03341958
217371_s_at	IL15	1.351910833	1431	11.58480635	0.033529001
212870_at	RASSF3	1.227735684	1432	11.58381164	0.033505587
223846_at	AZI2	1.247465572	1433	11.57150018	0.033677599
218423_x_at	VPS54	1.133669413	1434	11.56546199	0.033877266
1553709_a_at	PRPF38A	-1.104964485	1435	11.56347028	0.033853659
214735_at	PIP3-E	2.540301965	1436	11.54231535	0.034129526
202086_at	MX1	1.185914499	1437	11.54149144	0.034105776
212957_s_at	LOC92249	1.139973273	1438	11.51896185	0.034499305
202761_s_at	SYNE2	1.643760375	1439	11.51859764	0.03447533
1554101_a_at	TMTC4	-1.319507911	1440	11.51561798	0.034451389
218605_at	TFB2M	-1.119612889	1441	11.51540801	0.034427481
220603_s_at	MCTP2	2.042024251	1442	11.5022761	0.034535368
210538_s_at	BIRC3	1.355664327	1443	11.48926647	0.034934165
202630_at	APPBP2	1.250062303	1444	11.48699905	0.034909972
201778_s_at	KIAA0494	1.136029265	1445	11.48536195	0.034885813
202183_s_at	KIF22	-1.17772279	1446	11.48053111	0.034861687
200633_at	UBB	1.054091423	1447	11.47462522	0.035017277
221940_at	RPUSD2	-1.101905116	1448	11.47431921	0.034993094
217918_at	DYNLRB1	1.132883885	1449	11.46414618	0.035162181
217640_x_at	C18orf24	-1.215879283	1450	11.46208827	0.035137931
204282_s_at	FARS2	-1.109569472	1451	11.46040831	0.035113715
215833_s_at	SPPL2B	-1.151887642	1452	11.45516472	0.035261708
202969_at	AI216690	-1.340712592	1453	11.45114576	0.03523744
202308_at	SREBF1	-1.449946833	1454	11.44561409	0.035385144
220465_at	FLJ12355	-1.246601194	1455	11.44223374	0.035360825
212845_at	SAMD4A	-1.483494934	1456	11.44124646	0.035336538
234306_s_at	SLAMF7	1.393777239	1457	11.44015315	0.035312286
224060_s_at	DPH5	-1.136029265	1458	11.43243501	0.035466392
230223_at	C14orf131	1.139183377	1459	11.43148933	0.035442084
203569_s_at	OFD1	1.311302014	1460	11.43117167	0.035417808
212469_at	NIPBL	-1.219255094	1461	11.42827424	0.035585216
223273_at	C14orf142	-1.210833084	1462	11.42756398	0.035560876
229485_x_at	LOC152573	-1.489677463	1463	11.42447266	0.035536569
222423_at	NDFIP1	1.219255094	1464	11.42385622	0.035512295
224182_x_at	SEMA6B	-1.114193651	1465	11.42367503	0.035488055
208809_s_at	C6orf62	1.139183377	1466	11.41983856	0.035620737
210884_s_at	SPAG11	-1.168777249	1467	11.41621445	0.035596455
238516_at	BMPR2	1.215879283	1468	11.41313644	0.035572207
222138_s_at	WDR13	1.168777249	1469	11.40477349	0.035724983
217504_at	ABCA6	1.495885758	1470	11.40455573	0.03570068
227729_at	ZNF211	1.095052471	1471	11.39706617	0.035805574
210592_s_at	SAT1	1.526259209	1472	11.39692754	0.03578125
232115_at	SLC39A3	-1.139973273	1473	11.39516783	0.035756959
226099_at	ELL2	1.372684431	1474	11.38983169	0.035820896

224405_at	FCRL5	2.457173961	1475	11.38980573	0.03579661
225687_at	FAM83D	-1.153485605	1476	11.38582363	0.035772358
217837_s_at	VPS24	1.138394029	1477	11.38309566	0.035748138
228356_at	ANKRD11	1.162314108	1478	11.38273659	0.035723951
203058_s_at	PAPSS2	-1.25962998	1479	11.38176407	0.035699797
204327_s_at	ZNF202	-1.128964405	1480	11.38001723	0.035675676
1563104_at	RAB11FIP3	-1.127400412	1481	11.37016288	0.035779878
222657_s_at	UBE2W	1.164733586	1482	11.36858797	0.035931174
212188_at	KCTD12	-1.918528239	1483	11.35984491	0.036035064
225289_at	STAT3	1.245737416	1484	11.35899343	0.036010782
212069_s_at	KIAA0515	-1.194163187	1485	11.35680052	0.035986532
213310_at	EIF2C2	-1.271031689	1486	11.35379798	0.035962315
211825_s_at	FLI1	-1.276328769	1487	11.35374443	0.03593813
211260_at	BMP7	-1.116512962	1488	11.35069721	0.035913978
203171_s_at	KIAA0409	-1.121943481	1489	11.3480701	0.036030893
226848_at	NR2C2	-1.113421618	1490	11.34559703	0.036006711
226476_s_at	VPRBP	-1.123499903	1491	11.34532441	0.035982562
240058_at	AI949799	1.747145792	1492	11.34239521	0.035958445
206653_at	POLR3G	-1.360370852	1493	11.34037252	0.03593436
216397_s_at	BOP1	-1.209994089	1494	11.33566703	0.036044177
1564911_at	SNHG4	-1.260503392	1495	11.32165636	0.036173913
209166_s_at	MAN2B1	1.17609125	1496	11.31223796	0.036296791
208890_s_at	PLXNB2	1.300440147	1497	11.30800678	0.036432866
222875_at	DHX33	-1.17609125	1498	11.30694323	0.036408545
225583_at	UXS1	1.460032011	1499	11.30300409	0.036384256
217993_s_at	MAT2B	1.151089491	1500	11.30007662	0.03636
221510_s_at	GLS	1.144724161	1501	11.29946569	0.03649567
224943_at	BTBD7	1.220946513	1502	11.28168395	0.036611185
215264_at	EMX1	1.240567298	1503	11.27970305	0.036739854
219257_s_at	SPHK1	-1.088242442	1504	11.27778079	0.036715426
226058_at	MGC4655	1.237132479	1505	11.27649058	0.03669103
207791_s_at	RAB1A	1.151089491	1506	11.27504492	0.036666667
238022_at	LOC643911	-2.987626914	1507	11.27198601	0.036642336
223411_at	MIF4GD	1.121166078	1508	11.27095456	0.036618037
204249_s_at	LMO2	-2.177994031	1509	11.26742325	0.036713055
228019_s_at	MRPS18C	-1.083725967	1510	11.26252821	0.036688742
202742_s_at	PRKACB	1.496922987	1511	11.26167872	0.036664461
229631_at	DNHD1	-1.219255094	1512	11.25001373	0.036772487
229881_at	KLF12	1.409320755	1513	11.24991416	0.036873761
205698_s_at	MAP2K6	1.420107359	1514	11.24187134	0.036849406
227627_at	SGK3	1.395710764	1515	11.24119131	0.036825083
1553148_a_at	SNX13	1.199139914	1516	11.23957099	0.036992084
1555039_a_at	ABCC4	-1.446934886	1517	11.2336987	0.036967699
50314_i_at	C20orf27	-1.311302014	1518	11.23082976	0.036943347
235146_at	N51717	1.234562607	1519	11.22545741	0.037030941
214994_at	APOBEC3F	1.118837101	1520	11.21900612	0.037217105
226727_at	LOC284106	-1.170398641	1521	11.21492209	0.037192636
52940_at	SIGIRR	-1.316766922	1522	11.21404656	0.0371682
225943_at	NLN	-1.205807828	1523	11.21390561	0.037143795
226109_at	C21orf91	1.342572503	1524	11.20780896	0.037322835
1554915_a_at	2'-PDE	-1.133669413	1525	11.20264902	0.037298361

219211_at	USP18	1.453972517	1526	11.19934304	0.037411533
218627_at	DRAM	1.408344227	1527	11.19825081	0.037387033
214999_s_at	RAB11FIP3	-1.150291893	1528	11.19296589	0.037362565
201097_s_at	ARF4	1.112650121	1529	11.19272476	0.037338129
223215_s_at	C14orf100	1.118837101	1530	11.19253636	0.037313725
244026_at	ELL2	1.721898377	1531	11.19073985	0.037289353
210819_x_at	DIO2	-1.116512962	1532	11.18635613	0.037382507
235081_x_at	TRIM65	-1.171210181	1533	11.18578723	0.037358121
203096_s_at	RAPGEF2	1.614402149	1534	11.18308642	0.037333768
200696_s_at	GSN	1.270150983	1535	11.17578567	0.037459283
236416_at	AI681617	1.147107024	1536	11.17300998	0.037434896
214431_at	GMPS	-1.115739322	1537	11.1667728	0.03754717
227022_at	GNPDA2	1.148698355	1538	11.15511305	0.037691808
224985_at	BE964484	1.092020546	1539	11.15162844	0.037667316
219473_at	GDAP2	1.088242442	1540	11.14942247	0.037792208
201927_s_at	PKP4	-1.236275261	1541	11.14419637	0.037767683
203132_at	RB1	1.56265576	1542	11.13839983	0.037944228
229304_s_at	MLF1IP	-1.243149669	1543	11.13171267	0.037919637
219131_at	UBIAD1	-1.132883885	1544	11.13058682	0.037895078
219522_at	FJX1	-1.275444392	1545	11.12546782	0.03797411
225657_at	LOC152217	-1.145517898	1546	11.12426411	0.037949547
218281_at	MRPL48	-1.151089491	1547	11.12250453	0.037925016
201758_at	TSG101	1.151887642	1548	11.1220485	0.037900517
219463_at	C20orf103	3.540615058	1549	11.11607303	0.038095546
203460_s_at	PSEN1	1.180992661	1550	11.11281331	0.038070968
218558_s_at	MRPL39	-1.139973273	1551	11.11280481	0.038046422
201924_at	AFF1	1.2397077	1552	11.11251663	0.038021907
202956_at	ARFGEF1	1.163926534	1553	11.10838024	0.03818416
202160_at	CREBBP	1.187559666	1554	11.10722005	0.038159588
201352_at	YME1L1	1.10343374	1555	11.10562993	0.038135048
214446_at	ELL2	1.439931319	1556	11.10180709	0.03811054
210385_s_at	ARTS-1	1.278985581	1557	11.09885493	0.038233783
220685_at	FAM120C	-1.109569472	1558	11.09838632	0.038209243
223342_at	RRM2B	1.266634254	1559	11.09707197	0.038184734
213261_at	LBA1	1.635804117	1560	11.09172252	0.038160256
218715_at	UTP6	-1.105730653	1561	11.08636932	0.038321589
202906_s_at	NBN	1.21167266	1562	11.08050846	0.038297055
214213_x_at	LMNA	1.215879283	1563	11.07258697	0.038394114
232617_at	CTSS	1.346300069	1564	11.07097813	0.038369565
203450_at	PGEA1	1.185914499	1565	11.06843232	0.038485623
226628_at	THOC2	-1.200803427	1566	11.05944393	0.038627075
205270_s_at	LCP2	-1.783857039	1567	11.05295267	0.038602425
201221_s_at	SNRP70	-1.076986376	1568	11.04777115	0.038743622
204598_at	UBOX5	-1.124278924	1569	11.04166069	0.038718929
202734_at	TRIP10	1.22858698	1570	11.0358718	0.038796178
213940_s_at	FNBP1	1.147107024	1571	11.03380027	0.038771483
236384_at	ITGAE	-1.161508732	1572	11.03364041	0.038746819
229652_s_at	LOC400236	-1.199139914	1573	11.02638848	0.038868404
239917_at	VPS8	1.088997015	1574	11.02542432	0.03884371
226373_at	SFXN5	1.150291893	1575	11.02443761	0.038819048
203984_s_at	CASP9	1.136029265	1576	11.02435903	0.038794416

214615_at	P2RY10	1.220946513	1577	11.02387282	0.038769816
203459_s_at	VPS16	1.110338834	1578	11.02371816	0.038745247
235580_at	ZNF141	-1.224336392	1579	11.01460198	0.038860038
218384_at	CARHSP1	-1.276328769	1580	11.01263535	0.038835443
215973_at	HCG4P6	-1.215879283	1581	11.01031139	0.038810879
221449_s_at	ITFG1	1.151887642	1582	11.00982222	0.038931732
209017_s_at	LONP1	-1.142346247	1583	11.00953098	0.038907138
221702_s_at	TM2D3	1.218410264	1584	11.00818277	0.038882576
209832_s_at	CDT1	-1.360370852	1585	11.00742053	0.038858044
217917_s_at	DYNLRB1	1.129747215	1586	11.00533197	0.038833544
206337_at	CCR7	1.608816742	1587	11.00493687	0.038809074
204415_at	IFI6	1.497960934	1588	11.00129163	0.038784635
203484_at	SEC61G	1.082224645	1589	10.99131588	0.038930145
1569448_at	PGM2L1	-1.126619228	1590	10.98502911	0.039075472
209825_s_at	UCK2	-1.178539408	1591	10.97895991	0.039170333
209522_s_at	CRAT	1.335148303	1592	10.97809519	0.039145729
218924_s_at	CTBS	1.204137381	1593	10.97493436	0.039121155
213331_s_at	NEK1	1.16634937	1594	10.96988824	0.039228356
207348_s_at	LIG3	-1.129747215	1595	10.96817539	0.039203762
210648_x_at	SNX3	1.138394029	1596	10.9677018	0.039179198
218076_s_at	ARHGAP17	1.282536603	1597	10.96752518	0.039154665
209134_s_at	RPS6	-1.025267238	1598	10.96304379	0.039130163
204023_at	RFC4	-1.147902414	1599	10.95311877	0.039274547
212880_at	WDR7	1.119612889	1600	10.95018034	0.03925
226223_at	PAWR	-1.365093718	1601	10.947092	0.039331668
228113_at	RAB37	-1.400556321	1602	10.94675813	0.039307116
212092_at	PEG10	-1.657489809	1603	10.94217646	0.039282595
219910_at	HYPE	1.336074078	1604	10.92419897	0.039600998
201139_s_at	SSB	-1.176906737	1605	10.92195496	0.039576324
1554151_at	OGDH	-1.091263877	1606	10.90798485	0.039937733
202010_s_at	ZNF410	1.068065408	1607	10.9043499	0.039912881
220742_s_at	NGLY1	1.199971382	1608	10.89349651	0.04011194
219731_at	FLJ34077	-1.101141598	1609	10.88601144	0.040261032
225956_at	LOC153222	1.331451613	1610	10.88063304	0.040236025
205569_at	LAMP3	1.446934886	1611	10.87863729	0.040415891
231041_at	POLR1E	-1.138394029	1612	10.8764537	0.040390819
202643_s_at	TNFAIP3	1.33422317	1613	10.87346632	0.040365778
202097_at	NUP153	-1.070288698	1614	10.87130771	0.040340768
229863_s_at	TMEM103	-1.123499903	1615	10.87016045	0.040315789
212949_at	NCAPH	-1.181811547	1616	10.86132875	0.04042698
208691_at	TFRC	-1.106497353	1617	10.86112343	0.040401979
208438_s_at	FGR	1.378405153	1618	10.86035552	0.040377009
224523_s_at	C3orf26	-1.163926534	1619	10.8597323	0.040525015
217853_at	TNS3	1.72428709	1620	10.85680084	0.0405
217927_at	SPCS1	1.112650121	1621	10.84661697	0.040623072
1554519_at	CD80	1.350037985	1622	10.843499	0.040598027
241429_at	AW263035	1.190856849	1623	10.84185617	0.040573013
225562_at	RASA3	-1.55293775	1624	10.83758947	0.040720443
213478_at	KIAA1026	1.513616793	1625	10.83707891	0.040695385
213956_at	CEP350	1.278985581	1626	10.83655905	0.040670357
201432_at	CAT	1.172022284	1627	10.83578683	0.04064536

213434_at	STX2	1.203303026	1628	10.83016248	0.040620393
219322_s_at	WDR8	1.224336392	1629	10.82588946	0.040742787
225660_at	SEMA6A	1.330529041	1630	10.82579273	0.040717791
227865_at	C9orf103	1.135242102	1631	10.8200274	0.040692826
205277_at	PRDM2	1.280759861	1632	10.81711963	0.040882353
213320_at	PRMT3	-1.237132479	1633	10.80675403	0.041034905
229227_at	FLJ45244	-1.07997656	1634	10.80673589	0.041009792
239186_at	MGC39372	1.420107359	1635	10.80519388	0.040984709
33304_at	ISG20	1.609932275	1636	10.80393926	0.040959658
1559052_s_at	PAK2	-1.22010051	1637	10.80272009	0.040934637
222280_at	GAPDHS	-1.197478705	1638	10.80174454	0.040909646
222896_at	TMEM38A	-1.352848231	1639	10.80153266	0.040884686
204690_at	STX8	1.159095952	1640	10.79542409	0.041079268
213473_at	BRAP	1.121943481	1641	10.79451276	0.041054235
225683_x_at	PHPT1	1.196648963	1642	10.7911066	0.041029233
212563_at	BOP1	-1.356604327	1643	10.78782585	0.041235545
202143_s_at	COPS8	-1.099616149	1644	10.78257605	0.041210462
202343_x_at	COX5B	1.080725402	1645	10.78195803	0.04118541
213220_at	LOC92482	1.169587664	1646	10.77456502	0.041324423
218622_at	NUP37	-1.155085785	1647	10.76122035	0.04143898
203595_s_at	IFIT5	1.338855257	1648	10.76051886	0.041413835
219491_at	LRFN4	-1.335148303	1649	10.7571823	0.041534263
232253_at	RAD50	1.403471726	1650	10.75509404	0.041509091
235134_at	BF969544	-1.250062303	1651	10.75431121	0.041483949
218652_s_at	PIGG	1.114966219	1652	10.75287774	0.041458838
209628_at	NXT2	1.171210181	1653	10.7524213	0.041433757
214268_s_at	MTMR4	1.132098902	1654	10.74900617	0.041571947
205780_at	BIK	-1.863480859	1655	10.7461051	0.041546828
225036_at	SHB	-1.097331938	1656	10.74059898	0.041521739
225866_at	BXDC1	-1.155085785	1657	10.73303286	0.041707906
201960_s_at	MYCBP2	1.182631	1658	10.73164649	0.04168275
229587_at	SAE2	-1.135242102	1659	10.7297875	0.041862568
201339_s_at	SCP2	1.07549439	1660	10.72747666	0.041837349
203116_s_at	FECH	1.190031696	1661	10.72722102	0.041812161
227245_at	C12orf30	-1.180992661	1662	10.7091753	0.042154031
243932_at	AI286254	1.617762697	1663	10.70491544	0.042128683
209594_x_at	PSG9	-1.10343374	1664	10.6980525	0.042247596
221691_x_at	NPM1	-1.062895674	1665	10.69206683	0.042222222
202988_s_at	RGS1	1.71832151	1666	10.68864437	0.042352941
224619_at	CASC4	1.118837101	1667	10.68387493	0.042327534
209383_at	DDIT3	1.168777249	1668	10.68223033	0.042302158
229466_at	LOC256273	-1.199971382	1669	10.68206945	0.042276812
202672_s_at	ATF3	1.531557997	1670	10.67534738	0.042413174
229253_at	THEM4	-1.293248932	1671	10.66924514	0.042555356
219147_s_at	C9orf95	1.45296505	1672	10.66602114	0.042529904
202236_s_at	SLC16A1	-1.21335356	1673	10.66432742	0.042504483
238600_at	JAKMIP1	-2.294214048	1674	10.66089988	0.042479092
206335_at	GALNS	1.223488041	1675	10.65911188	0.042579104
208692_at	RPS3	-1.052631155	1676	10.65825139	0.042553699
217197_x_at	CG018	1.28788163	1677	10.65634532	0.042528324
213133_s_at	GCSH	-1.164733586	1678	10.65589989	0.04250298

211113_s_at	ABCG1	1.4063932	1679	10.65565115	0.042477665
213450_s_at	ICOSLG	-1.234562607	1680	10.65387179	0.042452381
208679_s_at	ARPC2	1.060687741	1681	10.6526775	0.042427127
201383_s_at	NBR1	1.145517898	1682	10.65246421	0.042401902
204698_at	ISG20	1.652900636	1683	10.64046973	0.042525253
202718_at	IGFBP2	-2.522754818	1684	10.63999311	0.042713777
221460_at	OR2C1	-1.118061851	1685	10.63117651	0.042688427
209089_at	RAB5A	1.10343374	1686	10.6290471	0.042793594
242870_at	FAM80B	-1.503161478	1687	10.62751882	0.042768228
202420_s_at	DHX9	-1.094293701	1688	10.62128601	0.042742891
207821_s_at	PTK2	-1.659789171	1689	10.61960623	0.042895204
218205_s_at	MKNK2	1.154285418	1690	10.61895578	0.042869822
232034_at	LOC203274	-1.230291345	1691	10.61878588	0.042844471
223262_s_at	FGFR1OP2	1.136816973	1692	10.61545862	0.042819149
208467_at	KLF12	1.182631	1693	10.60900952	0.043024217
215091_s_at	GTF3A	-1.106497353	1694	10.60791765	0.042998819
223100_s_at	NUDT5	-1.109569472	1695	10.60516277	0.042973451
219200_at	FASTKD3	-1.093535457	1696	10.59955691	0.043142689
203615_x_at	SULT1A1	-1.182631	1697	10.59694343	0.043117266
1554462_a_at	DNAJB9	1.448942155	1698	10.59149448	0.043091873
224630_at	C2orf30	1.248330549	1699	10.58401967	0.043243084
212292_at	SLC7A1	-1.151887642	1700	10.58375856	0.043217647
224700_at	STT3B	1.20664392	1701	10.58254094	0.04319224
214112_s_at	CXorf40A	1.127400412	1702	10.58239751	0.043166863
225143_at	SFXN4	-1.17772279	1703	10.58086358	0.043141515
218055_s_at	WDR41	1.315854525	1704	10.57968676	0.043339202
219675_s_at	UXS1	1.400556321	1705	10.57968193	0.043313783
212538_at	DOCK9	2.039195366	1706	10.57859696	0.043288394
223002_s_at	XRN2	-1.082975046	1707	10.57654451	0.043263035
201059_at	CTTN	1.437936533	1708	10.57322887	0.043237705
202179_at	BLMH	-1.130530567	1709	10.56900071	0.043358689
200753_x_at	SFRS2	-1.116512962	1710	10.56254685	0.043333333
201387_s_at	UCHL1	-2.820595921	1711	10.55817301	0.043448276
226005_at	UBE2G1	-1.157490217	1712	10.5575513	0.043422897
224893_at	DKFZP564J086	1.140763716	1713	10.55358239	0.043397548
239856_at	AI701798	-1.188383105	1714	10.54965485	0.043523921
201649_at	UBE2L6	1.28877463	1715	10.5482844	0.043498542
227042_at	LOC150223	-1.116512962	1716	10.54348843	0.043473193
228738_at	D2HGDH	-1.247465572	1717	10.54038258	0.043447874
213763_at	HIPK2	-1.327765158	1718	10.53803127	0.04355064
218871_x_at	GALNACT-2	1.263127262	1719	10.53751986	0.043525305
206033_s_at	DSC3	2.236123702	1720	10.53589613	0.0435
223245_at	STRBP	-1.130530567	1721	10.53344286	0.043474724
237215_s_at	TFRC	-1.395710764	1722	10.53280023	0.043449477
207315_at	CD226	1.635804117	1723	10.53167808	0.04342426
220985_s_at	RNF170	1.337927555	1724	10.52951684	0.043578886
201318_s_at	MRCL3	1.060687741	1725	10.52410339	0.043553623
202241_at	TRIB1	1.598811661	1726	10.52372631	0.043528389
202384_s_at	TCOF1	-1.191682575	1727	10.51824454	0.0437348
202009_at	TWF2	-1.125058485	1728	10.51497567	0.043709491
208821_at	SNRPB	-1.115739322	1729	10.5100272	0.043684211

203907_s_at	IQSEC1	1.254402205	1730	10.50456578	0.043895954
205053_at	PRIM1	-1.194163187	1731	10.50084667	0.043870595
208991_at	STAT3	1.271031689	1732	10.49523789	0.04404157
209714_s_at	CDKN3	-1.151887642	1733	10.49418257	0.044016157
224865_at	MLSTD2	-1.128964405	1734	10.49087886	0.043990773
229711_s_at	MGC5370	1.223488041	1735	10.48639095	0.044115274
214755_at	UAP1L1	-1.435944511	1736	10.48439462	0.044089862
218174_s_at	C10orf57	1.141554707	1737	10.48430599	0.044064479
204092_s_at	AURKA	-1.151887642	1738	10.48260427	0.044039125
242807_at	FSD1L	1.378405153	1739	10.47900515	0.044209316
212592_at	IGJ	1.640345822	1740	10.477215	0.044183908
202638_s_at	ICAM1	1.548638056	1741	10.47154959	0.04415853
203347_s_at	MTF2	-1.162314108	1742	10.46841109	0.04435132
208149_x_at	DDX11	-1.164733586	1743	10.4613845	0.044325875
200759_x_at	NFE2L1	1.171210181	1744	10.46122958	0.044300459
202902_s_at	CTSS	1.286097483	1745	10.46057834	0.044275072
202522_at	PITPNB	1.066585781	1746	10.46012604	0.044249714
204567_s_at	ABCG1	2.114036081	1747	10.45800178	0.044396108
224636_at	ZFP91	1.088997015	1748	10.45505799	0.044370709
204512_at	HIVEP1	1.227735684	1749	10.45070499	0.04434534
240318_at	AFMID	-1.114966219	1750	10.44848784	0.044508571
201801_s_at	SLC29A1	-1.455989549	1751	10.44721518	0.044483152
218242_s_at	SUV420H1	1.180992661	1752	10.44385899	0.044457763
201027_s_at	EIF5B	-1.118061851	1753	10.44352816	0.044432402
217538_at	RUTBC1	1.141554707	1754	10.43938594	0.044640821
219460_s_at	TMEM127	1.124278924	1755	10.43444338	0.044615385
218767_at	REXO4	-1.085229372	1756	10.43429472	0.044589977
223064_at	LOC51255	1.102669163	1757	10.43264891	0.044564599
217777_s_at	PTPLAD1	-1.252664439	1758	10.43177583	0.044539249
239790_s_at	EXOSC2	-1.164733586	1759	10.42983888	0.044701535
229973_at	C1orf173	1.328685814	1760	10.42952945	0.044676136
202847_at	PCK2	-1.139973273	1761	10.42858927	0.044650767
1553132_a_at	MTAC2D1	1.707635429	1762	10.42855614	0.044625426
35150_at	CD40	1.319507911	1763	10.42288396	0.044600113
213988_s_at	SAT1	1.488645255	1764	10.41525561	0.044761905
222239_s_at	INTS6	1.108800644	1765	10.41432711	0.044736544
207618_s_at	BCS1L	-1.115739322	1766	10.41418105	0.044711212
200937_s_at	RPL5	-1.041021598	1767	10.41390849	0.044685908
203826_s_at	PITPNM1	-1.293248932	1768	10.41147201	0.044660633
1553940_a_at	LRRC28	1.219255094	1769	10.40975928	0.044850198
209045_at	XPNPEP1	1.194163187	1770	10.40841748	0.044824859
228003_at	RAB30	1.565908593	1771	10.40611159	0.044799548
212635_at	AW161626	1.092777739	1772	10.40609518	0.044774266
209667_at	CES2	1.110338834	1773	10.40532823	0.044749013
1558290_a_at	PVT1	1.188383105	1774	10.40471928	0.044723788
224924_at	TTC7A	-1.224336392	1775	10.4031682	0.044698592
222545_s_at	C10orf57	1.077733145	1776	10.40298536	0.044673423
204372_s_at	KHSRP	-1.111108729	1777	10.39719697	0.0448565
228531_at	SAMD9	1.376495602	1778	10.39228163	0.044831271
207980_s_at	CITED2	1.387992719	1779	10.39168388	0.044806071
219083_at	SHQ1	-1.251796459	1780	10.38662311	0.044955056

215711_s_at	WEE1	-1.163926534	1781	10.3845891	0.044929815
204717_s_at	SLC29A2	-1.295042999	1782	10.38306261	0.044904602
225099_at	FBXO45	-1.131314463	1783	10.38293062	0.044879417
218228_s_at	TNKS2	1.185092771	1784	10.38144256	0.04485426
224759_s_at	C12orf23	1.132098902	1785	10.38126388	0.044829132
225319_s_at	FAM104A	1.162314108	1786	10.37152405	0.045005599
235914_at	SYNPO	1.303147149	1787	10.36946136	0.045198657
225636_at	STAT2	1.22603486	1788	10.36840369	0.045173378
200658_s_at	PHB	-1.125058485	1789	10.36738615	0.045148127
204317_at	GTSE1	-1.234562607	1790	10.36556356	0.045122905
226021_at	RDH10	-1.114966219	1791	10.36495041	0.045097711
236417_at	MFN1	1.444930398	1792	10.36201171	0.045072545
204872_at	TLE4	1.599920257	1793	10.35784219	0.045309537
1558914_at	C1orf121	-1.108032348	1794	10.34981396	0.045429208
222427_s_at	LARS	-1.100378609	1795	10.3473501	0.0454039
213720_s_at	SMARCA4	-1.188383105	1796	10.34243761	0.045378619
201562_s_at	SORD	-1.316766922	1797	10.34103902	0.045353367
1555062_s_at	GTPBP3	-1.151887642	1798	10.34024825	0.045328142
209236_at	SLC23A2	-1.407368375	1799	10.33740604	0.045486381
224869_s_at	MRPS25	-1.117287138	1800	10.33660918	0.045461111
201057_s_at	GOLGB1	1.229438867	1801	10.33616172	0.045435869
218904_s_at	C9orf40	-1.21167266	1802	10.33075425	0.045410655
223218_s_at	NFKBIZ	1.623379162	1803	10.32993504	0.045601775
206857_s_at	FKBP1B	1.425037614	1804	10.32967566	0.045576497
218032_at	SNN	1.361314116	1805	10.32790342	0.045551247
232780_s_at	ZNF691	-1.074004472	1806	10.32109249	0.045526024
220944_at	PGLYRP4	-1.204972315	1807	10.31949369	0.045711123
225101_s_at	SNX14	1.154285418	1808	10.31772689	0.045685841
205081_at	CRIP1	1.677136369	1809	10.31689952	0.045660586
217905_at	C10orf119	-1.155886707	1810	10.31404154	0.045635359
200038_s_at	RPL17	-1.027401439	1811	10.30631084	0.045875207
226430_at	LOC253981	1.43893358	1812	10.30154734	0.04584989
221712_s_at	WDR74	-1.146312186	1813	10.29763476	0.045990072
203791_at	DMXL1	1.147107024	1814	10.28575643	0.046130099
216532_x_at	LOC643450	-1.115739322	1815	10.28517745	0.046104683
203090_at	SDF2	1.114966219	1816	10.27131122	0.046272026
201366_at	ANXA7	1.139183377	1817	10.27093205	0.04624656
223871_x_at	LOC727773	-1.170398641	1818	10.27041499	0.046221122
207332_s_at	TFRC	-1.124278924	1819	10.26204348	0.04633315
205187_at	SMAD5	1.188383105	1820	10.26047735	0.046307692
211749_s_at	VAMP3	1.139973273	1821	10.25691509	0.046425041
204279_at	PSMB9	1.210833084	1822	10.25660618	0.046399561
201013_s_at	PAICS	-1.107264584	1823	10.25459434	0.046374109
239825_at	ATF6	1.204137381	1824	10.24139653	0.046546053
202388_at	RGS2	2.393297926	1825	10.24116865	0.046520548
217099_s_at	GEMIN4	-1.178539408	1826	10.23932699	0.046708653
213115_at	ATG4A	1.149494848	1827	10.23887148	0.046683087
208407_s_at	CTNND1	1.346300069	1828	10.23511361	0.046657549
212343_at	YIPF6	1.122721422	1829	10.23242548	0.046632039
216488_s_at	ATP11A	-1.2397077	1830	10.23060298	0.046606557
236165_at	AA904502	1.564823563	1831	10.22963371	0.046766794

209154_at	TAX1BP3	1.252664439	1832	10.22945699	0.046741266
206060_s_at	PTPN22	1.171210181	1833	10.22739648	0.046715767
218332_at	BEX1	-1.137605228	1834	10.21992726	0.046832061
201582_at	SEC23B	1.22010051	1835	10.21968909	0.04680654
212483_at	NIPBL	-1.279872414	1836	10.21957326	0.046781046
207196_s_at	TNIP1	1.155886707	1837	10.21888635	0.04675558
1564208_x_at	FLJ35390	-1.138394029	1838	10.21782494	0.046730141
225847_at	AADACL1	1.336074078	1839	10.21412741	0.046704731
225322_s_at	C17orf70	-1.267512522	1840	10.21404141	0.046679348
225808_at	LOC124512	1.099616149	1841	10.21398137	0.046653992
227332_at	BF511170	-1.147107024	1842	10.21217225	0.046628664
222659_at	IPO11	-1.082224645	1843	10.20868105	0.046776994
205538_at	CORO2A	-1.278985581	1844	10.20466263	0.046751627
222552_at	GOLT1B	1.200803427	1845	10.20242297	0.046726287
227330_x_at	LOC389833	1.766630103	1846	10.20219324	0.046700975
223268_at	C11orf54	1.250062303	1847	10.20101739	0.04667569
205726_at	DIAPH2	1.236275261	1848	10.19819839	0.046883117
221945_at	FBXO41	-1.099616149	1849	10.19467877	0.046857761
224401_s_at	FCRL4	2.202283196	1850	10.1943346	0.046832432
212094_at	PEG10	-1.517819253	1851	10.19339649	0.046807131
212789_at	NCAPD3	-1.134455485	1852	10.19194977	0.046781857
214075_at	NENF	-1.074749173	1853	10.19024475	0.046756611
200677_at	PTTG1IP	1.23370717	1854	10.18836459	0.046952535
212450_at	KIAA0256	1.293248932	1855	10.18561077	0.046927224
214664_at	PAICS	-1.159095952	1856	10.18356906	0.04690194
215606_s_at	ERC1	1.308578071	1857	10.18171571	0.046876683
205085_at	ORC1L	-1.22603486	1858	10.18049922	0.046851453
202846_s_at	PIGC	1.188383105	1859	10.17547049	0.047014524
208981_at	PECAM1	1.427014506	1860	10.17286599	0.046989247
205120_s_at	SGCB	1.354724977	1861	10.1656838	0.047173563
217437_s_at	TACC1	1.151089491	1862	10.14930219	0.047502685
218578_at	CDC73	1.142346247	1863	10.14357422	0.047477187
1557684_at	ZNF286	-1.123499903	1864	10.13953811	0.047677039
224821_at	ABHD14B	-1.265756594	1865	10.1380061	0.047651475
216834_at	RGS1	1.508380077	1866	10.13681111	0.047625938
203268_s_at	DRG2	-1.085229372	1867	10.13546928	0.047600428
204153_s_at	MFNG	-1.183451022	1868	10.13389773	0.047574946
243629_x_at	MFI2	-1.120389214	1869	10.12212606	0.047774211
210075_at	2-Mar	1.41029796	1870	10.12179428	0.047748663
203029_s_at	PTPRN2	1.353786279	1871	10.1152929	0.047958311
209434_s_at	PPAT	-1.151089491	1872	10.11403377	0.047932692
226337_at	SCYL1BP1	1.240567298	1873	10.11363577	0.047907101
223094_s_at	ANKH	-1.352848231	1874	10.10943546	0.048052295
218139_s_at	C14orf108	1.121166078	1875	10.10932797	0.048026667
214696_at	MGC14376	1.386069886	1876	10.1049479	0.048001066
211036_x_at	ANAPC5	-1.074004472	1877	10.10189457	0.047975493
203614_at	UTP14C	1.115739322	1878	10.09911241	0.048157614
209339_at	SIAH2	1.394743666	1879	10.0962782	0.048131985
214670_at	ZKSCAN1	1.162314108	1880	10.09558095	0.048106383
1555065_x_at	USP6	1.137605228	1881	10.09417203	0.048080808
212898_at	KIAA0406	-1.121943481	1882	10.09074304	0.04805526

1559170_at	MGC26718	1.466116757	1883	10.0901748	0.04802974
200967_at	PPIB	1.150291893	1884	10.07584141	0.048370488
209206_at	SEC22B	1.114966219	1885	10.07430979	0.048344828
225926_at	VTI1B	1.151887642	1886	10.0708178	0.048319194
228921_at	SLC25A42	-1.180992661	1887	10.0653188	0.048516163
217944_at	POMGNT1	1.141554707	1888	10.06472319	0.048490466
208678_at	ATP6V1E1	1.108032348	1889	10.06257566	0.048464796
222275_at	AI039469	1.155886707	1890	10.05935464	0.048645503
227255_at	PDIK1L	1.20163605	1891	10.05544772	0.048619778
218344_s_at	RCOR3	1.063632673	1892	10.04886163	0.048736786
201971_s_at	ATP6V1A	1.264879542	1893	10.04348835	0.048711041
220651_s_at	MCM10	-1.203303026	1894	10.04346206	0.048685322
1555820_a_at	MKS1	-1.092020546	1895	10.03909522	0.048875989
203656_at	KIAA0274	1.22603486	1896	10.03876893	0.048850211
203605_at	SRP54	1.094293701	1897	10.03738003	0.04882446
208754_s_at	NAP1L1	-1.171210181	1898	10.03666451	0.048798736
214906_x_at	CG018	1.218410264	1899	10.03463653	0.048773038
224583_at	COTL1	-1.647182035	1900	10.03122463	0.048747368
203388_at	ARRB2	-1.254402205	1901	10.02677954	0.048874277
213511_s_at	MTMR1	1.136816973	1902	10.01036685	0.049074658
227093_at	USP36	-1.111108729	1903	10.0006629	0.049285339
226692_at	SERF2	1.149494848	1904	9.997762586	0.049448529
222512_at	NUB1	1.342572503	1905	9.991018937	0.049422572
213419_at	APBB2	-1.69466487	1906	9.990219737	0.049396642
226355_at	WDR51A	-1.131314463	1907	9.985331374	0.049559518
218197_s_at	OXR1	1.243149669	1908	9.983337276	0.049533543
225029_at	LOC550643	1.119612889	1909	9.969381275	0.04996857
202073_at	OPTN	1.346300069	1910	9.966339604	0.049942408
224926_at	EXOC4	1.161508732	1911	9.961301527	0.049916274
222422_s_at	NDFIP1	1.272794935	1912	9.960081928	0.049890167
221880_s_at	LOC400451	1.810012926	1915	9.953145558	0.049979112
218013_x_at	DCTN4	1.133669413	1916	9.951891809	0.049953027
218035_s_at	FLJ20273	1.205807828	1917	9.950980793	0.049926969
