# Supplementary material for: Transcriptional Dysregulation in NIPBL and Cohesin Mutant Human Cells
Source: PLoS Biol. 2009 May 26;7(5):e1000119. doi: 10.1371/journal.pbio.1000119 (PMC2680332; doi:10.1371/journal.pbio.1000119)
Supplement: Table S3 — 339 nonredundant genes represented by 420 probe sets (FDR<0.01) are differentially expressed in CdLS. (0.24 MB PDF) [file pbio.1000119.s007.pdf]

Table S3. 339 non-redundant genes represented by 420 probe sets (FDR < 0.01) are differentially expressed in Cornelia de Lange syndrome.

Probe set	Gene symbol	Fold change	Rank	F_Score	FDR
213918_s_at	NIPBL	-1.33	1	51.26	0
219911_s_at	SLCO4A1	-1.61	2	50.95	0
204394_at	SLC43A1	-1.7	3	49.9	0
203060_s_at	PAPSS2	-3.38	5	45.73	0
203333_at	KIFAP3	1.41	6	45.71	0
205204_at	NMB	-1.17	7	41.14	0
37549_g_at	PTHB1	1.33	8	40.55	0
226580_at	BRMS1L	1.52	9	40.35	0
216388_s_at	LTB4R	-1.33	10	40.14	0
244467_at	LOC440829	-2.65	11	39.86	0
215438_x_at	GSPT1	-1.17	12	39.34	0
229332_at	GLOXD1	-2.36	13	39.24	0
204866_at	PHF16	3.85	14	37.31	0
218491_s_at	THYN1	-1.22	15	37.14	0
225914_s_at	CAB39L	1.77	16	35.96	0.000625
205352_at	SERPINI1	1.76	17	35.89	0.000588235
226611_s_at	PRR6	-1.83	18	35.84	0.000555556
225347_at	ARL8A	1.28	19	35.01	0.000526316
217995_at	SQRDL	1.36	21	34.37	0.00047619
203836_s_at	MAP3K5	1.55	22	33.89	0.000454545
207937_x_at	FGFR1	-1.22	23	33.66	0.000434783
212240_s_at	PIK3R1	1.36	24	33.42	0.000416667
231843_at	DDX55	-1.25	25	33.31	0.0004
209861_s_at	METAP2	-1.19	26	33.14	0.000769231
224648_at	GPBP1	1.25	27	32.49	0.001481481
205685_at	CD86	1.66	28	32.47	0.001428571
225639_at	SKAP2	1.44	29	32.4	0.00137931
225403_at	C9orf23	-1.23	30	32.1	0.001666667
225564_at	SPATA13	1.7	31	31.9	0.001612903
201522_x_at	SNRPN	1.25	32	31.58	0.001875
201952_at	ALCAM	1.37	33	31.41	0.002121212
209079_x_at	PCDHGC3	-2.06	35	31.08	0.002
201889_at	FAM3C	1.67	36	30.83	0.001944444
212335_at	GNS	1.36	37	30.6	0.001891892
218993_at	RNMTL1	-1.14	38	30.36	0.001842105
201658_at	ARL1	1.21	39	30.29	0.001794872
204601_at	N4BP1	1.29	40	30.27	0.00175
208910_s_at	C1QBP	-1.26	41	30.18	0.001707317
225777_at	C9orf140	-1.6	42	30.09	0.001666667
221752_at	SSH1	1.48	43	29.64	0.001860465
226991_at	NFATC2	-2.11	44	29.57	0.001818182
200903_s_at	AHCY	-1.23	45	29.48	0.001777778
218883_s_at	MLF1IP	-1.33	47	29.23	0.001702128
228764_s_at	CHMP4A	1.21	48	29.22	0.001666667
221808_at	RAB9	1.56	49	29.08	0.001836735
212959_s_at	GNPTAB	1.47	50	29.07	0.0018

218104_at	TEX10	-1.19	52	28.99	0.001730769
219734_at	SIDT1	1.49	54	28.86	0.001666667
229828_at	AL044007	1.25	55	28.74	0.002
218017_s_at	HGSNAT	1.48	57	28.49	0.002105263
207966_s_at	GLG1	1.27	58	28.3	0.002068966
200945_s_at	SEC31A	1.18	59	28.29	0.002033898
1569346_a_at	P2RX1	1.28	60	28.22	0.002
201770_at	SNRPA	-1.17	61	28.22	0.001967213
222692_s_at	FNDC3B	1.89	62	28.22	0.001935484
220768_s_at	CSNK1G3	1.25	64	28.13	0.001875
229817_at	ZNF608	2.19	65	28.11	0.001846154
218512_at	WDR12	-1.19	66	28.01	0.001969697
201847_at	LIPA	1.35	67	27.79	0.001940299
212291_at	HIPK1	1.32	68	27.7	0.001911765
227279_at	TCEAL3	1.34	70	27.65	0.001857143
228977_at	LOC729680	-1.57	71	27.59	0.001830986
208953_at	LARP5	1.14	72	27.55	0.001944444
225924_at	KIAA1450	2.62	73	27.48	0.001917808
210788_s_at	DHRS7	1.35	75	27.26	0.002
201813_s_at	TBC1D5	1.31	76	27.2	0.001973684
219274_at	TSPAN12	2.97	79	27.07	0.001898734
227525_at	GLCCI1	1.47	80	27.04	0.001875
208093_s_at	NDEL1	1.18	81	26.81	0.001851852
203867_s_at	NLE1	-1.24	85	26.56	0.002235294
202975_s_at	RHOBTB3	2.53	88	26.26	0.002386364
208660_at	CS	-1.13	89	26.26	0.002359551
1564907_s_at	MATR3	-1.38	90	26.15	0.002555556
201614_s_at	RUVBL1	-1.22	91	26.12	0.002527473
233759_s_at	SMEK2	1.13	92	26.09	0.0025
216020_at	IFIH1	1.49	93	25.99	0.002473118
212846_at	KIAA0179	-1.27	94	25.96	0.002446809
218909_at	RPS6KC1	1.24	95	25.92	0.002421053
202559_x_at	C1orf77	-1.09	96	25.88	0.002395833
224714_at	MKI67IP	-1.14	97	25.85	0.002371134
223917_s_at	SLC39A3	-1.34	98	25.78	0.002346939
242714_at	AW500340	3.08	99	25.73	0.002323232
241937_s_at	WDR4	-1.37	100	25.71	0.0024
210115_at	RPL39L	-1.31	101	25.66	0.002376238
218081_at	C20orf27	-1.31	102	25.65	0.002352941
224046_s_at	PDE7A	-1.36	104	25.39	0.002692308
201196_s_at	AMD1	-1.21	105	25.21	0.003142857
203882_at	ISGF3G	1.3	106	25.15	0.003113208
235347_at	LRCH3	1.29	108	25	0.003240741
209917_s_at	TP53AP1	1.37	109	24.99	0.003302752
212733_at	KIAA0226	1.24	110	24.84	0.003818182
213049_at	GARNL1	1.29	111	24.82	0.003783784
222024_s_at	AKAP13	1.3	112	24.69	0.003928571
210224_at	MR1	1.28	113	24.68	0.003893805
220762_s_at	GNB1L	-1.16	114	24.67	0.003859649
201328_at	ETS2	-1.28	115	24.63	0.003826087
213194_at	ROBO1	4.61	116	24.59	0.004137931
213669_at	FCHO1	-1.35	117	24.58	0.004102564

212355_at	KIAA0323	1.27	118	24.49	0.004237288
203758_at	CTSO	1.42	119	24.42	0.004285714
205483_s_at	ISG15	1.83	120	24.42	0.00425
228442_at	AI770171	-2.06	124	24.19	0.004435484
205733_at	BLM	-1.27	125	24.14	0.0044
209036_s_at	MDH2	-1.08	126	23.98	0.004603175
202085_at	TJP2	1.34	128	23.91	0.0046875
212742_at	ZNF364	1.15	129	23.86	0.004728682
208759_at	IKBKB	1.22	130	23.85	0.004692308
203173_s_at	MGC16824	1.17	131	23.84	0.004656489
202304_at	FNDC3A	1.34	132	23.82	0.00469697
217737_x_at	C20orf43	1.11	133	23.8	0.004661654
212441_at	KIAA0232	1.23	135	23.76	0.004666667
206037_at	CCBL1	-1.15	136	23.72	0.004632353
218058_at	CXXC1	-1.42	137	23.65	0.00459854
203291_at	CNOT4	1.16	138	23.53	0.004710145
208089_s_at	TDRD3	1.16	139	23.5	0.004748201
209664_x_at	NFATC1	-1.5	140	23.5	0.004714286
213238_at	ATP10D	1.71	142	23.44	0.00471831
235583_at	ILDR1	1.68	143	23.32	0.004825175
201563_at	SORD	-1.37	144	23.29	0.004861111
220588_at	BCAS4	-1.43	145	23.27	0.004827586
218085_at	CHMP5	1.26	146	23.26	0.004794521
212625_at	STX10	-1.25	147	23.21	0.004897959
226249_at	SNX30	-1.47	148	23.2	0.005
208680_at	PRDX1	-1.19	149	23.18	0.004966443
236835_at	LOC645431	-1.64	151	23.15	0.004900662
203384_s_at	GOLGA1	1.24	152	23.09	0.005065789
221920_s_at	SLC25A37	-1.65	153	23.08	0.005098039
235509_at	C8orf38	-1.27	154	23	0.00525974
224460_s_at	L2HGDH	-1.3	155	23	0.005225806
202180_s_at	MVP	1.57	156	22.88	0.00525641
212543_at	AIM1	1.77	157	22.81	0.005414013
209679_s_at	LOC57228	-1.92	159	22.74	0.005345912
225858_s_at	BIRC4	1.28	160	22.73	0.0053125
223134_at	BBX	1.29	162	22.68	0.00537037
203724_s_at	RUFY3	1.78	163	22.68	0.005337423
231517_at	ZYG11A	-1.88	164	22.68	0.005304878
1554015_a_at	CHD2	1.21	165	22.66	0.005272727
224610_at	SNHG1	-1.19	166	22.53	0.005481928
200701_at	NPC2	1.26	167	22.47	0.005449102
202468_s_at	CTNNAL1	-1.37	169	22.44	0.005443787
226196_s_at	C14orf179	1.31	170	22.43	0.005411765
201930_at	MCM6	-1.27	171	22.42	0.005380117
203885_at	RAB21	1.22	172	22.42	0.005348837
228252_at	PIF1	-1.26	173	22.39	0.005433526
219110_at	NOLA1	-1.19	174	22.39	0.005402299
222514_at	RRAGC	1.23	175	22.38	0.005371429
203227_s_at	TSPAN31	1.34	177	22.27	0.005367232
217733_s_at	TMSB10	1.18	178	22.26	0.005393258
221953_s_at	MMP24	-1.12	179	22.18	0.005363128
204308_s_at	KIAA0329	1.59	180	22.18	0.005333333

202149_at	NEDD9	1.69	181	22.14	0.005359116
212310_at	MIA3	1.27	182	22.13	0.00532967
226008_at	NDNL2	1.56	183	22.11	0.005409836
229270_x_at	LOC646044	-1.38	186	22.03	0.005376344
238510_at	ZNF720	1.24	187	22.02	0.00540107
209447_at	SYNE1	1.5	188	21.99	0.00537234
225074_at	RAB2B	1.27	189	21.97	0.005396825
214059_at	IFI44	1.72	190	21.96	0.005421053
207571_x_at	C1orf38	1.68	191	21.94	0.00539267
1555832_s_at	KLF6	1.46	192	21.9	0.005572917
217043_s_at	SYT7	1.32	193	21.86	0.005751295
231866_at	LNPEP	1.48	194	21.82	0.005721649
202144_s_at	ADSL	-1.12	195	21.82	0.005692308
218324_s_at	SPATS2	1.2	196	21.79	0.005765306
226713_at	CCDC50	1.55	198	21.75	0.005757576
202395_at	NSF	1.36	200	21.73	0.00575
214299_at	TOP3A	-1.16	201	21.72	0.005721393
213271_s_at	DOPEY1	1.36	202	21.62	0.005891089
208920_at	SRI	1.65	207	21.47	0.005845411
224326_s_at	PCGF6	-1.1	210	21.28	0.006285714
201393_s_at	IGF2R	1.29	211	21.27	0.006255924
1558381_a_at	GAPDHS	-1.17	212	21.25	0.006226415
203159_at	GLS	1.32	213	21.23	0.006197183
210044_s_at	LYL1	-1.56	214	21.23	0.006168224
219648_at	MREG	1.35	215	21.2	0.006186047
217743_s_at	TMEM30A	1.18	216	21.17	0.006203704
201457_x_at	BUB3	-1.16	217	21.16	0.006175115
209090_s_at	SH3GLB1	1.28	218	21.11	0.00646789
201989_s_at	CREBL2	1.25	219	21.06	0.006621005
208030_s_at	ADD1	1.26	220	21.03	0.006636364
212807_s_at	SORT1	1.86	221	21.03	0.006606335
212467_at	DNAJC13	1.09	222	21.03	0.006576577
204286_s_at	PMAIP1	1.4	224	20.98	0.0065625
221519_at	FBXW4	1.18	227	20.92	0.00660793
224722_at	MIB1	1.39	228	20.91	0.006578947
222613_at	C12orf4	1.14	230	20.89	0.006521739
213132_s_at	MCAT	-1.18	231	20.89	0.006493506
206074_s_at	HMGA1	-1.25	232	20.83	0.006508621
205403_at	IL1R2	3.17	233	20.82	0.006480687
202121_s_at	CHMP2A	1.15	234	20.78	0.006581197
217854_s_at	POLR2E	-1.13	235	20.77	0.006553191
224502_s_at	KIAA1191	1.26	236	20.71	0.006737288
225957_at	LOC153222	1.41	237	20.7	0.006793249
201391_at	TRAP1	-1.26	240	20.65	0.006833333
221156_x_at	CCPG1	1.55	242	20.59	0.006859504
235830_at	NT5DC1	1.3	244	20.53	0.006885246
220507_s_at	UPB1	1.39	245	20.52	0.006857143
203247_s_at	ZNF24	1.14	246	20.48	0.006910569
205264_at	CD3EAP	-1.37	247	20.47	0.006923077
227701_at	C10orf118	1.33	249	20.46	0.006907631
224957_at	LOC497661	1.18	251	20.44	0.00685259
222230_s_at	ACTR10	1.1	252	20.4	0.006944444

212006_at	UBXD2	1.15	253	20.39	0.006916996
203206_at	FAM53B	-1.36	254	20.33	0.007047244
209750_at	NR1D2	1.33	256	20.25	0.007070313
200083_at	USP22	-1.09	257	20.22	0.007120623
218132_s_at	TSEN34	1.15	258	20.21	0.007093023
202962_at	KIF13B	1.29	260	20.2	0.007038462
222498_at	AZI2	1.21	261	20.19	0.007011494
222408_s_at	YPEL5	1.53	262	20.19	0.006984733
218141_at	UBE2O	-1.17	263	20.18	0.006996198
203596_s_at	IFIT5	1.48	264	20.15	0.007007576
204780_s_at	FAS	1.42	265	20.11	0.007018868
243745_at	AP1S2	-1.31	266	20.1	0.006992481
219901_at	FGD6	-2.16	267	20.1	0.006966292
219458_s_at	NSUN3	1.14	268	20.09	0.006940299
205105_at	MAN2A1	2.13	270	20.07	0.006925926
238012_at	DPP7	-1.3	271	20.06	0.006900369
200673_at	LAPTM4A	1.2	272	20.05	0.006875
223422_s_at	ARHGAP24	2.89	274	20	0.00689781
236080_at	BE276063	-1.15	276	19.98	0.006847826
229146_at	C7orf31	1.24	277	19.94	0.006895307
212282_at	TMEM97	-1.32	279	19.87	0.006989247
211623_s_at	FBL	-1.13	280	19.85	0.007
204573_at	CROT	1.48	281	19.84	0.007010676
208273_at	ZNF695	-1.79	282	19.84	0.006985816
204198_s_at	RUNX3	1.25	284	19.83	0.00693662
201133_s_at	PJA2	1.13	285	19.79	0.007017544
218020_s_at	ZFAND3	1.25	286	19.79	0.006993007
223177_at	GLI3	1.21	287	19.78	0.006968641
214749_s_at	ARMCX6	1.13	289	19.76	0.007024221
231927_at	ATF6	1.26	291	19.7	0.007113402
202318_s_at	SENP6	1.14	292	19.67	0.007123288
201892_s_at	IMPDH2	-1.17	293	19.6	0.007303754
222281_s_at	AW517716	2.78	294	19.6	0.007278912
212400_at	FAM102A	-1.48	295	19.59	0.007254237
226682_at	LOC283666	4.01	296	19.58	0.00722973
205920_at	SLC6A6	-1.55	297	19.58	0.007205387
212048_s_at	YARS	-1.16	298	19.58	0.007181208
222360_at	DPH5	-1.17	299	19.57	0.007157191
225522_at	AAK1	1.31	300	19.56	0.007166667
204033_at	TRIP13	-1.27	301	19.55	0.00717608
213245_at	ADCY1	-2.76	302	19.51	0.007284768
227697_at	SOCS3	2.78	306	19.42	0.00748366
226391_at	NDUFB2	1.27	308	19.34	0.007694805
219863_at	HERC5	1.43	309	19.34	0.007669903
202284_s_at	CDKN1A	1.32	310	19.32	0.007677419
204510_at	CDC7	-1.28	311	19.3	0.007717042
222235_s_at	GALNACT-2	1.34	312	19.25	0.007948718
213581_at	PDCD2	-1.18	313	19.24	0.007923323
226799_at	AK026881	-1.79	314	19.23	0.007929936
222781_s_at	C9orf40	-1.28	315	19.23	0.007904762
201272_at	AKR1B1	-1.11	316	19.2	0.008006329
207826_s_at	ID3	-1.77	317	19.19	0.008012618

209421_at	MSH2	-1.28	319	19.15	0.008056426
224404_s_at	FCRL5	2.72	320	19.15	0.00803125
206106_at	MAPK12	-1.37	321	19.15	0.008006231
212341_at	YIPF6	1.19	322	19.15	0.007981366
218048_at	COMMD3	1.26	323	19.13	0.007987616
1554806_a_at	FBXO8	1.22	324	19.1	0.00808642
228205_at	TKT	-1.14	325	19.08	0.008061538
227968_at	PDDC1	-1.17	326	19.08	0.00803681
205641_s_at	TRADD	1.3	327	19.07	0.008042813
223209_s_at	SELS	1.43	328	19.04	0.008109756
226267_at	JDP2	-1.52	331	19	0.008277946
213521_at	PTPN18	-1.38	332	18.99	0.008253012
223018_at	NOB1	-1.12	333	18.98	0.008228228
211729_x_at	BLVRA	1.52	334	18.91	0.008413174
238520_at	TRERF1	-2.35	336	18.88	0.008422619
212498_at	AF056433	1.21	337	18.83	0.008694362
218590_at	PEO1	-1.14	338	18.83	0.008668639
202788_at	MAPKAPK3	-1.31	339	18.81	0.008672566
201641_at	BST2	1.21	340	18.77	0.008735294
223738_s_at	PGM2	-1.09	342	18.75	0.00880117
201193_at	IDH1	1.23	343	18.75	0.00877551
213073_at	ZFYVE26	1.3	344	18.75	0.00875
208783_s_at	CD46	1.18	345	18.74	0.008724638
206175_x_at	ZNF222	1.19	346	18.73	0.008757225
201851_at	SH3GL1	1.15	347	18.73	0.008731988
201710_at	MYBL2	-1.25	348	18.72	0.008735632
204128_s_at	RFC3	-1.26	350	18.71	0.008742857
223892_s_at	TMBIM4	1.18	351	18.7	0.008774929
212036_s_at	PNN	-1.12	352	18.7	0.00875
203097_s_at	RAPGEF2	1.74	354	18.69	0.008728814
217974_at	TM7SF3	1.43	356	18.67	0.008707865
243521_at	AW590862	1.16	357	18.67	0.008683473
222620_s_at	DNAJC1	1.28	358	18.65	0.008715084
201972_at	ATP6V1A	1.26	359	18.63	0.008857939
216251_s_at	TTLL12	-1.28	360	18.62	0.008916667
201311_s_at	SH3BGRL	1.15	361	18.62	0.008891967
226122_at	PLEKHG1	1.71	362	18.61	0.008922652
226262_at	AA534526	-1.22	363	18.6	0.008898072
228478_at	AA889954	1.27	364	18.59	0.008873626
207098_s_at	MFN1	1.37	365	18.57	0.008931507
201999_s_at	DYNLT1	1.35	367	18.55	0.008991826
224696_s_at	WDR22	1.13	368	18.52	0.009103261
221746_at	UBL4A	-1.14	369	18.52	0.009078591
207339_s_at	LTB	-2.15	370	18.51	0.009135135
224468_s_at	C19orf48	-1.19	371	18.46	0.00916442
235812_at	C16orf69	1.15	372	18.45	0.009166667
221918_at	PCTK2	1.35	373	18.42	0.009276139
206055_s_at	SNRPA1	-1.17	374	18.41	0.009304813
202078_at	COPS3	-1.1	375	18.41	0.00928
229350_x_at	PARP10	1.23	376	18.32	0.009574468
222401_s_at	TMEM50A	1.14	377	18.31	0.009549072
226440_at	DUSP22	1.29	378	18.29	0.009603175

244422_at	AI494573	-1.42	379	18.28	0.009577836
203955_at	KIAA0649	1.38	382	18.26	0.009581152
215930_s_at	CTAGE5	1.32	383	18.25	0.009634465
207621_s_at	PEMT	-1.13	384	18.24	0.009635417
222914_s_at	TMEM121	-1.15	386	18.23	0.009585492
202534_x_at	DHFR	-1.3	387	18.22	0.009638243
214114_x_at	FASTK	1.12	388	18.2	0.009690722
209004_s_at	FBXL5	1.21	389	18.2	0.00966581
201075_s_at	SMARCC1	-1.24	392	18.15	0.009719388
226333_at	AV700030	-2.28	393	18.13	0.009745547
218671_s_at	ATPIF1	-1.11	395	18.13	0.009696203
209418_s_at	THOC5	-1.09	396	18.13	0.009671717
231690_at	AI962352	1.45	398	18.07	0.009773869
225458_at	LOC25845	-1.2	399	18.06	0.009749373
218161_s_at	CLN6	-1.26	400	18.05	0.0098
209900_s_at	SLC16A1	-1.23	401	18.04	0.009875312
203732_at	TRIP4	1.16	402	18.04	0.009850746
202911_at	MSH6	-1.19	403	18.04	0.009826303
218581_at	ABHD4	1.33	404	18.04	0.00980198
238190_at	TUFM	-1.11	405	18.03	0.009802469
209194_at	CETN2	1.13	406	18.03	0.009778325
221788_at	PGM3	1.23	407	18.01	0.00980344
227056_at	KIAA0141	1.26	408	18	0.009803922
227802_at	AI075999	1.69	410	17.96	0.009829268
201968_s_at	PGM1	1.2	411	17.95	0.009854015
226809_at	FLJ30428	-1.57	412	17.92	0.009951456
203194_s_at	NUP98	-1.17	413	17.92	0.009927361
212150_at	KIAA0143	1.19	414	17.91	0.009951691
213134_x_at	BTG3	1.25	415	17.91	0.009927711
238695_s_at	RAB39B	1.27	416	17.91	0.009903846
1559220_at	BG025779	-1.14	417	17.9	0.009880096
223217_s_at	NFKBIZ	1.64	419	17.89	0.009856802
200977_s_at	TAX1BP1	1.27	420	17.87	0.00997619
