# Supplementary material for: Transcriptional Dysregulation in NIPBL and Cohesin Mutant Human Cells
Source: PLoS Biol. 2009 May 26;7(5):e1000119. doi: 10.1371/journal.pbio.1000119 (PMC2680332; doi:10.1371/journal.pbio.1000119)
Supplement: Table S4 — Evaluation of Leave-One-Out cross-validation for the 33 samples in the training set. Two healthy controls and one proband were misclassified. (0.22 MB PDF) [file pbio.1000119.s008.pdf]

Table S4. Evaluation of Leave-One-Out cross-validation for the 33 samples in the training set. Two healthy controls and 1 proband were misclassified.

		True conditions				
		Proband	Control	Total	*PPV	*NPV
Test results	Proband	15	2	17	15/17=0.88	
	Control	1	15	16		15/16=0.94
	Total	16	17	33		
	Sensitivity	15/16=0.94				
	Specificity		15/17=0.88			

*PPV: positive predictive value; NPV: negative predictive value
