# Supplementary material for: Transcriptional Dysregulation in NIPBL and Cohesin Mutant Human Cells
Source: PLoS Biol. 2009 May 26;7(5):e1000119. doi: 10.1371/journal.pbio.1000119 (PMC2680332; doi:10.1371/journal.pbio.1000119)
Supplement: Table S5 — Five functional independent gene clusters identified among the 339 genes (FDR<0.01) using GSEA online program and R code. (0.24 MB PDF) [file pbio.1000119.s009.pdf]

Table S5. Five functional independent gene clusters identified among the 339 genes (FDR < 0.01) using GSEA online program and R code.

Probe set	Cluster	Gene symbol	Fold change	F_Score	Rank	p_Value	FDR
205204_at	1	NMB	-1.17	41.14	7	3.8E-07	0
216388_s_at	1	LTB4R	-1.33	40.14	10	4.73E-07	0
244467_at	1	LOC440829	-2.65	39.86	11	5.04E-07	0
207937_x_at	1	FGFR1	-1.22	33.66	23	2.16E-06	0.000435
225403_at	1	C9orf23	-1.23	32.1	30	3.19E-06	0.001667
200903_s_at	1	AHCY	-1.23	29.48	45	6.26E-06	0.001778
226991_at	1	NFATC2	-2.11	29.57	44	6.1E-06	0.001818
209079_x_at	1	PCDHGC3	-2.06	31.08	35	4.12E-06	0.002
210115_at	1	RPL39L	-1.31	25.66	101	1.78E-05	0.002376
212846_at	1	KIAA0179	-1.27	25.96	94	1.63E-05	0.002447
228442_at	1	AI770171	-2.06	24.19	124	2.71E-05	0.004435
220588_at	1	BCAS4	-1.43	23.27	145	3.55E-05	0.004828
201563_at	1	SORD	-1.37	23.29	144	3.53E-05	0.004861
236835_at	1	LOC645431	-1.64	23.15	151	3.68E-05	0.004901
208680_at	1	PRDX1	-1.19	23.18	149	3.65E-05	0.004966
235509_at	1	C8orf38	-1.27	23	154	3.84E-05	0.00526
231517_at	1	ZYG11A	-1.88	22.68	164	4.24E-05	0.005305
209679_s_at	1	LOC57228	-1.92	22.74	159	4.15E-05	0.005346
229270_x_at	1	LOC646044	-1.38	22.03	186	5.15E-05	0.005376
219901_at	1	FGD6	-2.16	20.1	267	9.37E-05	0.006966
212282_at	1	TMEM97	-1.32	19.87	279	0.000101	0.006989
203206_at	1	FAM53B	-1.36	20.33	254	8.72E-05	0.007047
222360_at	1	DPH5	-1.17	19.57	299	0.000111	0.007157
212400_at	1	FAM102A	-1.48	19.59	295	0.00011	0.007254
213245_at	1	ADCY1	-2.76	19.51	302	0.000113	0.007285
201892_s_at	1	IMPDH2	-1.17	19.6	293	0.00011	0.007304
226799_at	1	AK026881	-1.79	19.23	314	0.000124	0.00793
201272_at	1	AKR1B1	-1.11	19.2	316	0.000125	0.008006
207826_s_at	1	ID3	-1.77	19.19	317	0.000125	0.008013
223018_at	1	NOB1	-1.12	18.98	333	0.000134	0.008228
226267_at	1	JDP2	-1.52	19	331	0.000134	0.008278
238520_at	1	TRERF1	-2.35	18.88	336	0.000139	0.008423
202788_at	1	MAPKAPK3	-1.31	18.81	339	0.000142	0.008673
223738_s_at	1	PGM2	-1.09	18.75	342	0.000144	0.008801
224468_s_at	1	C19orf48	-1.19	18.46	371	0.000159	0.009164
222914_s_at	1	TMEM121	-1.15	18.23	386	0.000171	0.009585
207621_s_at	1	PEMT	-1.13	18.24	384	0.000171	0.009635
218671_s_at	1	ATPIF1	-1.11	18.13	395	0.000177	0.009696
226333_at	1	AV700030	-2.28	18.13	393	0.000177	0.009746
226809_at	1	FLJ30428	-1.57	17.92	412	0.00019	0.009951
203333_at	2	KIFAP3	1.41	45.71	6	1.44E-07	0
37549_g_at	2	PTHB1	1.33	40.55	8	4.33E-07	0
226580_at	2	BRMS1L	1.52	40.35	9	4.51E-07	0

203836_s_at	2	MAP3K5	1.55	33.89	22	2.04E-06	0.000455
217995_at	2	SQRDL	1.36	34.37	21	1.81E-06	0.000476
205352_at	2	SERPINI1	1.76	35.89	17	1.26E-06	0.000588
225914_s_at	2	CAB39L	1.77	35.96	16	1.24E-06	0.000625
225639_at	2	SKAP2	1.44	32.4	29	2.95E-06	0.001379
205685_at	2	CD86	1.66	32.47	28	2.9E-06	0.001429
224648_at	2	GPBP1	1.25	32.49	27	2.88E-06	0.001481
228764_s_at	2	CHMP4A	1.21	29.22	48	6.7E-06	0.001667
219734_at	2	SIDT1	1.49	28.86	54	7.37E-06	0.001667
204601_at	2	N4BP1	1.29	30.27	40	5.09E-06	0.00175
201658_at	2	ARL1	1.21	30.29	39	5.06E-06	0.001795
212959_s_at	2	GNPTAB	1.47	29.07	50	6.98E-06	0.0018
208093_s_at	2	NDEL1	1.18	26.81	81	1.29E-05	0.001852
227279_at	2	TCEAL3	1.34	27.65	70	1.02E-05	0.001857
201522_x_at	2	SNRPN	1.25	31.58	32	3.63E-06	0.001875
227525_at	2	GLCCI1	1.47	27.04	80	1.21E-05	0.001875
212335_at	2	GNS	1.36	30.6	37	4.67E-06	0.001892
212291_at	2	HIPK1	1.32	27.7	68	1.01E-05	0.001912
225924_at	2	KIAA1450	2.62	27.48	73	1.07E-05	0.001918
222692_s_at	2	FNDC3B	1.89	28.22	62	8.76E-06	0.001935
201889_at	2	FAM3C	1.67	30.83	36	4.4E-06	0.001944
208953_at	2	LARP5	1.14	27.55	72	1.05E-05	0.001944
201813_s_at	2	TBC1D5	1.31	27.2	76	1.16E-05	0.001974
1569346_a_at	2	P2RX1	1.28	28.22	60	8.75E-06	0.002
200945_s_at	2	SEC31A	1.18	28.29	59	8.59E-06	0.002034
207966_s_at	2	GLG1	1.27	28.3	58	8.56E-06	0.002069
201952_at	2	ALCAM	1.37	31.41	33	3.79E-06	0.002121
203882_at	2	ISGF3G	1.3	25.15	106	2.05E-05	0.003113
235347_at	2	LRCH3	1.29	25	108	2.14E-05	0.003241
213049_at	2	GARNL1	1.29	24.82	111	2.26E-05	0.003784
222024_s_at	2	AKAP13	1.3	24.69	112	2.34E-05	0.003929
212355_at	2	KIAA0323	1.27	24.49	118	2.48E-05	0.004237
205483_s_at	2	ISG15	1.83	24.42	120	2.54E-05	0.00425
203173_s_at	2	MGC16824	1.17	23.84	131	3E-05	0.004656
217737_x_at	2	C20orf43	1.11	23.8	133	3.04E-05	0.004662
208759_at	2	IKBKB	1.22	23.85	130	2.99E-05	0.004692
202304_at	2	FNDC3A	1.34	23.82	132	3.02E-05	0.004697
203291_at	2	CNOT4	1.16	23.53	138	3.29E-05	0.00471
213238_at	2	ATP10D	1.71	23.44	142	3.38E-05	0.004718
212742_at	2	ZNF364	1.15	23.86	129	2.99E-05	0.004729
208089_s_at	2	TDRD3	1.16	23.5	139	3.32E-05	0.004748
218085_at	2	CHMP5	1.26	23.26	146	3.56E-05	0.004795
235583_at	2	ILDR1	1.68	23.32	143	3.5E-05	0.004825
202180_s_at	2	MVP	1.57	22.88	156	3.99E-05	0.005256
1554015_a_at	2	CHD2	1.21	22.66	165	4.26E-05	0.005273
225858_s_at	2	BIRC4	1.28	22.73	160	4.17E-05	0.005313
212310_at	2	MIA3	1.27	22.13	182	4.99E-05	0.00533
204308_s_at	2	KIAA0329	1.59	22.18	180	4.93E-05	0.005333
203724_s_at	2	RUFY3	1.78	22.68	163	4.24E-05	0.005337
209447_at	2	SYNE1	1.5	21.99	188	5.22E-05	0.005372
217733_s_at	2	TMSB10	1.18	22.26	178	4.81E-05	0.005393

238510_at	2	ZNF720	1.24	22.02	187	5.17E-05	0.005401
226008_at	2	NDNL2	1.56	22.11	183	5.03E-05	0.00541
214059_at	2	IFI44	1.72	21.96	190	5.26E-05	0.005421
202395_at	2	NSF	1.36	21.73	200	5.65E-05	0.00575
226713_at	2	CCDC50	1.55	21.75	198	5.62E-05	0.005758
218324_s_at	2	SPATS2	1.2	21.79	196	5.53E-05	0.005765
208920_at	2	SRI	1.65	21.47	207	6.12E-05	0.005845
213271_s_at	2	DOPEY1	1.36	21.62	202	5.83E-05	0.005891
203159_at	2	GLS	1.32	21.23	213	6.58E-05	0.006197
222613_at	2	C12orf4	1.14	20.89	230	7.31E-05	0.006522
212467_at	2	DNAJC13	1.09	21.03	222	7.01E-05	0.006577
212807_s_at	2	SORT1	1.86	21.03	221	7E-05	0.006606
221519_at	2	FBXW4	1.18	20.92	227	7.24E-05	0.006608
201989_s_at	2	CREBL2	1.25	21.06	219	6.94E-05	0.006621
208030_s_at	2	ADD1	1.26	21.03	220	6.99E-05	0.006636
221156_x_at	2	CCPG1	1.55	20.59	242	8.03E-05	0.00686
235830_at	2	NT5DC1	1.3	20.53	244	8.18E-05	0.006885
227701_at	2	C10orf118	1.33	20.46	249	8.37E-05	0.006908
203247_s_at	2	ZNF24	1.14	20.48	246	8.31E-05	0.006911
212006_at	2	UBXD2	1.15	20.39	253	8.56E-05	0.006917
205105_at	2	MAN2A1	2.13	20.07	270	9.47E-05	0.006926
219458_s_at	2	NSUN3	1.14	20.09	268	9.4E-05	0.00694
222230_s_at	2	ACTR10	1.1	20.4	252	8.51E-05	0.006944
223177_at	2	GLI3	1.21	19.78	287	0.000104	0.006969
203596_s_at	2	IFIT5	1.48	20.15	264	9.22E-05	0.007008
204573_at	2	CROT	1.48	19.84	281	0.000102	0.007011
214749_s_at	2	ARMCX6	1.13	19.76	289	0.000104	0.007024
202962_at	2	KIF13B	1.29	20.2	260	9.08E-05	0.007038
209750_at	2	NR1D2	1.33	20.25	256	8.94E-05	0.00707
226682_at	2	LOC283666	4.01	19.58	296	0.00011	0.00723
222281_s_at	2	AW517716	2.78	19.6	294	0.00011	0.007279
227697_at	2	SOCS3	2.78	19.42	306	0.000117	0.007484
202284_s_at	2	CDKN1A	1.32	19.32	310	0.00012	0.007677
226391_at	2	NDUFB2	1.27	19.34	308	0.000119	0.007695
222235_s_at	2	GALNACT-2	1.34	19.25	312	0.000123	0.007949
212341_at	2	YIPF6	1.19	19.15	322	0.000127	0.007981
218048_at	2	COMMD3	1.26	19.13	323	0.000128	0.007988
224404_s_at	2	FCRL5	2.72	19.15	320	0.000127	0.008031
223209_s_at	2	SELS	1.43	19.04	328	0.000131	0.00811
211729_x_at	2	BLVRA	1.52	18.91	334	0.000137	0.008413
243521_at	2	AW590862	1.16	18.67	357	0.000149	0.008683
222620_s_at	2	DNAJC1	1.28	18.65	358	0.000149	0.008715
203097_s_at	2	RAPGEF2	1.74	18.69	354	0.000148	0.008729
201641_at	2	BST2	1.21	18.77	340	0.000144	0.008735
206175_x_at	2	ZNF222	1.19	18.73	346	0.000145	0.008757
223892_s_at	2	TMBIM4	1.18	18.7	351	0.000147	0.008775
201972_at	2	ATP6V1A	1.26	18.63	359	0.00015	0.008858
224696_s_at	2	WDR22	1.13	18.52	368	0.000156	0.009103
235812_at	2	C16orf69	1.15	18.45	372	0.000159	0.009167
229350_x_at	2	PARP10	1.23	18.32	376	0.000166	0.009574
214114_x_at	2	FASTK	1.12	18.2	388	0.000173	0.009691

209194_at	2	CETN2	1.13	18.03	406	0.000183	0.009778
218581_at	2	ABHD4	1.33	18.04	404	0.000183	0.009802
221788_at	2	PGM3	1.23	18.01	407	0.000184	0.009803
227802_at	2	AI075999	1.69	17.96	410	0.000187	0.009829
223217_s_at	2	NFKBIZ	1.64	17.89	419	0.000192	0.009857
238695_s_at	2	RAB39B	1.27	17.91	416	0.000191	0.009904
213134_x_at	2	BTG3	1.25	17.91	415	0.000191	0.009928
215438_x_at	3	GSPT1	-1.17	39.34	12	5.67E-07	0
218491_s_at	3	THYN1	-1.22	37.14	15	9.37E-07	0
231843_at	3	DDX55	-1.25	33.31	25	2.35E-06	0.0004
208910_s_at	3	C1QBP	-1.26	30.18	41	5.21E-06	0.001707
203867_s_at	3	NLE1	-1.24	26.56	85	1.38E-05	0.002235
223917_s_at	3	SLC39A3	-1.34	25.78	98	1.72E-05	0.002347
208660_at	3	CS	-1.13	26.26	89	1.5E-05	0.00236
224714_at	3	MKI67IP	-1.14	25.85	97	1.68E-05	0.002371
241937_s_at	3	WDR4	-1.37	25.71	100	1.75E-05	0.0024
201614_s_at	3	RUVBL1	-1.22	26.12	91	1.56E-05	0.002527
224046_s_at	3	PDE7A	-1.36	25.39	104	1.92E-05	0.002692
220762_s_at	3	GNB1L	-1.16	24.67	114	2.36E-05	0.00386
213669_at	3	FCHO1	-1.35	24.58	117	2.42E-05	0.004103
218058_at	3	CXXC1	-1.42	23.65	137	3.18E-05	0.004599
206037_at	3	CCBL1	-1.15	23.72	136	3.11E-05	0.004632
209664_x_at	3	NFATC1	-1.5	23.5	140	3.32E-05	0.004714
212625_at	3	STX10	-1.25	23.21	147	3.62E-05	0.004898
221953_s_at	3	MMP24	-1.12	22.18	179	4.92E-05	0.005363
214299_at	3	TOP3A	-1.16	21.72	201	5.66E-05	0.005721
201457_x_at	3	BUB3	-1.16	21.16	217	6.73E-05	0.006175
213132_s_at	3	MCAT	-1.18	20.89	231	7.31E-05	0.006494
206074_s_at	3	HMGA1	-1.25	20.83	232	7.45E-05	0.006509
217854_s_at	3	POLR2E	-1.13	20.77	235	7.59E-05	0.006553
236080_at	3	BE276063	-1.15	19.98	276	9.74E-05	0.006848
205264_at	3	CD3EAP	-1.37	20.47	247	8.33E-05	0.006923
218141_at	3	UBE2O	-1.17	20.18	263	9.15E-05	0.006996
200083_at	3	USP22	-1.09	20.22	257	9.01E-05	0.007121
212048_s_at	3	YARS	-1.16	19.58	298	0.000111	0.007181
205920_at	3	SLC6A6	-1.55	19.58	297	0.000111	0.007205
213581_at	3	PDCD2	-1.18	19.24	313	0.000123	0.007923
206106_at	3	MAPK12	-1.37	19.15	321	0.000127	0.008006
227968_at	3	PDDC1	-1.17	19.08	326	0.00013	0.008037
213521_at	3	PTPN18	-1.38	18.99	332	0.000134	0.008253
218590_at	3	PEO1	-1.14	18.83	338	0.000141	0.008669
201710_at	3	MYBL2	-1.25	18.72	348	0.000146	0.008736
216251_s_at	3	TTLL12	-1.28	18.62	360	0.000151	0.008917
221746_at	3	UBL4A	-1.14	18.52	369	0.000156	0.009079
207339_s_at	3	LTB	-2.15	18.51	370	0.000157	0.009135
202078_at	3	COPS3	-1.1	18.41	375	0.000162	0.00928
206055_s_at	3	SNRPA1	-1.17	18.41	374	0.000161	0.009305
209418_s_at	3	THOC5	-1.09	18.13	396	0.000177	0.009672
201075_s_at	3	SMARCC1	-1.24	18.15	392	0.000176	0.009719
218161_s_at	3	CLN6	-1.26	18.05	400	0.000182	0.0098
238190_at	3	TUFM	-1.11	18.03	405	0.000183	0.009802

203194_s_at	3	NUP98	-1.17	17.92	413	0.00019	0.009927
213918_s_at	4	NIPBL	-1.33	51.26	1	4.76E-08	0
219911_s_at	4	SLCO4A1	-1.61	50.95	2	5.06E-08	0
204394_at	4	SLC43A1	-1.7	49.9	3	6.2E-08	0
203060_s_at	4	PAPSS2	-3.38	45.73	5	1.43E-07	0
229332_at	4	GLOXD1	-2.36	39.24	13	5.79E-07	0
226611_s_at	4	PRR6	-1.83	35.84	18	1.27E-06	0.000556
209861_s_at	4	METAP2	-1.19	33.14	26	2.45E-06	0.000769
225777_at	4	C9orf140	-1.6	30.09	42	5.33E-06	0.001667
218883_s_at	4	MLF1IP	-1.33	29.23	47	6.68E-06	0.001702
218104_at	4	TEX10	-1.19	28.99	52	7.12E-06	0.001731
228977_at	4	LOC729680	-1.57	27.59	71	1.04E-05	0.001831
218993_at	4	RNMTL1	-1.14	30.36	38	4.97E-06	0.001842
201770_at	4	SNRPA	-1.17	28.22	61	8.76E-06	0.001967
218512_at	4	WDR12	-1.19	28.01	66	9.27E-06	0.00197
218081_at	4	C20orf27	-1.31	25.65	102	1.78E-05	0.002353
202559_x_at	4	C1orf77	-1.09	25.88	96	1.67E-05	0.002396
1564907_s_at	4	MATR3	-1.38	26.15	90	1.55E-05	0.002556
201196_s_at	4	AMD1	-1.21	25.21	105	2.02E-05	0.003143
201328_at	4	ETS2	-1.28	24.63	115	2.39E-05	0.003826
205733_at	4	BLM	-1.27	24.14	125	2.75E-05	0.0044
209036_s_at	4	MDH2	-1.08	23.98	126	2.88E-05	0.004603
226249_at	4	SNX30	-1.47	23.2	148	3.62E-05	0.005
221920_s_at	4	SLC25A37	-1.65	23.08	153	3.75E-05	0.005098
224460_s_at	4	L2HGDH	-1.3	23	155	3.84E-05	0.005226
201930_at	4	MCM6	-1.27	22.42	171	4.57E-05	0.00538
219110_at	4	NOLA1	-1.19	22.39	174	4.62E-05	0.005402
228252_at	4	PIF1	-1.26	22.39	173	4.62E-05	0.005434
202468_s_at	4	CTNNAL1	-1.37	22.44	169	4.56E-05	0.005444
224610_at	4	SNHG1	-1.19	22.53	166	4.43E-05	0.005482
202144_s_at	4	ADSL	-1.12	21.82	195	5.5E-05	0.005692
210044_s_at	4	LYL1	-1.56	21.23	214	6.58E-05	0.006168
1558381_a_at	4	GAPDHS	-1.17	21.25	212	6.54E-05	0.006226
224326_s_at	4	PCGF6	-1.1	21.28	210	6.48E-05	0.006286
201391_at	4	TRAP1	-1.26	20.65	240	7.87E-05	0.006833
238012_at	4	DPP7	-1.3	20.06	271	9.48E-05	0.0069
208273_at	4	ZNF695	-1.79	19.84	282	0.000102	0.006986
243745_at	4	AP1S2	-1.31	20.1	266	9.37E-05	0.006992
211623_s_at	4	FBL	-1.13	19.85	280	0.000101	0.007
204033_at	4	TRIP13	-1.27	19.55	301	0.000112	0.007176
204510_at	4	CDC7	-1.28	19.3	311	0.000121	0.007717
222781_s_at	4	C9orf40	-1.28	19.23	315	0.000124	0.007905
209421_at	4	MSH2	-1.28	19.15	319	0.000127	0.008056
228205_at	4	TKT	-1.14	19.08	325	0.00013	0.008062
204128_s_at	4	RFC3	-1.26	18.71	350	0.000147	0.008743
212036_s_at	4	PNN	-1.12	18.7	352	0.000147	0.00875
226262_at	4	AA534526	-1.22	18.6	363	0.000152	0.008898
244422_at	4	AI494573	-1.42	18.28	379	0.000168	0.009578
202534_x_at	4	DHFR	-1.3	18.22	387	0.000172	0.009638
225458_at	4	LOC25845	-1.2	18.06	399	0.000181	0.009749
202911_at	4	MSH6	-1.19	18.04	403	0.000183	0.009826

209900_s_at	4	SLC16A1	-1.23	18.04	401	0.000183	0.009875
1559220_at	4	BG025779	-1.14	17.9	417	0.000191	0.00988
204866_at	5	PHF16	3.85	37.31	14	9.02E-07	0
212240_s_at	5	PIK3R1	1.36	33.42	24	2.29E-06	0.000417
225347_at	5	ARL8A	1.28	35.01	19	1.55E-06	0.000526
225564_at	5	SPATA13	1.7	31.9	31	3.34E-06	0.001613
221808_at	5	RAB9	1.56	29.08	49	6.95E-06	0.001837
229817_at	5	ZNF608	2.19	28.11	65	9.03E-06	0.001846
221752_at	5	SSH1	1.48	29.64	43	6.01E-06	0.00186
220768_s_at	5	CSNK1G3	1.25	28.13	64	8.99E-06	0.001875
219274_at	5	TSPAN12	2.97	27.07	79	1.2E-05	0.001899
201847_at	5	LIPA	1.35	27.79	67	9.84E-06	0.00194
229828_at	5	AL044007	1.25	28.74	55	7.62E-06	0.002
210788_s_at	5	DHRS7	1.35	27.26	75	1.14E-05	0.002
218017_s_at	5	HGSNAT	1.48	28.49	57	8.14E-06	0.002105
242714_at	5	AW500340	3.08	25.73	99	1.74E-05	0.002323
202975_s_at	5	RHOBTB3	2.53	26.26	88	1.5E-05	0.002386
218909_at	5	RPS6KC1	1.24	25.92	95	1.65E-05	0.002421
216020_at	5	IFIH1	1.49	25.99	93	1.62E-05	0.002473
233759_s_at	5	SMEK2	1.13	26.09	92	1.57E-05	0.0025
209917_s_at	5	TP53AP1	1.37	24.99	109	2.15E-05	0.003303
212733_at	5	KIAA0226	1.24	24.84	110	2.25E-05	0.003818
210224_at	5	MR1	1.28	24.68	113	2.35E-05	0.003894
213194_at	5	ROBO1	4.61	24.59	116	2.41E-05	0.004138
203758_at	5	CTSO	1.42	24.42	119	2.53E-05	0.004286
212441_at	5	KIAA0232	1.23	23.76	135	3.07E-05	0.004667
202085_at	5	TJP2	1.34	23.91	128	2.94E-05	0.004688
203384_s_at	5	GOLGA1	1.24	23.09	152	3.74E-05	0.005066
203885_at	5	RAB21	1.22	22.42	172	4.58E-05	0.005349
202149_at	5	NEDD9	1.69	22.14	181	4.98E-05	0.005359
203227_s_at	5	TSPAN31	1.34	22.27	177	4.79E-05	0.005367
223134_at	5	BBX	1.29	22.68	162	4.24E-05	0.00537
222514_at	5	RRAGC	1.23	22.38	175	4.63E-05	0.005371
207571_x_at	5	C1orf38	1.68	21.94	191	5.29E-05	0.005393
225074_at	5	RAB2B	1.27	21.97	189	5.25E-05	0.005397
226196_s_at	5	C14orf179	1.31	22.43	170	4.57E-05	0.005412
212543_at	5	AIM1	1.77	22.81	157	4.07E-05	0.005414
200701_at	5	NPC2	1.26	22.47	167	4.51E-05	0.005449
1555832_s_at	5	KLF6	1.46	21.9	192	5.35E-05	0.005573
231866_at	5	LNPEP	1.48	21.82	194	5.49E-05	0.005722
217043_s_at	5	SYT7	1.32	21.86	193	5.43E-05	0.005751
219648_at	5	MREG	1.35	21.2	215	6.65E-05	0.006186
217743_s_at	5	TMEM30A	1.18	21.17	216	6.7E-05	0.006204
201393_s_at	5	IGF2R	1.29	21.27	211	6.51E-05	0.006256
209090_s_at	5	SH3GLB1	1.28	21.11	218	6.84E-05	0.006468
205403_at	5	IL1R2	3.17	20.82	233	7.48E-05	0.006481
204286_s_at	5	PMAIP1	1.4	20.98	224	7.1E-05	0.006563
224722_at	5	MIB1	1.39	20.91	228	7.26E-05	0.006579
202121_s_at	5	CHMP2A	1.15	20.78	234	7.57E-05	0.006581
224502_s_at	5	KIAA1191	1.26	20.71	236	7.75E-05	0.006737
225957_at	5	LOC153222	1.41	20.7	237	7.76E-05	0.006793

224957_at	5	LOC497661	1.18	20.44	251	8.43E-05	0.006853
220507_s_at	5	UPB1	1.39	20.52	245	8.21E-05	0.006857
200673_at	5	LAPTM4A	1.2	20.05	272	9.52E-05	0.006875
229146_at	5	C7orf31	1.24	19.94	277	9.85E-05	0.006895
223422_s_at	5	ARHGAP24	2.89	20	274	9.66E-05	0.006898
204198_s_at	5	RUNX3	1.25	19.83	284	0.000102	0.006937
222408_s_at	5	YPEL5	1.53	20.19	262	9.12E-05	0.006985
218020_s_at	5	ZFAND3	1.25	19.79	286	0.000103	0.006993
222498_at	5	AZI2	1.21	20.19	261	9.1E-05	0.007011
201133_s_at	5	PJA2	1.13	19.79	285	0.000103	0.007018
204780_s_at	5	FAS	1.42	20.11	265	9.34E-05	0.007019
218132_s_at	5	TSEN34	1.15	20.21	258	9.06E-05	0.007093
231927_at	5	ATF6	1.26	19.7	291	0.000106	0.007113
202318_s_at	5	SENP6	1.14	19.67	292	0.000107	0.007123
225522_at	5	AAK1	1.31	19.56	300	0.000111	0.007167
219863_at	5	HERC5	1.43	19.34	309	0.00012	0.00767
205641_s_at	5	TRADD	1.3	19.07	327	0.00013	0.008043
1554806_a_at	5	FBXO8	1.22	19.1	324	0.000129	0.008086
212498_at	5	AF056433	1.21	18.83	337	0.000141	0.008694
217974_at	5	TM7SF3	1.43	18.67	356	0.000148	0.008708
208783_s_at	5	CD46	1.18	18.74	345	0.000145	0.008725
201851_at	5	SH3GL1	1.15	18.73	347	0.000146	0.008732
213073_at	5	ZFYVE26	1.3	18.75	344	0.000145	0.00875
201193_at	5	IDH1	1.23	18.75	343	0.000145	0.008776
228478_at	5	AA889954	1.27	18.59	364	0.000152	0.008874
201311_s_at	5	SH3BGRL	1.15	18.62	361	0.000151	0.008892
226122_at	5	PLEKHG1	1.71	18.61	362	0.000151	0.008923
207098_s_at	5	MFN1	1.37	18.57	365	0.000154	0.008932
201999_s_at	5	DYNLT1	1.35	18.55	367	0.000154	0.008992
221918_at	5	PCTK2	1.35	18.42	373	0.000161	0.009276
222401_s_at	5	TMEM50A	1.14	18.31	377	0.000167	0.009549
203955_at	5	KIAA0649	1.38	18.26	382	0.00017	0.009581
226440_at	5	DUSP22	1.29	18.29	378	0.000168	0.009603
215930_s_at	5	CTAGE5	1.32	18.25	383	0.00017	0.009634
209004_s_at	5	FBXL5	1.21	18.2	389	0.000173	0.009666
231690_at	5	AI962352	1.45	18.07	398	0.000181	0.009774
227056_at	5	KIAA0141	1.26	18	408	0.000185	0.009804
203732_at	5	TRIP4	1.16	18.04	402	0.000183	0.009851
201968_s_at	5	PGM1	1.2	17.95	411	0.000188	0.009854
212150_at	5	KIAA0143	1.19	17.91	414	0.00019	0.009952
200977_s_at	5	TAX1BP1	1.27	17.87	420	0.000193	0.009976
