# Supplementary material for: Transcriptional Dysregulation in NIPBL and Cohesin Mutant Human Cells
Source: PLoS Biol. 2009 May 26;7(5):e1000119. doi: 10.1371/journal.pbio.1000119 (PMC2680332; doi:10.1371/journal.pbio.1000119)
Supplement: Table S6 — 32 genes chosen by clustering-based feature selection for custom array analysis. (0.23 MB PDF) [file pbio.1000119.s010.pdf]

Table S6. 32 genes chosen by clustering based feature selection for custom array analysis.

Gene symbol	Affy_ID	Cluster	F_Score	Rank	p_Value	FDR	Fold change
ADCY1	213245_AT	1	19.51	302	0.000113	0.007285	-2.76
FGD6	219901_AT	1	20.1	267	9.37E-05	0.006966	-2.16
ID3	207826_S_AT	1	19.19	317	0.000125	0.008013	-1.77
JDP2	226267_AT	1	19	331	0.000134	0.008278	-1.52
LOC440829	244467_AT	1	39.86	11	5.04E-07	0	-2.65
NFATC2 (AI770171)	228442_AT	1	24.19	124	2.71E-05	0.004435	-2.06
NMB	205204_AT	1	41.14	7	3.8E-07	0	-1.17
PCDHG	209079_X_AT	1	31.08	35	4.12E-06	0.002	-2.06
TRERF1	238520_AT	1	18.88	336	0.000139	0.008423	-2.35
ATP10D	213238_AT	2	23.44	142	3.38E-05	0.004718	1.71
KIAA1450	225924_AT	2	27.48	73	1.07E-05	0.001918	2.62
KIFAP3	203333_AT	2	45.71	6	1.44E-07	0	1.41
PTHB1	37549_G_AT	2	40.55	8	4.33E-07	0	1.33
CXXC1	218058_AT	3	23.65	137	3.18E-05	0.004599	-1.42
LTB	207339_S_AT	3	18.51	370	0.000157	0.009135	-2.15
PTPN18	213521_AT	3	18.99	332	0.000134	0.008253	-1.38
SLC6A6	205920_AT	3	19.58	297	0.000111	0.007205	-1.55
GLOXD1	229332_AT	4	39.24	13	5.79E-07	0	-2.36
NIPBL	213918_S_AT	4	51.26	1	4.76E-08	0	-1.33
PAPSS2	203060_S_AT	4	45.73	5	1.43E-07	0	-3.38
PRR6	226611_S_AT	4	35.84	18	1.27E-06	0.000556	-1.83
SNX30	226249_AT	4	23.2	148	3.62E-05	0.005	-1.47
ZNF695	208273_AT	4	19.84	282	0.000102	0.006986	-1.79
AIM1	212543_AT	5	22.81	157	4.07E-05	0.005414	1.77
ARHGAP24	223422_S_AT	5	20	274	9.66E-05	0.006898	2.89
ARL8A	225347_AT	5	35.01	19	1.55E-06	0.000526	1.28
MAP3K5 (AW500340)	242714_AT	5	25.73	99	1.74E-05	0.002323	3.08
PHF16	204866_AT	5	37.31	14	9.02E-07	0	3.85
RHOBTB3	202975_S_AT	5	26.26	88	1.5E-05	0.002386	2.53
ROBO1	213194_AT	5	24.59	116	2.41E-05	0.004138	4.61
TSPAN12	219274_AT	5	27.07	79	1.2E-05	0.001899	2.97
ZNF608	229817_AT	5	28.11	65	9.03E-06	0.001846	2.19
