# Supplementary material for: Transcriptional Dysregulation in NIPBL and Cohesin Mutant Human Cells
Source: PLoS Biol. 2009 May 26;7(5):e1000119. doi: 10.1371/journal.pbio.1000119 (PMC2680332; doi:10.1371/journal.pbio.1000119)
Supplement: Table S7 — Cohort of 101 individuals of European descent selected for custom array validation. Clinical evaluation and gene mutations of this cohort are listed. (0.27 MB PDF) [file pbio.1000119.s011.pdf]

Table S7. Cohort of 101 individuals of European descent selected for custom array validation. Clinical evaluation and gene mutations of this cohort are listed.

	Phenotype	NIPBL mutation	#Samples	
[A]Original training samples	Healthy control	No	17	1 Female and 1 Male were split and labeled as different samples
	Severe CdLS	Yes	14	1 Female and 1 Male were labeled as different samples (technique duplicate)
[B]New samples	Healthy control	No	4	
	Severe CdLS	Yes	6	
	Moderate CdLS	Yes	9	
	Mild CdLS	Yes	26*	
	Moderate CdLS	No	4	
	Mild CdLS	No, but w/**SMC1A** mutations	9	
	Mild CdLS	No	8	
	[C]Other congenital multisystem disorders	No	4	1 Roberts syndrome proband, 2 Alagille probands, 1 unknown genetic disorder

[A] All of the 17 healthy individuals and the 14 severely affected probands with *NIPBL* mutations have been tested on Affymatrix expression arrays; also serving as training samples for custom array analysis.
[B] Testing samples.
[C] Roberts syndrome, although distinct, displays clinically overlap with CdLS, and its disease causing gene *ESCO2* functions in the same pathway as NIPBL; Alagille syndrome is due to mutations in the *JAG1* gene, a ligand in the Notch signaling pathway, and is clinical quite different from CdLS.
*15 probands were randomly selected as training samples for a more robust classifier.
