# Supplementary material for: Transcriptional Dysregulation in NIPBL and Cohesin Mutant Human Cells
Source: PLoS Biol. 2009 May 26;7(5):e1000119. doi: 10.1371/journal.pbio.1000119 (PMC2680332; doi:10.1371/journal.pbio.1000119)
Supplement: Table S8 — 56 probes designed for the 32 selected genes for the custom array. (0.22 MB PDF) [file pbio.1000119.s012.pdf]

Table S8. 56 probes designed for the 32 selected genes the custom array.

Probe Name	Gene	Probe sequence
204866_AT_2	PHF16	AAGGAGCCTTCAGTTGTAAATTTCAATTACCCCAAAATGTATTTGCTACA
204866_AT_3		TTCATGCCTTTCAATTCTGAGTGGGAGGAAAAGCAAACATCAAAA
205204_AT_2	NMB	GGTCACTTCATGGGCAAGAAGAGTCTGGAGCCTTC
205204_AT_3		GGGACCCTGTTGATGGCCCCATCTGGATGTAAATC
205920_AT_3	SLC6A6	ACTTCCAAGCTCAGTCTAAATGAAACCGAAACGTGACCAC
207339_S_AT_2	LTB	CGTCAACATCAGTCACCCCGATATGGTGGACTTCG
207339_S_AT_3		GGGAATATGAGTGCGTGGTGCGAGTGCGTGAATAT
207826_S_AT_2	ID3	CAAGTTCTAAGGTCTTTTCAGAGCGTGGAGGTGTGGAAGG
207826_S_AT_3		CAGAGCTGGTCTTCTGGTCTCCTTGGAGAAAGGTTCTGTT
208273_AT_2	ZNF695	AATTCATACTGGTGAGAAACCATACAAATGTGAGGAATGTGGCAAAG
208273_AT_3		TGACTAAGACCTACTATTCGATAGCACAACAGGGTGACTAC
209079_X_AT_3	PCDHGC	GCGACCCCCTCCCCCGTACTGACTTCTCCTATAAG
212543_AT_1	AIM1	ATCTTCTCTGGAGTCTATGGTAGGCAATTATGGTCACTGGAATAGTT
212543_AT_2		GGGTAAGACATTCTACAGTAGCCTGTGCTGAACTGATCTCTTAAA
213194_AT_1	ROBO1	GATAGATAAGTATCAGGTCTGACCCCAGTGGAAAACAAAGCCAAA
213194_AT_2		AACAATCCATGTGATTAATGTTTTCATTATGTTCATGTAAGAAGCCCCTT
213238_AT_1	ATP10D	TATATTTGAAAGTTGTCAGCCACCAGTCATCCAGAATTTCCTTCCT
213238_AT_2		CAGGGTTTAGTTTTCTACCTTCTGCCTACTATTTTGGTCTGACATTTT
213245_AT_1	ADCY1	CCCGCTCAAACAACAATGTCCTTATTATGATGACCATCTCGTAGT
213245_AT_3		TTTGGCTAGATAGGGTTGTGTCCCTCTATGGAATGGAGAGTGATG
213521_AT_1	PTPN18	GGTGCTGACCTCTGTGTTGCTGGATAATGAGTCATCTATCT
213521_AT_3		GGTGATGCTGAGGTGTGGATTTTTAACAGTTCCCAGACTTT
213918_S_AT_1	NIPBL	GCGGAGGAGGAGTCAACGTATTTCGCAGCGTATTAC
213918_S_AT_2		TTCAGGGGTGAGGCGGAGGAGGAGTCAACGTATTT
218058_AT_1	CXXC1	CCCGTGTGTCCGTTCCTCCACTCATCTGTTTCTCC
218058_AT_3		TGACCGCCCATCTGCCTTTATCAGAGGGACTGTCC
219274_AT_1	TSPAN12	GCTTATCTTTGCCTTCTCCAAACAAGAAGCAATAGTCTCCAAGTC
219274_AT_3		TAGTTGATTCAGAAAGGACTTGTATGCTGTTTTTCTCCCAAATGAAGACT
219901_AT_1	FGD6	CTGCCAAGATAAAGCCAACCAGACCCTTCATCAAAGAAATTGTTT
219901_AT_2		AAACACATAGCAAGGAGCAAATTTCCACAAAGTGCTTGGTTTAGG
219901_AT_3		ATTCGGCTCAGAAGTGGATAGAAGCATTTCAGGAAGGCACAATAT

223422_S_AT_1	ARHGAP24	ATGTGTACCAAAGTTATATCATGCCCCATAATGCTACTGTCAAGTGTTAC
223422_S_AT_3		GAACCGAGAGAGGAAACACAATATGGATTCAGTGAGCCTGCTTTC
225347_AT_3	ARL8A	TCTCATTTTGCAGAGTTGCACAAGGAGAGAACTCAGCATG
225924_AT_3	KIAA1450	ACACTCAGGATTCCAGTCAGAACCTAATCCTCATATCTATTGCCTAC
226249_AT_2	SNX30	AAGTGCTTGAGAGAGTGTGTTGATAAGAAAGTGATTTATTTACAGATGGA
226249_AT_3		TTTTTGTTGACTTTTTGATGCTGGCACGAGGTTTTTGTCCACTTT
226267_AT_3	JDP2	GCATTTCCATCCTTGTAAACCCTTCATAGTACTCAGTCCTGTATCG
226611_S_AT_2	PRR6	GCAGAATAGACACTTCATTGTTCCAGCTTCTCGCTTCAAG
228442_AT_1	NFATC2	AGGCTTAGGTTTAGAAAATCCTGACTGTTAAAGGCGTTTGAATACATCA
228442_AT_3	(AI770171)	GGCTGTTGGGACAAGCTTAGCATCCTGGACATCTT
229332_AT_1	GLOXD1	AGGTGTGACTTCCATTTCATCAGTGCCTGCCAGAA
229332_AT_2		ATACTACCAGCAGCCAGGAAAGGAGAGGCAGATCC
229817_AT_1	ZNF608	AAAGATTAAAATGGGGAAATAATGAGCATCTGTGAATAATGAGGTGTCA
229817_AT_3		TATCAGTGTGCCTGAACCTTGCATATCCTTCACATATTTCCCATAAG
238520_AT_1	TRERF1	TGTTCTGGCTTCGTTATGTTCTATTCTCAGCAGATGAATTGCATG
238520_AT_3		CTTAACTGTATTTTCCCGGAATGCAAGTCCCTTGGTCCATATTAAAG
242714_AT_3	MAP3K5 (AW500340)	ACCTGAATGAATGAATATAACCTCTTCACGGTTCAGTGTTTTCATAAGCA
244467_AT_2	LOC440829	GCGGGTTCGTCTTGTAAATAAGCCCTTTTATGTGGTTCCT
37549_G_AT_3	PTHB1	AAGAGGGGTTGGGACTTTTTACTTCACTAGGAGAACTTGTAACAC
202975_S_AT_1	RHOBTB3	CCTTTCAACCTGGCTACTTCATTTCATTGCTACTAACTACCTCATCTTC
202975_S_AT_3		TCCCTTAAAGATGTCCTGTTGCTTTAGTGATATTTAGACCCCTCTCA
203060_S_AT_2	PAPSS2	CGGATACTGAAAGGTCGAGTTTTCTGAACTGCACTGATTTTATTGC
203060_S_AT_3		TTTCAAGCTGTTCTGAGACATCTTCTGATGGCTTTACTTCCTGAG
203333_AT_2	KIFAP3	GCCCTGATGAACCTTACTACTATGGCTATGGATCTTGATAAAGTATCTGT
203333_AT_3		GATGGATGAGAGTGAGCAGTACTTGTATGGTGATGATCGAATTGAG
