# Supplementary material for: Transcriptional Dysregulation in NIPBL and Cohesin Mutant Human Cells
Source: PLoS Biol. 2009 May 26;7(5):e1000119. doi: 10.1371/journal.pbio.1000119 (PMC2680332; doi:10.1371/journal.pbio.1000119)
Supplement: Table S9 — Step wise method to select the 23- and ten-gene classifiers and the three-gene biomarkers. (0.23 MB PDF) [file pbio.1000119.s013.pdf]

Table S9. Step wise method to select the 23- and 10-gene classifiers and the 3- gene biomarkers.

A. Samples and statistical information.

Training groups	
N	17 healthy controls
P1	14 severe CdLS probands (with NIPBL mutation)
P2	15 mild CdLS probands (with NIPBL mutation)
P3A	8 mild CdLS probands (without NIPBL mutation)
P3B	9 mild CdLS probands (with SMC1A mutation)
P3	P3A+P3B
Mean	Group average
SD	Standard deviation
POOL_grc	Pooled standard deviation
p_groups	Student's t test between 2 groups (2 sided, unequal variance)

B. Statistical results

Gene	Mean _N	Mean _P1	Mean _P2	Mean _P3	SD_N	SD_P 1	SD_P 2	SD_P 3	POOL _N+P	POOL _N+P	POOL _N+P	p_N vs. P1	p_N vs. P2	p_N vs. P3
NFATC2	6.153	5.366	5.370	5.448	0.531	0.593	0.521	0.387	0.560	0.527	0.465	**0.001**	**0.000**	**0.000**
ZNF608	5.153	5.870	5.537	5.493	0.330	0.555	0.298	0.301	0.445	0.315	0.316	**0.000**	**0.002**	**0.004**
PAPSS2	4.811	4.166	4.200	4.405	0.452	0.198	0.126	0.301	0.361	0.341	0.384	**0.000**	**0.000**	**0.005**
ZNF695	4.819	4.524	4.635	4.596	0.277	0.285	0.296	0.201	0.281	0.286	0.242	**0.007**	0.081	0.012
ADCY1	3.749	3.579	3.603	3.585	0.226	0.053	0.065	0.110	0.172	0.171	0.178	**0.008**	0.020	0.013
GLOXD1	5.983	5.569	5.666	5.777	0.280	0.303	0.280	0.181	0.290	0.280	0.235	**0.001**	**0.003**	0.017
LOC440829	6.976	5.840	6.247	6.518	0.619	0.532	0.463	0.557	0.582	0.552	0.589	**0.000**	**0.001**	0.030
ROBO1	5.651	7.138	6.407	6.489	1.189	1.027	0.978	1.090	1.120	1.096	1.141	**0.001**	0.058	0.040
PCDHG	9.684	9.838	9.647	9.470	0.335	0.307	0.368	0.306	0.323	0.351	0.321	0.195	0.767	0.061
ATP10D	6.360	6.931	6.791	6.580	0.351	0.166	0.316	0.336	0.283	0.335	0.343	**0.000**	**0.001**	0.071
MAP3K5	6.237	7.584	7.131	6.769	0.764	1.152	0.539	0.920	0.957	0.669	0.846	**0.001**	**0.001**	0.076
PHF16	4.867	6.213	5.712	5.336	0.687	0.949	0.589	0.893	0.815	0.643	0.797	**0.000**	**0.001**	0.097
PRR6	6.248	5.702	5.911	6.116	0.233	0.395	0.446	0.254	0.316	0.349	0.244	**0.000**	0.016	0.123
ARHGAP24	4.602	5.268	4.747	4.917	0.371	0.748	0.537	0.756	0.571	0.456	0.595	**0.007**	0.389	0.137
FGD6	4.707	4.401	4.593	4.587	0.254	0.212	0.233	0.237	0.236	0.244	0.245	**0.001**	0.196	0.162
PTPN18	6.731	6.566	6.603	6.610	0.273	0.202	0.181	0.270	0.244	0.235	0.272	0.063	0.124	0.204
LTB	6.717	5.794	6.029	6.482	0.733	0.576	0.619	0.390	0.667	0.682	0.587	**0.000**	**0.007**	0.254
TRERF1	5.068	4.613	4.890	4.937	0.421	0.342	0.450	0.267	0.387	0.435	0.352	**0.002**	0.259	0.287
NIPBL	6.524	6.108	6.304	6.376	0.452	0.500	0.456	0.407	0.474	0.454	0.430	0.023	0.182	0.323
AIM1	8.608	9.417	8.844	8.453	0.493	0.484	0.374	0.425	0.489	0.442	0.460	**0.000**	0.136	0.335
ID3	9.059	8.332	9.039	9.213	0.527	0.438	0.324	0.404	0.489	0.444	0.469	**0.000**	0.898	0.347
PTHB1	4.914	5.166	5.069	4.968	0.201	0.203	0.200	0.209	0.202	0.201	0.205	**0.002**	0.037	0.446
SNX30	6.095	5.880	5.871	6.022	0.263	0.242	0.237	0.317	0.253	0.251	0.291	0.025	0.017	0.474
CXXC1	7.891	7.724	7.900	7.955	0.268	0.143	0.174	0.305	0.221	0.229	0.287	0.037	0.912	0.516
KIFAP3	6.131	6.614	6.341	6.177	0.197	0.176	0.245	0.214	0.188	0.221	0.206	**0.000**	0.013	0.518
NMB	4.163	4.041	4.060	4.215	0.218	0.078	0.061	0.298	0.170	0.165	0.261	0.044	0.079	0.564
KIAA1450	5.119	5.837	5.648	5.247	0.611	0.447	0.665	0.756	0.544	0.637	0.687	**0.001**	0.027	0.593
TSPAN12	6.463	7.155	6.744	6.527	0.407	0.548	0.386	0.345	0.475	0.397	0.377	**0.001**	0.055	0.624
RHOBTB3	6.486	6.954	6.618	6.563	0.459	0.518	0.508	0.588	0.486	0.482	0.527	0.014	0.446	0.670
SLC6A6	3.898	3.851	3.873	3.879	0.068	0.069	0.068	0.169	0.068	0.068	0.129	0.068	0.307	0.685
ARL8A	5.719	6.009	5.985	5.694	0.179	0.159	0.203	0.237	0.170	0.191	0.210	**0.000**	**0.001**	0.739
JDP2	7.501	7.212	7.493	7.502	0.363	0.364	0.244	0.235	0.364	0.313	0.306	0.036	0.944	0.995
