# Supplementary material for: Transcriptional Dysregulation in NIPBL and Cohesin Mutant Human Cells
Source: PLoS Biol. 2009 May 26;7(5):e1000119. doi: 10.1371/journal.pbio.1000119 (PMC2680332; doi:10.1371/journal.pbio.1000119)
Supplement: Table S10 — Intragenic cohesin binding in mapped human RefSeq genes. The total number of mapped human RefSeq transcripts is 15,162, whereas 4,784 genes are not transcribed in LCLs (group A); 9,199 genes are transcribed but not differentially expressed in CdLS (group B); and 1,179 genes are both transcribed and differentially expressed in CdLS (group C). (A) Cohesin binding is reduced in group A genes (18.9% of 4,784 genes) but increased in group C genes (27.0% of 1,179 genes) as compared to all the mapped transcripts (22.0% of 15,162 genes). Group B genes demonstrate little change (22.9% of 9,199 genes) as compared to all mapped transcripts. In CdLS, the number of genes bound by cohesin in all the groups is significantly reduced (all transcripts, 22.0% → 16.0%; group A genes, 18.9% → 13.8%; group B genes, 22.9% → 16.8%; group C genes, 27.0% → 18.5%). (B) In both control and CdLS, when compared to the number of genes bound by cohesin in all the mapped transcripts (22.0% in control and 16.0% in CdLS), group A has a significantly reduced percentage of genes bound by cohesin (p≤7.2e−6 in control and p≤0.000187 in CdLS), whereas group C has a significantly increased percentage of genes bound by cohesin (p≤7.44e−5 in control and p≤0.0249 in CdLS), and group B does not demonstrate a statistically significant change in cohesin binding (p≤0.0864 in control and p≤0.0836 in CdLS). *p, binomial proportions comparing intragenic cohesin binding between control and CdLS; **p, binomial proportions comparing intragenic cohesin binding between individual group (A, B, or C) and all the mapped transcripts. (0.27 MB PDF) [file pbio.1000119.s014.pdf]

Table S10. Intragenic cohesin binding in mapped human RefSeq genes. The total number of mapped human RefSeq transcripts is 15,162, whereas 4,784 genes are not transcribed in LCLs (group A); 9,199 genes are transcribed but not differentially expressed in CdLS (group B); and 1,179 genes are both transcribed and differentially expressed in CdLS (group C). (A) Cohesin binding is reduced in group A genes (18.9% of 4,784 genes) but increased in group C genes (27.0% of 1,179 genes) as compared to all the mapped transcripts (22.0% of 15,162 genes). Group B genes demonstrate little change (22.9% of 9,199 genes) as compared to all mapped transcripts. In CdLS, the number of genes bound by cohesin in all the groups is significantly reduced (all transcripts: 22.0% ⇒ 16.0%; Group A genes: 18.9% ⇒ 13.8%; Group B genes: 22.9% ⇒ 16.8%; group C genes: 27.0% ⇒ 18.5%). (B) In both control and CdLS, when compared to the number of genes bound by cohesin in all the mapped transcripts (22.0% in control and 16.0% in CdLS), group A has a significantly reduced percentage of genes bound by cohesin (*p*<= 7.2e-06 in control and *p*<= 0.000187 in CdLS), whereas group C has a significantly increased percentage of genes bound by cohesin (*p*<= 7.44e-05 in control and *p*<= 0.0249 in CdLS) and group B does not demonstrate a statistically significant change in cohesin binding (*p* <= 0.0864 in control and *p* <= 0.0836 in CdLS). ***p***: binomial proportions comparing intragenic cohesin binding between control and CdLS; ****p***: binomial proportions comparing intragenic cohesin binding between individual group (A, B, or C) and all the mapped transcripts.

A

	**All mapped transcripts**	**Group A genes (non-transcribed)**	**Group B genes (transcribed, neutral)**	**Group C gene (transcribed , disease specific)**
**Control**	22.0% (3,333/15,162)	18.9% (906/4,784)	22.9% (2,109/9,199)	27.0% (318/1,179)
**CdLS**	16.0% (2,425/15,162)	13.8% (658/4,784)	16.8% (1,549/9,199)	18.5% (218/1,179)
p	<= 2.58e-40	<= 7.11e-12	<= 4.54e-25	<= 8.95e-07

B

	**All mapped transcripts**	**Group A genes (non-transcribed)**	**Group B genes (transcribed, neutral)**	**Group C genes (transcribed , disease specific)**
**Control**	22.0%	18.9% (****p***<= 7.2e-06)	22.9% (****p*** <= 0.0864)	27.0% (****p*** <= 7.44e-05)
**CdLS**	16.0%	13.8% (****p*** <= 0.000187)	16.8% (****p*** <= 0.0836)	18.5% (****p*** <= 0.0249)
