# Supplementary material for: Transcriptional Dysregulation in NIPBL and Cohesin Mutant Human Cells
Source: PLoS Biol. 2009 May 26;7(5):e1000119. doi: 10.1371/journal.pbio.1000119 (PMC2680332; doi:10.1371/journal.pbio.1000119)
Supplement: Table S11 — Specific primer pairs used for ChIP-qPCR validation. Primer pair 1 and 2 amplify regions that are bound by cohesin equally in healthy and CdLS cells, and served here as positive controls. Primer pair 3 and 4 amplify regions that are not bound by cohesin in either healthy or CdLS cells, and served as negative controls. Primer pair 5 to 13 amplify regions that were identified as having lost cohesin binding in CdLS cells by the qualitative ChIP array studies. (0.22 MB PDF) [file pbio.1000119.s015.pdf]

Table S11. Specific primer pairs used for ChIP-qPCR validation. Primer pair #1 and #2 amplify regions that are bound by cohesin equally in healthy and CdLS cells, and served here as positive controls. Primer pair #3 and #4 amplify regions that are not bound by cohesin in either healthy or CdLS cells, and served as negative controls. Primer pair # 5 to # 13 amplify regions that were identified as having lost cohesin binding in CdLS cells by the qualitative ChIP array studies.

Primer ID	left primer	right primer	Chr	position
1	ACCACAAGGTGGCAGAATAAG	CACATGCATATAGGCTAACGAAG	chr3	79653256-79653385
2	AACACAAGCGGTCTCTGTCC	GATGGCTCTTCCATGTACGG	chr18	72239945-72240119
3	GAGCTCTAAGGGAGGCTCCG	CATCATGGTGTCCTCACAGG	chr11	1983833-1983994
4	TGCCATGCGTTGAAAATATCC	TGCTTTCTGAAGTTGCCAAGC	chr7	27213317-27213491
5	GGCACGATTACTTAATGTTCACG	CTGCAGCAGTGGTCGC	chr5	124071139-124071295
6	ACTTGGTTCTGGAAGGCTCTC	TTGAGGAGACACCAGAGTGAC	chr5	124088545-124088657
7	ACGAGAGTGTGGCTCATGC	AGGAAGGACGACTCATCTGG	chr4	47187478-47187660
8	AGTCCTTCTCCGCACCATC	CCATTATCTTCACACACCATATCC	chr13	112645175-112645323
9	AGGAGATGCTGTGCTGATCC	TATCCAGAGCGAGTGTGCTG	chr1	153443443-153443596
10	CCAGGCACTGTCCTTCAAC	TGTGTTGCATGTAATGTACTAGCTC	chr6	10586629-10586819
11	GCCAAGCTAATGCACATGG	CACAGCTCAAGAGCACCATC	chr6	114422682-114422857
12	TTCCTCTTCAGTGCTCTTCAAC	TTGCAAGCGTGTGGACTC	chr16	71623997-71624129
13	AGTGAGAGAGAGGTTCACAGTCC	TGACACCATTGAGATGTTGACTC	chr14	96721538-96721691

*Primer #28 (negative for CTCF nd RAD21) Wendt KS. *et al.* Nature 2008 451 796

*Primer #36 (negative for CTCF nd RAD21) Wendt KS. *et al.* Nature 2008 451 796
