# Supplementary material for: Transcriptional Dysregulation in NIPBL and Cohesin Mutant Human Cells
Source: PLoS Biol. 2009 May 26;7(5):e1000119. doi: 10.1371/journal.pbio.1000119 (PMC2680332; doi:10.1371/journal.pbio.1000119)
Supplement: Table S12 — Intragenic cohesin binding in the classifier genes and the gene ontology analysis. The appearance of a cohesin binding site is described as “+,” binding in both CdLS proband and control cells is shown. The involvements of multiple bio-functions and canonical pathways of each gene are also listed based on the IPA analysis. The 10-gene classifier and the three biomarkers are part of the 23-gene classifier. (0.27 MB PDF) [file pbio.1000119.s016.pdf]

Table S12. Intragenic cohesin binding in the classifier genes and the gene ontology analysis. The appearance of a cohesin binding site is described as "**+** ", binding in both CdLS proband and control cells is showed. The involvements of multiple bio-functions and canonical pathways of each gene are also listed based on the IPA analysis. The 10-gene classifier and the 3 biomarkers are part of the 23-gene classifier.

Genes	Intragenic binding in CdLS	Intragenic binding in controls	Bio-functions[⫯]	Canonical pathways[⫯⫯]
**PAPSS2**[ᛏŦ]	+	+	+	+
**ZNF608**[ᛏŦ]	+	+ *		
**NFATC2** [ᛏŦ]			+	+
**MAP3K5**[ᛏ]	+	+	+	+
**PHF16**[ᛏ]	+	+	+	
**LTB**[ᛏ]		+	+	
**ATP10D**[ᛏ]		+		
**ARL8A**[ᛏ]				
**HPDL(GLOXD1)**[ᛏ]				
**LOC440829**[ᛏ]				
**KIFAP3**	+	+	+	
**ARHGAP24**	+	+	+	
**TSPAN12**	+	+	+	
**AIM1**	+	+	+	
**TRERF1**		+	+	
**BBS9 (PTHB1)**		+	+	
**ID3**			+	
**FGD6**	+	+		
**KIAA1450**	+	+		
**ROBO1**	+	+		
**ADCY1**				+
**ZNF695**				
**PRR6**				

Ŧ 3 biomarkers
ᛏ 10-gene classifier
⫯ Identified by IPA, including 47 biological functions or disease conditions
⫯⫯ Identified by IPA, including 32 canonical pathways
* but have lost 3 sites including one at 5'-UTR
