# Supplementary material for: Transcriptional Dysregulation in NIPBL and Cohesin Mutant Human Cells
Source: PLoS Biol. 2009 May 26;7(5):e1000119. doi: 10.1371/journal.pbio.1000119 (PMC2680332; doi:10.1371/journal.pbio.1000119)
Supplement: Table S13 — Dysregulated genes (FDR<0.05) identified in CdLS probands with NIPBL mutations that are functionally related to cohesion pathways. Genes that have FDR between 0.05 and 0.1 are highlighted in red. (0.23 MB PDF) [file pbio.1000119.s017.pdf]

Table S13. Dys-regulated genes (FDR<0.05) identified in CdLS probands with *NIPBL* mutations that are functionally related to cohesion pathways. Genes that have FDR between 0.05 and 0.1 are highlighted in red.

Gene Symbol	Fold Change	FDR	Roles in sister chromatid cohesion	Reference
NIPBL	-1.34	0.000	Cohesin loading	Deardorff *et al.*, 2007
Mau2 (KIAA0892)	1.10	0.026	Cohesin loading	Seitan *et al.*, 2006
STAG1	1.16	0.023	Stromalin component, homologous to SCC3	Anazawa *et al.*, 2004
PSD5B (ARPIN)	-1.16	0.032	Direct associated with and stabilize cohesion	Losada *et al.*, 2005
Sororin (CDCA5)	-1.17	0.028	G2 phase cohesin	Schmitz et al., 2007
TIPIN	-1.17	0.018	Initiation and maintenance of chromosome cohesion	Gotter *et al.*, 2007
SMC4	-1.17	0.061	Condesin	Lam *et al.* 2006
NCAPD3	-1.13	0.047	Codensin	Losada *et al.*, 2002
NCAPH	-1.18	0.040	Condensin	Losada *et al.*, 2002
NEK1	1.17	0.039	Mitotic kinase, cohesin removal	Li and Li, 2006
PLK1	-1.27	0.029	Mitotic kinase, cohesin removal	Li and Li, 2006
AURKB	-1.22	0.025	Mitotic kinase, cohesin removal, condensin loading	Lipp et al., 2007; Li and Li, 2006
RECQL4	-1.27	0.077	DNA helicase, chromosome cohesion	Mann *et al.*, 2005
DDX11	-1.23	0.029	DNA helicase, binding to cohesin complex	Parish *et al.*, 2006
ORC1L	-1.23	0.047	Replication initiation	Bell and Dutta, 2002
ORC2L	-1.17	0.011	Replication initiation	Bell and Dutta, 2002
MCM2	-1.18	0.029	Replication initiation	Bell and Dutta, 2002
MCM3	-1.21	0.017	Replication initiation	Bell and Dutta, 2002
MCM4	-1.38	0.031	Replication initiation	Bell and Dutta, 2002
MCM5	-1.26	0.025	Replication initiation	Bell and Dutta, 2002
MCM6	-1.28	0.005	Replication initiation	Bell and Dutta, 2002
MCM10	-1.20	0.049	Replication initiation	Bell and Dutta, 2002
RFC2	-1.23	0.027	Replication initiation and chromosome cohesion	Skibbens, 2005
RFC3	-1.26	0.009	Replication initiation and chromosome cohesion	Skibbens, 2005
RFC4	-1.15	0.039	Replication initiation and chromosome cohesion	Skibbens, 2005
RFC5	-1.16	0.087	Replication initiation and chromosome cohesion	Skibbens, 2005
BUB1B	-1.19	0.020	Kinetochore proteins	Taylor *et al.*, 2004
BUB3	-1.17	0.006	Kinetochore proteins	Taylor *et al.*, 2004
CENPA	-1.16	0.056	Kinetochore proteins	Yuen et al., 2005
CENPL	-1.22	0.024	Kinetochore proteins	Yuen et al., 2005
SMARCA4	-1.39	0.017	Chromatin remodeling SWI/SNF family members	Shanahan *et al.*, 1999
SMARCC1	-1.24	0.010	Chromatin remodeling SWI/SNF family members	Shanahan et al., 1999
ATRX	+1.18	0.070	chromatin remodeling enzyme, chromosome cohesion	Ritchie et al., 2008
KIFAP3	1.41	0.000	Direct binding to SMC3 and KIF3A/3B	Shimizu *et al.*, 1998
PTTG1IP	1.23	0.047	Direct binding to PTTG1, the human homolog of securin	Vlotides *et al.*, 2007
**DNA-damage-checkpoint proteins binding to SMC1 and SMC3**				
BRCA1	-1.20	0.017	double strand break repair	Kim et al., 2002; Yazdi *et al.*, 2002
BLM	-1.27	0.004	double strand break repair	Yazdi et al., 2002
NBS1	1.25	0.017	double strand break repair	Kim et al., 2002; Yazdi *et al.*, 2002
