# Supplementary material for: Transcriptional Dysregulation in NIPBL and Cohesin Mutant Human Cells
Source: PLoS Biol. 2009 May 26;7(5):e1000119. doi: 10.1371/journal.pbio.1000119 (PMC2680332; doi:10.1371/journal.pbio.1000119)
Supplement: Text S1 — Supporting methods and statistical analysis. (1.31 MB PDF) [file pbio.1000119.s018.pdf]

Supporting Information

Transcriptional Dysregulation in *NIPBL* and Cohesin Mutant Human Cells

Jinglan Liu[1], Zhe Zhang[2], Masashige Bando[3], Takehiko Itoh[3], Matthew A. Deardorff[1, 4], Dinah Clark[1], Maninder Kaur[1], Stephany Tandy[1], Tatsuro Kondoh[5], Eric Rappaport[6], Nancy B. Spinner[1, 4], Hugo Vega[7], Laird G. Jackson[8], Katsuhiko Shirahige[3], Ian D. Krantz[1, 4]*

*To whom correspondence should be addressed.
E-mail: ian2@mail.med.upenn.edu

Contents:

Supporting Information Figure Legends

Figure S1. Expression level of three genes (*NFATC2*, *PAPSS2* and *ZNP608*) for controls and probands.

Figure S2. ChIP-qPCR validation of 13 cohesin binding sites identified by ChIP array.

Figure S3. Cohesin binding within +/- 100 bp around TSSs is enriched in differentially expressed genes.

Figure S4. False-discovery rates (FDRs) of genome wide ChIP microarrays of (A) controls and (B) CdLS proband.

Table S1. Thirty nine training and testing samples were used for the whole genome expression array analyses.

Table S2. 1915 probe sets representing 1501 unique genes (FDR<0.05) are differentially expressed in CdLS.

Table S3. 339 non-redundant genes represented by 420 probe sets (FDR < 0.01) are differentially expressed in Cornelia de Lange syndrome.

Table S4. Evaluation of Leave-One-Out cross-validation for the 33 samples in the training set. Two healthy controls and 1 proband were misclassified.

Table S5. Five functional independent gene clusters identified among the 339 genes (FDR < 0.01) using GSEA online program and R code.

Table S6. 32 genes chosen by clustering based feature selection for custom array analysis.

Table S7. Cohort of 101 individuals of European descent selected for custom array validation. Clinical evaluation and gene mutations of this cohort are listed.

Table S8. 56 probes designed for the 32 selected genes the custom array.

Table S9. Step wise method to select the 23- and 10-gene classifiers and the 3-gene biomarkers.

Table S10. Intragenic cohesin binding in mapped human RefSeq genes.

Table S11. Specific primer pairs used for ChIP-qPCR validation.

Table S12. Intragenic cohesin binding in the classifier genes and the gene ontology analysis.

Table S13. Dys-regulated genes (FDR<0.05) identified in CdLS probands with *NIPBL* mutations that are functionally related to sister chromatid cohesion.

Supporting Information Methods - Statistical Analysis

a. Data process for Affymetrix expression arrays

1) Classification methods

2) Evaluation and comparison of methods

3) The procedure of Nearest Centroid classification (NC)

b. Principal component analysis (PCA)

c. Data process for custom arrays

d. Clustering-based feature selection

e. Integration of ChIP-on-chip and gene expression data

Supporting Information Figure Legends

Figure S1. Expression level of three genes (*NFATC2*, *PAPSS2* and *ZNP608*) for controls and probands. (A) 17 healthy controls and 14 severely affected CdLS probands with *NIPBL* protein truncating mutations, and (B) 101 sample cohort used for target array analysis including the same individuals as in (A). Three axes represent expression of the 3 genes, Blue dots represent controls, including healthy participants and individuals with other genetic diagnoses; red dots represent CdLS probands.

Figure S2. ChIP-qPCR validation of 13 cohesin binding sites identified by ChIP array. RAD21 ChIP samples were obtained from the CdLS proband and the control in the ChIP array studies and were analyzed by qPCR for the presence of 13 different cohesin-binding sites with site-specific primers (mean of n=3; error bars +/- standard deviation [SD])(see supplementary Table S11 for genomic addresses of the 13 sites and primer sequences). The results were presented as fold-enrichment over control ChIP (nonantibody). (A) The presence of cohesin binding at 13 examined genomic sites, sites #1 and #2 were bound equally by cohesin in both probands and control in the array studies and served as positive controls here; sites #3 and #4 did not demonstrate cohesin binding in either proband and or control in the array studies and served as negative controls here; sites #5 to #13 are 9 genomic sites where cohesin binding was lost in the CdLS cells by qualitative analysis in the array studies. Quantitative PCR has revealed the amount of cohesin bound to these sites is significantly reduced at all

of the examined loci. (B) Quantitative analysis of average amount of cohesin bound to the 9 examined sites revealed at least half of cohesin binding is lost in the CdLS cells.

Figure S3. Cohesin binding within +/- 100 bp around TSSs is enriched in differentially expressed genes. The 10,378 unique genes expressed in LCLs are ranked by their F scores. The reference enrichment is the overall percentage of genes having cohesin binding within 200bp (+/- 100 bp) around TSSs. The relative enrichment is calculated as the value of cohesin binding enrichment in top-ranked genes over the reference enrichment. The relative enrichment point is calculated for the total number of genes prior to the point on the x-axis. The numbers on x-axis denote the number of top-ranked genes. The curves are smoothed by the LOWESS algorithm.

Figure S4. False-discovery rates (FDRs) of genome wide ChIP microarrays of (A) controls and (B) CdLS proband. The x-axis denotes the *p*-values and the y-axis denotes the average FDR percentage for each experiment. Note that the FDR is less than 1% at the threshold *p*-value = 10^{-6} adopted for the analyses performed in this study.

Supporting Information Methods

Statistical Analysis

Data process for Affymetrix expression array

The Affymetrix .CEL files of all 39 arrays were processed by dCHIP (http://www.dchip.org) using PM-only background subtraction, invariant set normalization, and model-based probe set summarization. Probe set data was log2-transformed afterwards. The total of 54,675 probe sets was pre-filtered and the remaining 27,995 probe sets were averaged more than 5.467 (25% quantile) and called present in more than 40% of the 33 training arrays. These probe sets were considered as expressed in LCLs and used for the following analysis. We ranked the differential expression of probe sets between two training groups by calculating the ratio of between-group variance and pooled within group variance (the F statistic). Probe sets with higher F score were ranked higher. Instead of p values, we ran a permutation test to evaluate the significance of F scores. The 33 training samples were randomly re-grouped 100 times and the F score of each probe set was re-calculated each time. The False Discovery Rate (FDR) for each probe set was calculated as N/M, while N is the number of probe sets having equal or higher F scores in the permutated data, and M is the number of probe sets having equal or higher F scores in the real data. Probe sets having FDR less 0.05 were considered differentially expressed in control and CdLS samples. An even stricter cutoff of FDR less than 0.01 was also applied in some analyses. Summary of the statistic analyses is list in Supporting Information Methods Table 1.

Supporting Information Methods Table 1. Statistic procedures performed on microarray data analysis.

**Type of Analysis and Reporting ***
Outcome-related gene finding
Statistics used for comparison<br>F statistic<br>Assessment of statistical significance<br>False Discovery Rate<br>Method for controlling the number of false positives<br>Permutation test
Supervised prediction
Main model used for classification<br>Nearest centroid<br>Statistics used for feature selection<br>Combination of FDR, fold change, and functional relevance
Validation procedure
Use of a separate test set<br>Origin of test set : Random split on initial dataset<br>Size of test set: 6 samples<br>Ratio test/training set size: 6/33<br>Preliminary use of outcome information from test set samples : No<br>Use of expression data from test set samples for class definition : No
Cross-validation procedure<br>Preliminary use of outcome information from test samples : No<br>Use of expression data from test samples for class definition : No
Presentation of classifier performance
Prediction of diseased and control groups<br>Prediction accuracy or misclassification rate<br>Sensitivity and specificity<br>Positive apparent predictive rate and negative apparent predictive rate

* Table contents were adapted from the recommended microarray analysis guidelines by Dupuy and Simon [1].

<u>Classification methods</u>

A large number of classification methods, from naïve weighted voting method to complex machine learning technology, have been applied to class prediction of microarray samples. No method has been conclusively proven to be superior to the others and different experimental systems may have their own most suitable methods. We selected 7 classification methods commonly used in microarray studies and compared their performance on the 33 training samples (17 healthy controls and 16 severe CdLS) using leave-one-third-out validation procedure. Given that the number of variables is much larger than the number of samples, methods looking for optimal combination of predictive variables (genes), such as stepwise logistic regression, tend to over-fit the classifier. Consequently, we only selected methods assuming independence of predictive variables (genes) with

one exception: LDA (Linear Discriminate Analysis). All these methods except for the Score for Expression Profile (SEP) and Nearest Centroid (NC) were executed by previously implemented functions in R. The details about these methods are given in Supporting Information Methods Table 2.

Evaluation and comparison of methods

We performed a procedure of repeated resampling and leave-one-third-out cross-validation to evaluate and compare different classification methods. Two-thirds of the 33 samples (11 healthy controls and 11 severe patients) were randomly selected to train the classification models, and the remaining one-third of samples (6 controls and 5 patients) were used to test the models. The process of resampling followed by training-testing was repeated 100 times, so totally 1,100 (11 X 100) class predictions were made on the testing samples.

After each resampling, we calculated the differential expression of 27,995 probe sets between two training groups. The differential expression was represented by the ratio of between-group variance and within-group variance (F statistic). All the probe sets were then ranked by their F statistics, from high to low. The top-ranked N probe sets were selected as the input of classification models. We compared the results when N equal to 25, 50, 100, and 200. In almost all the cases, classification methods had the best performance when N was equal to 50, followed by 25, 100, and 200.

Supporting Information Methods Table 3 and Supporting Information Methods Table 4 summarize the performance of classification methods on 1,100 predictions when N is equal to 50. It shows that all classification methods perform similarly except LDA. The poorer performance of LDA is not surprising since it tends to over-fit the training data. The Nearest Centroid classification has an overall performance slightly better than the other methods. In addition, it is more robust to systematical bias and can report prediction result in continuous values instead of dichotomous classes. Nearest Centroid was then chosen as the preferred classification method in our experimental system. The detailed procedure of NC classification is given in the next section.

The procedure of Nearest Centroid classification (NC)

The basic idea of NC classification is to classify a testing sample to the class whose centroid is the nearest. Its procedure includes the following steps:

1. Select top-ranked N probe sets having the most prominent differential expression between 2 training classes, and use the data from these probe sets to generate centroids and predict testing samples.
2. Calculate the pooled standard deviation (SDp) of each selected probe sets in training samples, and divide the data of each probe set by its SDp. The purpose of this adjustment is to reduce the weight of high-variable genes in the classifier.
3. Calculate the median of each probe set using the adjusted training data, and generate an N-vector as the centroid for each class. The centroid can be

thought of as the middle point of training samples of the same class in an N-dimensional space, where N is the number of selected probe sets.

4. Given the adjusted data of each testing sample, calculate its distance to both centroids. The distance is measured by Pearson correlation coefficient instead of Euclidean because correlation is more robust to systematic bias between arrays.
5. Calculate a Discriminant Score (DS) for each testing patients:

   DS = 100*log2(corr2/corr1);   where corr1 and corr2 are the correlation coefficients to control and patient centroids, respectively.

   The transformation is to make the DS more readable and symmetric around 0. By default, a testing sample will be classified as a patient if its DS is greater than 0. However, the fact that DS is a continuous variable also makes it possible to compare distribution of DS between patient subgroups as we showed in the paper.

Supporting Information Methods Table 2. Classification methods

Method	Symbol	R package	R code
Linear Discriminant Analysis	LDA	MASS	> lda(train, label)→model<br>> predict(model, test) →prediction
Diagonal Linear Discriminant Analysis	DLDA	sma	> stat.diag.da(train, label, test) →prediction
Score for Expression Profile	SEP	[NA]	> sum(t*test) →SEP; (SEP>0)→prediction<br># t is the t statistic from 22 training samples
Naïve Bayes	NB	e1071	> naiveBayes(data.frame(train), label)→model<br>> predict(model, test)→prediction
Support Vector Machine	SVM	kernlab	> ksvm(train, label)→model<br>> predict(model, test)→prediction
Nearest Neighbor(s)	(k)NN	kknn	> kknn(label~., train, test, k)→prediction<br># k is number of neighbors to be considered
Nearest Centroid	NC	[NA]	See text for description

* In R codes, parameter 'train' stands for a matrix of 22 training samples and 'label' indicates the group of these samples (0 for healthy control and 1 for severe patient) while 'test' is the expression data vector of a testing sample.

Supporting Information Methods Table 3. Comparison of classification methods

Method	TP	FP	FN	TN	Sensitivity	Specificity	Accuracy	Odds Ratio	Kappa
**DLDA**	429	83	71	517	0.858	0.862	86.0%	37.427	0.718
**LDA**	415	124	85	476	0.830	0.793	81.0%	18.680	0.619
**SEP**	430	82	70	518	0.860	0.863	86.2%	38.585	0.722
**NB**	443	95	57	505	0.886	0.842	86.2%	41.063	0.723
**SVM**	438	94	62	506	0.876	0.843	85.8%	37.817	0.716
***7NN**	445	91	55	509	**0.890**	0.848	86.7%	**44.973**	0.734
**NC**	437	80	63	520	0.874	**0.867**	**87.0%**	44.817	**0.739**

TP: true positives; FP: false positives; FN: false negatives; TN: true negatives; Kappa: agreement between class prediction and diagnosed disease state.
* kNN classification has the best performance when k=7; see Supplementary Method Table 4 for full kNN results.

Supporting Information Methods Table 4. Performance of Nearest Neighbor classification with different values of k

k	TP	FP	FN	TN	Sensitivity	Specificity	Accuracy	Odds Ratio	Kappa
**1**	441	122	59	478	0.882	0.797	83.5%	29.153	0.672
**3**	445	108	55	492	0.890	0.820	85.2%	36.696	0.704
**5**	447	96	53	504	**0.894**	0.840	86.5%	43.987	0.729
**7**	445	91	55	509	0.890	0.848	**86.7%**	**44.973**	**0.734**
**9**	442	93	58	507	0.884	0.845	86.3%	41.298	0.725
**11**	443	94	57	506	0.886	0.843	86.3%	41.582	0.725
**13**	441	93	59	507	0.882	0.845	86.2%	40.511	0.723
**15**	439	91	61	509	0.878	0.848	86.2%	40.027	0.723
**17**	436	90	64	510	0.872	0.850	86.0%	38.394	0.719
**19**	435	88	65	512	0.870	0.853	86.1%	38.745	0.721
**21**	430	81	70	519	0.860	**0.865**	86.3%	39.133	0.724

Principal component analysis (PCA)

To better visualize differences in expression profiles between the CdLS probands and healthy control subjects. Spotfire Decision Site for Functional Genomics (version 8.0) was utilized to calculate the principal components on the training sets and then used the same principal components to analyze the test sets. We plotted the principal components which captured the most variation in the original data.

Data Process for custom arrays

101 LCL samples were measured on 105 (includes 4 technical replicates) custom arrays provided by Xceed Molecular Inc. ((http://www.xceedmolecular.com/). This custom array includes 56 probes whose sequences are mapped to 32 focused genes identified by genome-wide screening (see above). The array also includes 14 controls probes: 10 positive controls perfectly mapped to 10 housekeeping genes; 3 negative controls; and 1

mismatch probe of *ACTB*. These probes were used as references for data normalization. All arrays were processed together as below.

Raw values were log2-transformed after being adjusted by adding 10.0 to: 1) make all values positive and 2) reduce the variability of probes having low-level expression. A reference sample was created by averaging the values of each probe on all 105 arrays. Data of each array was normalized by using control probes to fit a robust linear regression model to the reference (R: rlm() function). Un-normalized data was subtracted by the intercept and then divided by the slope of the fitted line. Normalized data of technical replicates was averaged, so each unique sample got a single series of data.

If more than one probe was mapped to one focused gene, only the probe performing better in the 31 training samples would be used for analysis. The performance of probes was evaluated according to their intensity, difference between two training groups, and correlation to Affymetrix data.

All samples were Caucasian, but categorized into multiple groups with different clinical features and gene mutations. The details about sample groups are listed in the following Supporting Information Methods Table 5 and Supporting Information Methods Table 8.

Supporting Information Methods Table 5. 101 samples used for target array hybridization.

	Phenotype	NIPBL Mutation	#Samples
Original training	Healthy control	No	17
	Severe CdLS	Yes	14
New samples (with Caucasian background)	Healthy control	No	4
	Severe CdLS	Yes	6
	Moderate CdLS	Yes	9
	Mild CdLS	Yes	*26
	Moderate CdLS	No	4
	Mild CdLS	No, but SMC1A	9
	Mild CdLS	No	8
	Other disease	No	4

* 15 were randomly selected as training samples for a more robust classifier

Quality control

All 105 arrays show good and consistent data quality. The negative control probes have average intensity close to 0. Control probes of these arrays are highly correlated. Their Pearson correlation coefficients to the reference sample range from 0.9899 to 0.9999. Residuals of linear fitting of all control probes are symmetric around 0. The unsupervised hierarchical clustering of 31 training samples (on 35 arrays) shows that pairs of technical replicates are always the

nearest node of each other (Supporting Information Methods Figure. 1), suggesting that technical variance is generally smaller than biological variance.

Except for one gene (PCDHG), the selected probe of all focused genes is positively correlated to its counterpart on Affymetrix platform (r = 0.21 – 0.94) and significantly or marginally significantly different between 2 training groups (p = 0.06 – 6.7E-8).

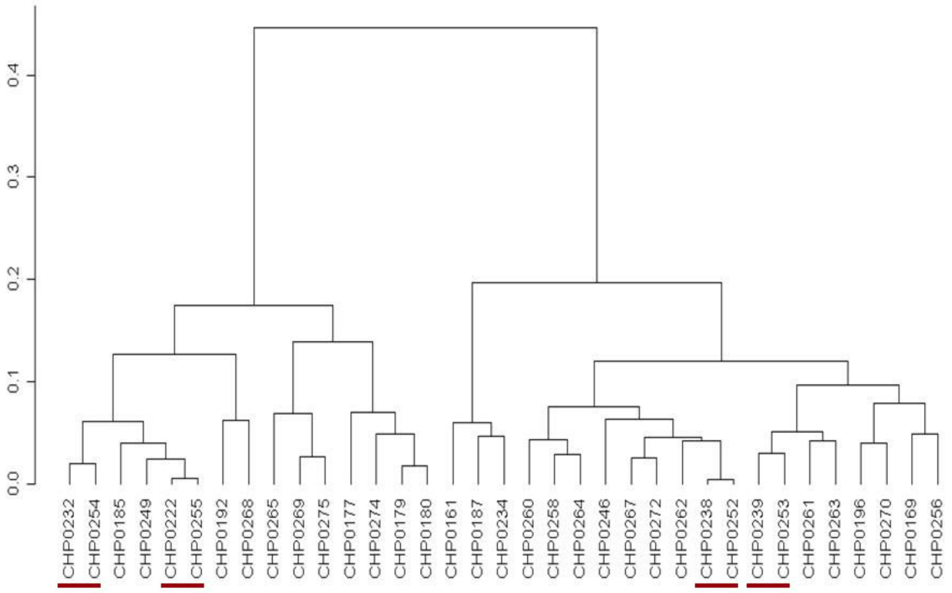

Supporting Information Methods Figure 1. Unsupervised hierarchical clustering of 31 training samples, with 4 pairs of technical replicates. Each pair of technical replicates (underlined) are clustered together.

<u>Identification and evaluation of multi-gene classifiers</u>

Expression values of some of the 32 selected genes are not significantly correlated between Affymetrix and Xceed platforms due to disparity of technologies and probe sequences. In addition, genes identified from control-versus-severe comparison may not be suitable to other disease subgroups. Consequently, we made further gene selection through a stepwise procedure (Supporting Information Methods Figure. 2). Different training proband samples were compared to healthy controls at each step and the classifiers identified were evaluated by remaining testing samples. All these 2-group comparisons were performed by Student’s *t* test assuming unequal variance.

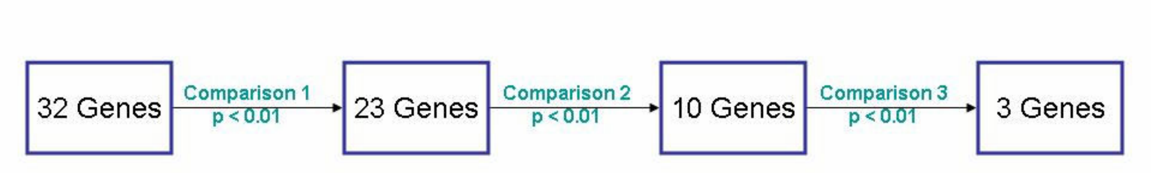

Comparison 1: 17 healthy controls *vs* 14 severe CdLS probands (original training samples)
Comparison 2: 17 healthy controls *vs* 15 mild CdLS probands with *NIPBL* mutations
Comparison 3: 17 healthy controls *vs* 17 mild CdLS probands without *NIPBL* mutation

Supporting Information Methods Figure 2. Stepwise selection of genes applicable to different CdLS subgroups using different training samples.

23 of the 32 genes are able to significantly differentiate between the two original training groups according to expressions on the target arrays (Comparison 1, Supporting Information Methods Figure 2). A classifier based on these genes and training samples was applied to all samples using Nearest Centroid method and a Discriminant Score (DS) was obtained for each sample (see above Comparison of Classification Methods for details). The performance of this 23-gene classifier on testing Caucasian samples is summarized in Supporting Information Methods Figure 3a:

1. The testing severe CdLS samples are ideally separated from controls, but less severe cases, especially those without *NIPBL* mutation, are not completely separated from controls.
2. There is a trend of gradual increase of group means, suggesting DS is correlated to severity of CdLS. If we code the subgroups 0 - 6 from left to right to indicate levels of disease severity, the Pearson correlation coefficient between DS and severity equals to 0.464 ($p = 0.0001$).
3. With DS=0 as cutoff, the classifier will misclassify most probands without an identifiable *NIPBL* mutation.

The poor performance of the 23-gene classifier on non-severe patients is because the genes were identified from severe *vs* control comparison. To improve the generality of classifier, we performed one more step of gene selection (Comparison 2, Supporting Information Methods Figure 2) by randomly selecting 15 mild cases with *NIPBL* mutation as a new training group. 10 of the 23 genes were significantly different between this group and original 17 normal controls. DS of samples was calculated again using these genes for Nearest Centroid classification. The performance of this 10-gene classifier is summarized in Supporting Information Methods Figure 3b:

1. Not only severe probands are completely separated from controls, but the mild and moderate cases are for the most part also separated.
2. The DS-severity correlation still exists.

3. The classifier performs well on all CdLS probands, especially cases with *NIPBL* mutations.
4. As a consequence of higher generality and less genes in the classifier, the within group variance of DS is increased in general.

According to previous results, classifiers identified from comparison of controls and mild cases without *NIPBL* mutation may have an even higher generality. However, only 3 genes, *NFATC2, PAPSS2* and *ZNP608*, showed a significant difference between the 17 controls and 17 mild cases without *NIPBL* mutation (Comparison 3, Supporting Information Methods Figure 2). The 2 mild subgroups were combined for this comparison to increase statistical power based on the observation that none of the 10 genes were different between mild cases with no mutation and with *SMC1A* mutations (*p* values from 0.14 to 0.94).

A

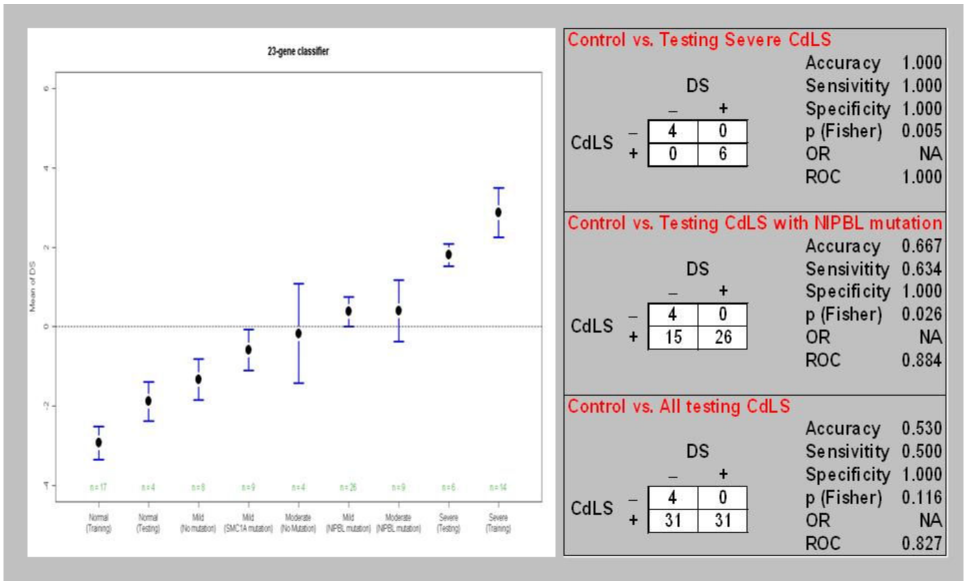

B

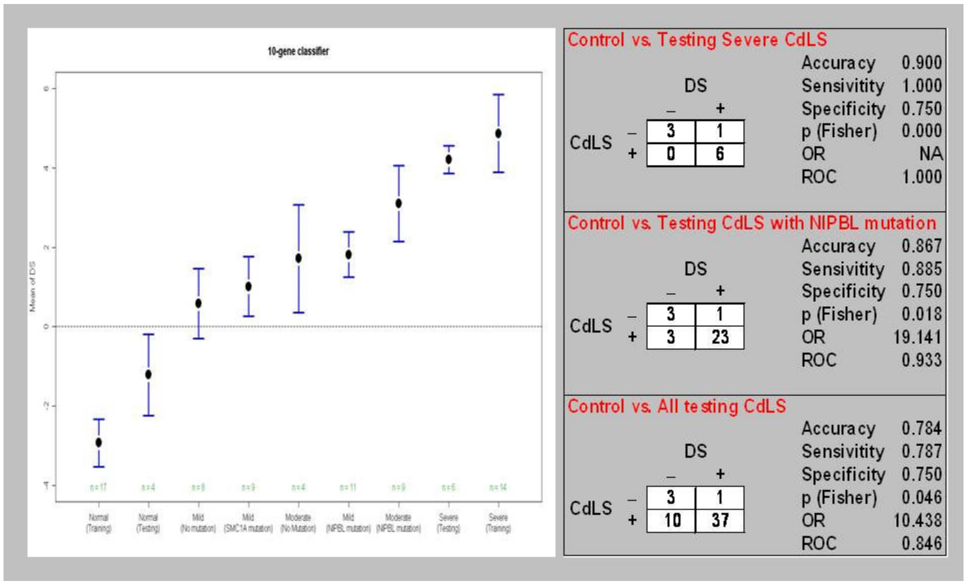

Supporting Information Methods Figure 3. Performance of multigene classifiers. The left side figures illustrate the distribution of DS of subgroups. Black dots indicate group means and vertical bars indicate standard errors. The right side panels summarize the performance of classifiers on different testing samples, using DS=0 as cutoff. OR: Odds Ratio; ROC: area of ROC curve of 2 classes (control *vs* CdLS); and *p*: Fisher's exact test (2-sided). (A) DS was calculated with a 23-gene classifier generated from healthy control *vs* severe CdLS comparison; (B) DS was calculated with a 10-gene classifier generated from healthy control *vs* mild CdLS (with *NIPBL* mutation) comparison. Note that the size of the mild with *NIPBL* mutation group was reduced to 11 in B. because the other 15 had been used as training samples.

Clustering-based feature selection

Clustering-based feature selection was carried out to select the signature genes for CdLS. Redundancy was collapsed for the previously defined 420 probe sets (with FDR <= 0.01) by keeping the ones with the highest F scores, and identified 339 unique genes. Normalization of each gene to a mean=0, S.D.=1.0, and using 'gap' statistic(Yeung et al. 2001) to obtain an optimal balance between the number of clusters and within-cluster variance (implemented by SAGx package in R) was undertaken. 32 focused genes were evenly chosen from 5 clusters in favor of higher F score (purely statistical), bigger fold change (more detectable), or less redundancy (consistent to the assumption about multiple pathways).

Intergration of ChIP-on-chip and gene expression data

Probe sets on expression microarrays were mapped to chromosome locations corresponding to the transcripts defined by the RefSeq Genes track of UCSC Genome Browser (http://genome.ucsc.edu, version hg18). Probe sets mapped to multiple locations were removed. If multiple probe sets were mapped to the same transcript, only the one having the greatest differential expression between

controls and probands would be used. Consequently, we obtained a total of 15,162 uniquely located transcripts whose expression was measured by the same number of probe sets. These transcripts were split into three groups: group A, not expressed in LCLs; group B, expressed in LCL without difference between controls and probands, and group C, differentially expressed between controls and probands (FDR < 0.05).

Cohesin binding sites in control and CdLS samples were identified from ChIP-on-chip data as described above. If a transcript or sequence (5'UTR, exon, etc.) overlapped with any binding site, we would call cohesin present in the transcript or sequence. The continuous frequency plots as in Figure 4C were generated by summarizing the presence of cohesin binding at a base location relative to the TSS or TTS.

References for Supporting Information Methods

1. Yeung KY, Fraley C, Murua A, Raftery AE, Ruzzo WL (2001) Model-based clustering and data transformations for gene expression data. Bioinformatics (Oxford, England) 17(10): 977-987.
